# The utility of endotracheal aspirate bacteriology in identifying mechanically ventilated patients at risk for ventilator associated pneumonia: a single-center prospective observational study

**DOI:** 10.1186/s12879-019-4367-7

**Published:** 2019-08-29

**Authors:** Ekaterina Kabak, Jana Hudcova, Zoltán Magyarics, Lukas Stulik, Marie Goggin, Valéria Szijártó, Eszter Nagy, Chris Stevens

**Affiliations:** 1 Previously Arsanis Biosciences GmbH, Vienna, Austria; 2 Independent Researcher, Vienna, Austria; 3 Lahey Hospital and Medical Center, Burlington, MA USA; 4 X4 Pharmaceuticals Inc, Cambridge, MA USA; 5 CEBINA (Central European Biotech Incubator and Accelerator) GmbH, Vienna, Austria; 6 Wilhelm-Weber-Weg 4/3/2, A-1110 Wien, Austria

**Keywords:** Ventilator-associated pneumonia, Endotracheal aspirate, *Staphylococcus aureus*, Gram-negative pathogens, Prospective study

## Abstract

**Background:**

Ventilator-associated pneumonia (VAP) is a well-known, life-threatening disease that persists despite preventative measures and approved antibiotic therapies. This prospective observational study investigated bacterial airway colonization, and whether its detection and quantification in the endotracheal aspirate (ETA) is useful for identifying mechanically ventilated ICU patients who are at risk of developing VAP.

**Methods:**

240 patients admitted to 3 ICUs at the Lahey Hospital and Medical Center (Burlington, MA) between June 2014 and June 2015 and mechanically ventilated for > 2 days were included. ETA samples and clinical data were collected. Airway colonization was assessed, and subsequently categorized into “heavy” and “light” by semi-quantitative microbiological analysis of ETAs. VAP was diagnosed retrospectively by the study sponsor according to a pre-specified pneumonia definition.

**Results:**

Pathogenic bacteria were isolated from ETAs of 125 patients. The most common species isolated was *S. aureus* (56.8%), followed by *K. pneumoniae*, *P. aeruginosa*, and *E. coli* (35.2% combined*)*. VAP was diagnosed in 85 patients, 44 (51.7%) with no bacterial pathogen, 18 associated with *S. aureus* and 18 Gram-negative-only cases, and 5 associated with other Gram-positive or mixed species. A higher proportion of patients who were heavily colonized with *S. aureus* developed VAP (32.4%) associated with *S. aureus* compared to those lightly colonized (17.6%). The same tendency was seen for patients heavily and lightly colonized with Gram-negative pathogens (30.0 and 0.0%, respectively). Detection of *S. aureus* in the ETA preceded *S. aureus* VAP by approximately 4 days, while Gram-negative organisms were first detected 2.5 days prior to Gram-negative VAP. VAP was associated with significantly longer duration of mechanical ventilation and hospitalization regardless of microbiologic cause when compared to patients who did not develop VAP.

**Conclusions:**

The overall VAP rate was 35%. Heavy tracheal colonization supported identification of patients at higher risk of developing a corresponding *S. aureus* or Gram-negative VAP. Detection of bacterial ETA-positivity tended to precede VAP.

**Electronic supplementary material:**

The online version of this article (10.1186/s12879-019-4367-7) contains supplementary material, which is available to authorized users.

## Background

Despite the approval of new antibiotics and the implementation of ventilator-associated pneumonia (VAP) prevention bundles, VAP remains a major concern and burden to the healthcare system, being associated with increased mortality, morbidity, hospital length of stay (LoS), and healthcare costs [1–4]. Several processes are implicated in VAP pathogenesis: aspiration of oropharyngeal secretions resulting from an altered state of consciousness, loss of natural protective mechanisms of the airways, and direct pathogen inoculation at the time of intubation [5]. Colonization of the airways by pathogens detected by routine bacterial cultures from endotracheal aspirate (ETA) may be useful to identify patients at increased risk for development of VAP [6].

In the case of VAP radiological and clinical signs, an ETA sample demonstrating high bacterial burden is considered confirmatory of microbial etiology according to recently issued guidelines from the Infectious Diseases Society of America [7]. *Staphylococcus aureus*, *Klebsiella pneumoniae*, *Pseudomonas aeruginosa*, *Acinetobacter baumannii*, and *Escherichia coli* are the most common pathogens associated with VAP [5]. These microorganisms are often multi-drug resistant (MDR) [8] and/or highly virulent, making early detection particularly important for initiating the appropriate treatment regimen [9–11].

The majority of reports focuses on the analysis of microbiologically confirmed VAP cases; less data are available on frequency and timing of airway colonization, and semi-quantitative culture methods, as well as their value for identification of patients at increased risk of developing VAP. The purpose of this study was to analyze semi-quantitatively cultured, serially collected ETA samples to better understand the association of bacterial colonization with progression to VAP in mechanically ventilated patients. We hypothesized that patients heavily colonized with pathogenic bacterial species in the trachea were more likely to develop culture-positive VAP during their ICU stay, and that heavier burden of colonization is associated with a higher risk of developing a bacterial VAP. VAP incidence in relation to bacterial airway colonization was examined as the primary outcome of the study. Secondary outcomes that were evaluated included the duration of mechanical ventilation (MV), ICU and hospital LoS, as well as all-cause mortality in patients with and without VAP.

## Methods

### Study population and data collection

A total of 250 mechanically ventilated patients admitted to two medical ICUs (MICU) and one surgical ICU (SICU) at the Lahey Hospital & Medical Center (Burlington, MA, USA) between June 2014 and June 2015 were included in this prospective observational study. Exclusion criteria included age below 18 years, and an expected length of MV of less than 48 h as judged by the treating physician (e.g., admission to the ICU from the operating theatre just for weaning, or moribund condition of the patient). Subjects exited the study upon extubation, tracheostomy, transfer to another facility, death, or initiation of “comfort measures only” protocol. Basic demographic data and the medical history were collected upon ICU admission from the patient directly, from the relatives with the help of collateral history taking, and/or from the available medical records. Hospital and ICU LoS, length of MV was recorded at the end of hospitalization, and diagnostic parameters, such as body temperature, white blood-cell count, and chest X-ray (CXR) readings were recorded daily during the ICU stay. Additionally, local epidemiology data on antibiotic susceptibility of the bacterial isolates, as well as empiric and prescribed antibiotic regimens were recorded.

All patient-related data were received in de-identified form. Informed consent was waived, since only leftover materials (otherwise discarded) were used, and no additional intervention or change in treatment plan was implemented. CXR was assessed by two independent, trained observers; in the case of non-unanimous judgement, the opinion of a senior radiologist was used as decisive. ETA samples were to be collected daily as part of the standard of care.

ETA samples were cultured and analyzed in the clinical microbiology laboratories of the Lahey Hospital and Medical Center. Bacterial species were determined by standard methods. Local rate of MDR pathogen isolation was not assessed separately for the ICUs included in the study. However, upon the commencement of the study global susceptibility rates of all isolates in the Lahey Hospital and Medical Center for the year preceding the study was provided. Multi-drug resistance was determined by demonstrating resistance to multiple antibiotic classes, with the conventional microbiological methods and Microscan® panels (latter used for all organisms except *P. aeruginosa*, where a disc diffusion test was employed), and/or detection of ESBL or carbapenemase activity. Alpha-hemolytic *Streptococcus* spp., apathogenic *Neisseria* spp., *Bacteroides* spp., *Fusobacterium* spp., *Spirochaetes*, and *Candida* spp. were considered as normal respiratory flora (NRF) [12]. Semi-quantitative microbiological analysis of ETA samples (SQ-ETA) was performed; samples were streaked onto appropriate agar culture plates in four consecutive quadrants. Presence of 5 or less colonies in the first quadrant corresponded to 1+ (rare), of 6 and more colonies in the first quadrant to 2+ (few), any amount of colonies in the first and second quadrants to 3+ (moderate), and in three consecutive or all 4 quadrants corresponded to 4+ (many). SQ-ETA data were used to categorize bacterial burden as light (1+ and 2+) or heavy (3+ and 4+). Heavy colonization corresponded to approximately ≥10^5^ CFU/mL of ETA [13]. Purulent ETA samples were detected by light microscopy and defined as > 10 polymorphonuclear cells per low power field. Pathogenic bacterial species recovered from the ETA were shipped to the sponsor, and additional analyses (species confirmation and MRSA status assignment by genetic tests) were performed as described previously [14].

The study was conducted in accordance with the ethical principles of the Declaration of Helsinki and the local regulations; and was approved by the Lahey Hospital and Medical Center Institutional Review Board.

### VAP diagnosis

Modified criteria outlined by Johanson et al. [15] were used for retrospective assignment of VAP by the sponsor and was defined by the presence of a new or progressive infiltrate determined by CXR, and at least two of the following clinical findings: (1) fever or hypothermia, (2) leukocytosis or leukopenia, and (3) presence of purulent respiratory secretions, all appearing > 2 days after initiation of MV. For this study, patients were defined as “progressing” to VAP if they had bacterial colonization and one or more episodes of VAP at any time during the study period. A bacterial pathogen was considered a potential causative agent of VAP if isolated from the ETA on the same day or within the 2 days preceding or following the detected of VAP clinical signs. In this study, both heavy and light ETA bacterial burden were accepted for determination of VAP bacterial etiology for the purpose of a more comprehensive overview. In selected patients, upon orders of the treating physicians, bronchoalveolar lavage (BAL) and/or non-bronchoscopic bronchoalveolar lavage (NBAL) was performed during the ICU stay. In order to avoid potential bias in this study, however, results of these tests were not included in the analysis, as they were not carried out routinely, were not limited to cases of suspected pneumonia (e.g. lung malignancy, interstitial lung disease) and were performed in less than 10% of the study population. No other nosocomial infections other than VAP was systematically assessed or recorded in this study.

Ventilator-associated tracheobronchitis (VAT) was not assessed as part of this study, as the authors believe that the retrospective diagnosis of VAT, in absence of documented radiological findings as in case of VAP, is most accurate when corroborated by the treating clinicians, which conflicted with the design of the study.

### Antibiotic use in the studied ICUs

Selective digestive decontamination (SDD) was not administered as part of routine patient care. Upon suspected VAP, cefepime and vancomycin was administered empirically; if a patient was found to be negative for *S. aureus*, vancomycin was removed from the regimen, and, depending on recovered bacterial flora, cefepime was continued or changed to ceftriaxone or cefazolin. In case of cephalosporin allergy levofloxacin was administered.

### Statistical analysis

Reported variables were grouped into continuous (e.g. age, MV duration, hospital LoS), and categorical values (e.g., gender, underlying disorders, all-cause mortality). Continuous variables were represented as mean with standard deviation, whereas categorical variables as percentage. Continuous variables were tested with Student’s two-tailed unpaired t-test; categorical ones were organized into contingency tables and tested with Fisher’s exact test. Statistical tests were performed with the Prism® 6.07 (GraphPad) software package. Level of statistical significance was set at *p* value ≤0.05.

## Results

### Patient demographics and ICU characteristics

Out of the 250 patients enrolled, 241 were mechanically ventilated for > 2 days; one additional patient was excluded due to missing clinical information crucial for analysis (Additional file 3: Fig. S1**,** Additional file 7). The average age among the remaining 240 patients was 64 years, and a slight male predominance was seen. The mean LoS and MV duration were 20 and 9 days, respectively. We addressed the potential differences in patient characteristics between those admitted to SICU and MICU, respectively. Significant differences were observed: patients in the SICU were hospitalized and ventilated significantly longer, but displayed significantly lower mortality rates compared to those in the MICU. Additionally, patients with respiratory failure were significantly more likely to be admitted to the MICU, whereas subjects requiring an emergency surgery or having a history of recent trauma – to the SICU, as expected by the ICU profiles. Other characteristics, including most frequently observed comorbidities, history of smoking, alcohol abuse, use of antibiotics and of immunosuppressive medications, did not differ significantly among the groups. VAP was detected in approximately one-third of the patients irrespective of the ICU type (Table 1).

**Table 1:** Patient demographics

Baseline variables*	All population (*n* = 240)	MICU (*n* = 138)	SICU (*n* = 102)	*p* value
Age, yr	63.9 ± 14.8	62.5 ± 14.7	65.9 ± 14.7	0.0752
BMI	30.2 ± 9.5	30.1 ± 9.3	30.4 ± 9.7	0.7875
Hospital LoS	19.6 ± 13.3	16.5 ± 10.4	23.8 ± 15.4	< 0.0001
Duration of MV	9.1** ± 5.1	8.5 ± 5.1	9.8** ± 5.1	0.0482
Male, % (*n*)	58.8 (141)	58.0 (80)	59.8 (61)	0.7923
All-cause mortality, % (*n*)	31.3 (75)	39.9 (55)	19.6 (20)	0.0062
SICU, % (*n*)	42.5 (102)	N/A	N /A	N/A
Emergency surgery, % (n)	30.4 (73)	8.0 (11)	60.8 (62)	< 0.0001
Trauma, % (*n*)	9.2 (22)	2.9 (4)	17.6 (18)	0.0001
Hypertension, % (*n*)	50.4 (121)	48.6 (67)	52.9 (54)	0.5165
Diabetes, % (*n*)	27.5 (66)	29.0 (40)	25.5 (26)	0.5627
Asthma, % (*n*)	7.5 (18)	8.0 (11)	6.9 (7)	0.8088
COPD, % (*n*)	17.1 (41)	16.7 (23)	17.6 (18)	0.8636
Chronic heart failure, % (*n*)	16.3 (39)	19.6 (27)	11.8 (12)	0.1146
Malignancy, % (*n*)	23.8 (57)	21.0 (29)	27.5 (28)	0.2837
Previous smoker, % (*n*)	26.7 (64)	26.1 (36)	27.5 (28)	0.8829
Current smoker, % (*n*)	18.3 (44)	21.7 (30)	13.7 (14)	0.1303
Alcohol abuse, % (*n*)	23.3 (56)	25.4 (35)	20.6 (21)	0.4416
Antibiotics in the last 90 days, % (*n*)	43.3 (104)	34.1 (47)	46.1 (47)	0.1774
Antibiotics upon admission, % (*n*)	15.0 (36)	15.9 (22)	13.7 (14)	0.7161
Immunoactive drugs***, % (*n*)	22.1 (53)	24.6 (34)	18.6 (19)	0.3450
Admission due to sepsis, % (*n*)	17.1 (41)	19.6 (27)	13.7 (14)	0.2982
Admission due to respiratory failure or hypoxia, % (*n*)	27.9 (67)	44.2 (61)	5.9 (6)	< 0.0001
VAP, % (*n*)	35.4 (85)	31.9 (44)	40.2 (41)	0.2193
*S. aureus* VAP, % (*n*)	7.5 (18)	5.8 (8)	9.8 (10)	1.000
Gram-negative VAP, % (*n*)	7.5 (18)	6.5 (9)	8.8 (9)	0.6215

*Results are presented as mean ± standard deviation for numerical, and as percentage with an absolute count (n) for categorical variables. For comparison of MICU and SICU groups, *p*-values were determined by unpaired two-tailed t-test for numerical data and two-tailed Fisher’s exact test for categorical data. N/A = not applicable. **1 patient excluded since MV start date was not recorded. ***Including corticosteroids and chemotherapy; upon admission and/or in the last 90 days

Few clinical indicators such as clinical pulmonary infection score (CPIS) and disease severity scores could not be analyzed as some of the clinical parameters required for their calculation were not available, as they were not recorded for every patient, or were recorded at different time points during the ICU stay.

### Bacterial pathogens isolated from the ETA samples

**Fig. 1:**
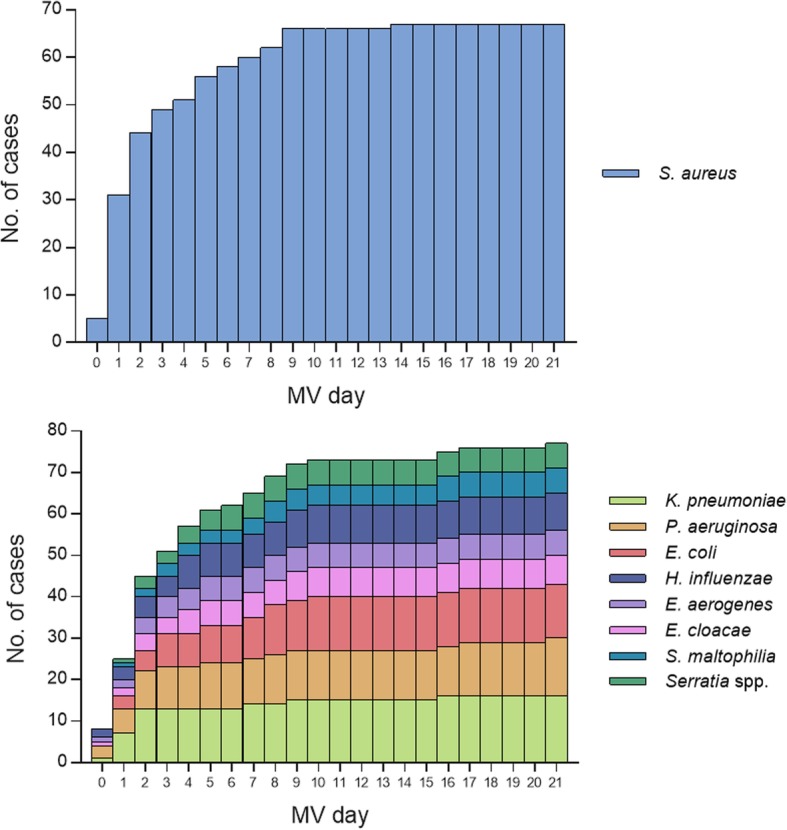
First detection of *S. aureus* and the most common Gram-negative pathogens in ETA samples. 5 patients were excluded (no ETA collected during the first 3–5 days of MV or no MV start date recorded)

Upon study commencement the most recent, yearly assessed global rates of local bacterial susceptibility to the antibiotics were provided, summarized and adapted for the purposes of the study to the organisms most commonly isolated from the ETA in Additional file 1: Table S1. ETA sampling frequency was approximately 2 out of every 3 MV days in the overall ICU population studied. The ETA microbiological culture results for patients were grouped into three main categories: yielding *S. aureus* (*n* = 71), Gram-negative bacteria (18 species; *n* = 75), or displaying no bacterial growth or only NRF (*n* = 115). 35.0% of the study subjects (84/240) yielded a single pathogen throughout the study, whereas 12.9% had two, and 4.2% yielded three or more pathogens. The most common bacterial species in ETAs was *S. aureus*, isolated from 29.6% of the study patients (71/240), and 56.8% (71/125) of patients with pathogen-positive ETAs (Additional file 9). 40.8% (29/71) of *S. aureus* positive patients carried MRSA. This was similar to the global MRSA rate (38%) reported for Lahey Hospital and Medical Center in the year preceding the study (Additional file 1: Table S1). The three most commonly detected Gram-negative pathogens, *K. pneumoniae*, *P. aeruginosa* and *E. coli,* were present in ETA samples of 18.3% (44/240), and 15 other Gram-negative species were detected in 22.1% (53/240) of patients (Table 2). 47.8, 43.8, and 42.7% of *S. aureus*, *K. pneumoniae*, and *P. aeruginosa* respectively were detected in the ETA by day 2 of MV. *E. coli*, *S. maltophilia*, *E. cloacae*, and *E. aerogenes* appeared later in the course of MV, with only 17–33% of these species isolated within the first two MV days (Fig. 1).

**Table 2:** Bacterial pathogens isolated from ETA

Bacterial species	*n*	% of all subjects (*n* = 240)	% of subjects carrying pathogen in ETA (*n* = 125)
*Staphylococcus aureus*	71	29.6	56.8
MRSA*	29	12.1	23.2
MSSA*	43	17.9	34.4
*Klebsiella pneumoniae*	17	7.1	13.6
*Pseudomonas aeruginosa*	14	5.8	11.2
*Escherichia coli*	13	5.4	10.4
*Haemophilus influenzae*	9	3.8	7.2
*Enterobacter cloacae*	7	2.9	5.6
*Enterobacter aerogenes*	6	2.5	4.8
*Stenotrophomonas maltophilia*	6	2.5	4.8
*Serratia marcescens*	5	2.1	4.0
*Klebsiella oxytoca*	4	1.7	3.2
Diphtheroid	4	1.7	3.2
*Burkholderia cepacia*	3	1.3	2.4
*Moraxella* spp.	3	1.3	2.4
*Achromobacter xylosoxidans*	2	0.8	1.6
*Streptococcus pneumoniae*	2	0.8	1.6
*Acinetobacter baumannii*	2	0.8	1.6
β-hemolytic Group F Streptococci	2	0.8	1.6
*Proteus mirabilis*	2	0.8	1.6
*Serratia rubidaea*	1	0.4	0.8
*Acinetobacter lwoffii*	1	0.4	0.8
*Alcaligenes denitrificans*	1	0.4	0.8
*Streptococcus agalactiae*	1	0.4	0.8
β-hemolytic Group C Streptococci	1	0.4	0.8
*Citrobacter freundii*	1	0.4	0.8

*Two subjects carried both MRSA and MSSA simultaneously. One of the 71 patients with *S. aureus* positive ETA excluded as the isolate was not tested for antibiotic susceptibility

### ETA microbiology during VAP events

**Fig. 2:**
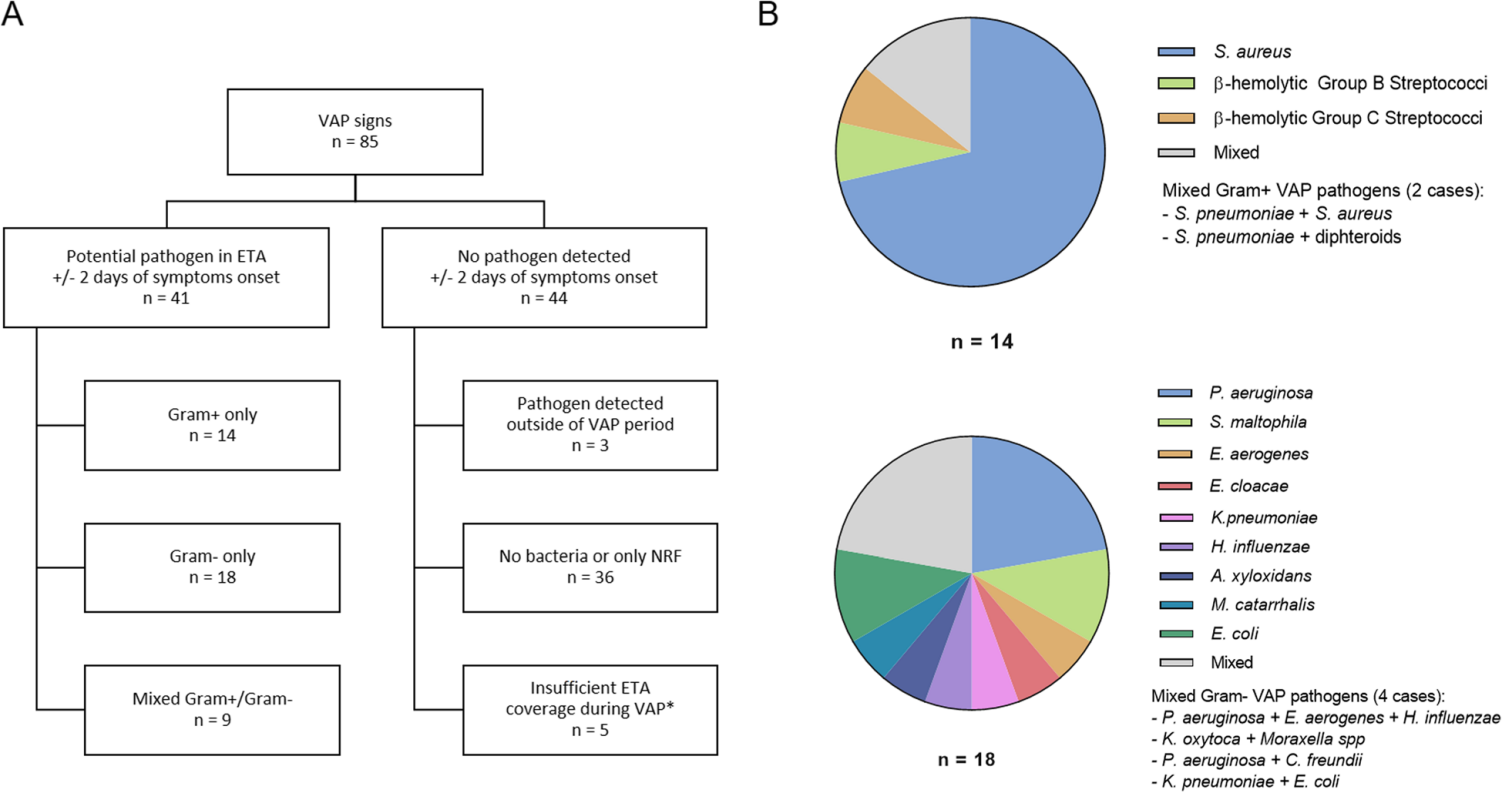
ETA bacteriology and its association with VAP. **a**: Bacteriological characteristics of ETA samples obtained in the VAP relevant-period (defined as day where VAP was diagnosed ±2 days). *not sufficient ETA sample coverage: ETA samples obtained on < 50% of days during VAP-relevant period and not yielding any pathogens. **b:** Gram-positive and Gram-negative species isolated from ETAs in VAP-relevant period. All cases where multiple bacterial species were isolated are listed individually

**Fig. 3:**
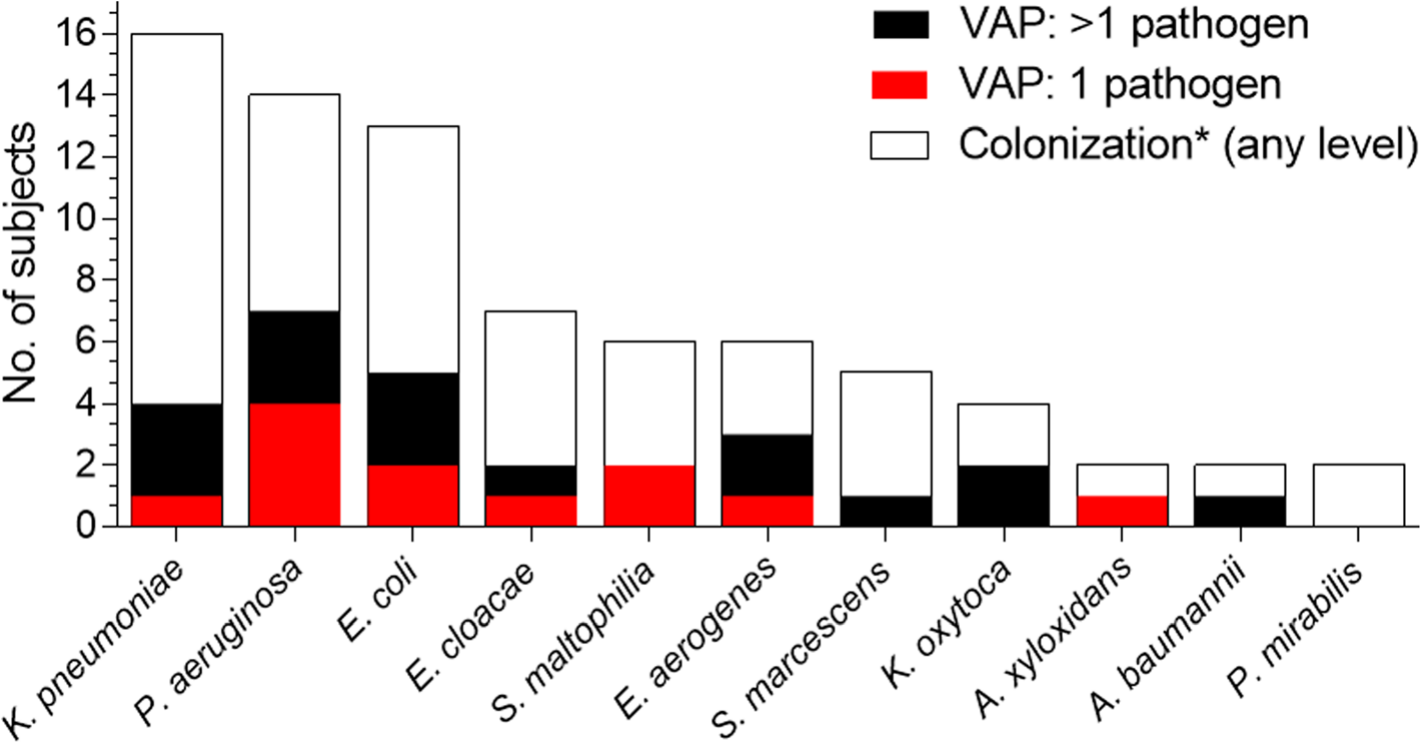
Role of Gram-negative organisms in VAP and colonization**.** *Does not exclude VAP-positive subjects with no pathogens isolated in VAP-relevant period

Eighty-five of the 240 study patients experienced VAP during MV (Additional file 8). In 41 (48.2%) of these patients, potential pathogenic bacteria were recovered from the ETA samples at the time of VAP diagnosis, ±2 days **(**Fig. 2A**)**. *S. aureus* was isolated from 18 patients; however, in 7 of these cases Gram-negative pathogens, and in one case *S. pneumoniae* were also isolated (Fig. 2B, Additional file 2: Table S2**,** Additional file 4: Fig. S2**,** Additional file 5: Fig. S3). Twenty-seven patients (65.8%) had at least one Gram-negative pathogen, the most common ones being *P. aeruginosa*, *E. coli*, and *S. maltophilia* (Fig. 3 and Additional file 2: Table S2).

Both medical and surgical ICU had similar proportions of *S. aureus* and Gram-negative VAP cases (Table 1). In 51.8% of the VAP patients, no culturable bacteria (other than NRF) were detected in the ETA (Fig. 2A).

### Association of airway colonization with subsequent pneumonia

**Fig. 4:**
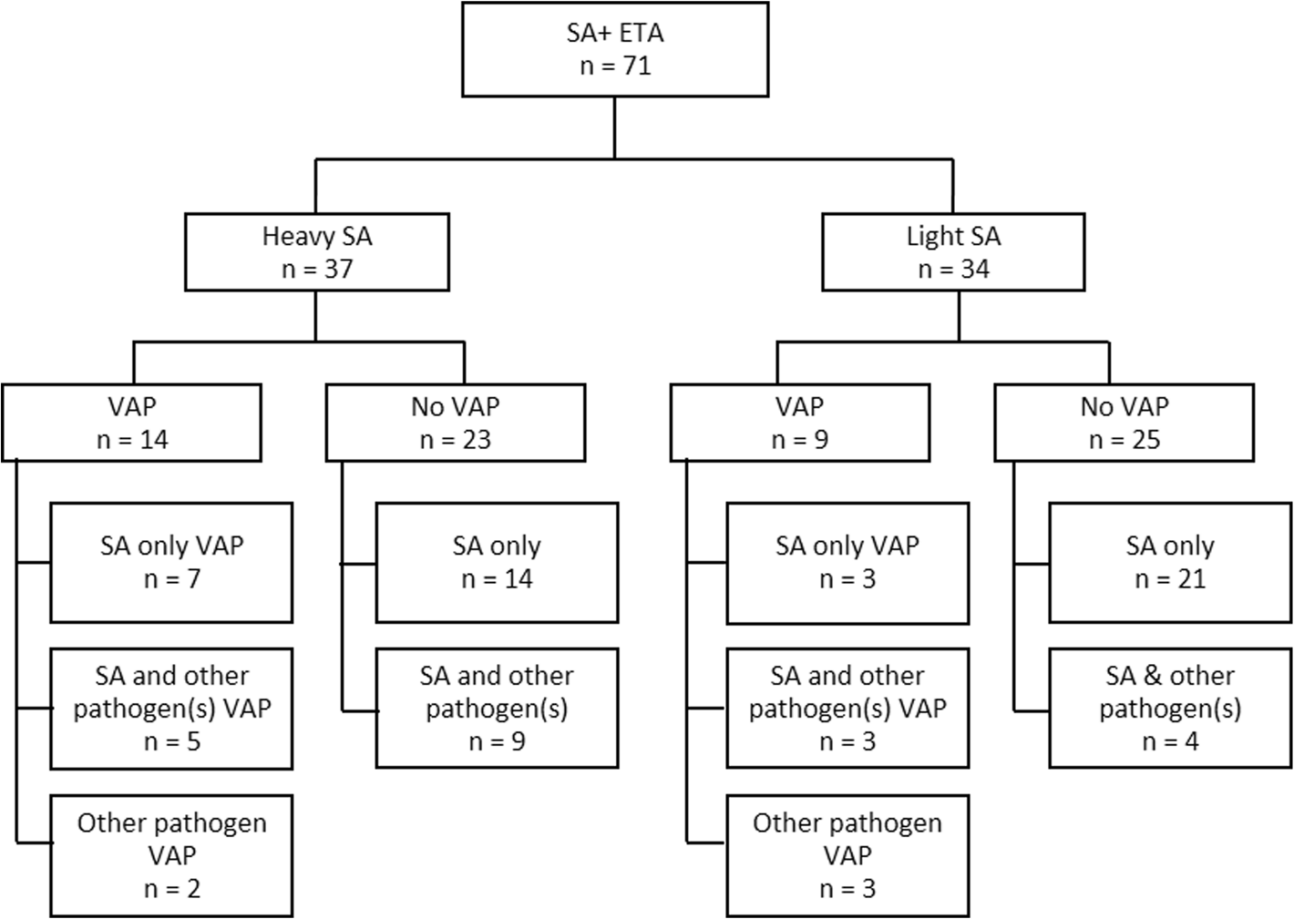
VAP in patients demonstrating a heavy or low *S. aureus* burden in ETA. SA = *S. aureus*

Patients yielding ETA samples with *S. aureus* and Gram-negative (pooled) organism were divided into heavily or lightly colonized. Seventy-one of the 240 patients had at least one ETA sample positive for *S. aureus*: 37 were heavily colonized and 34 were lightly colonized. 12 of the 37 heavily colonized patients (32.4%) developed VAP associated with *S. aureus* during the VAP-relevant period, compared to only 6 out of 34 lightly colonized patients (Fig. 4). The difference between progression to VAP from heavily colonized (32.4%) compared with lightly colonized (17.6%) did not reach statistical significance, possibly due to low sample size (*p* = 0.1808). Notably, out of the 18 total VAP cases with *S. aureus* present 15 yielded at least one ETA with only *S. aureus* at any time during the VAP-relevant period.

Of the 71 patients yielding *S. aureus* positive ETAs, 41 had MSSA, 27 MRSA, 2 had both, and for 1 patient the isolate was not received and its methicillin resistance could not be assessed. 48.6% (18/37) of the *S. aureus* from ETAs with heavy colonization were MSSA, and 67.6% (23/34) with light colonization were MSSA. Patients heavily colonized only with MSSA progressed to VAP in 44.4% (8/18) of cases, while only 23.5% of patients heavily colonized with MRSA did so (4/17, *p* = 0.289). In total, MSSA was implicated in 11 out of 18 VAP cases with *S. aureus* present, and 7 out of 10 where only *S. aureus* was isolated during the whole VAP-relevant period. Notably, in all *S. aureus* VAP patients colonizing and VAP strains had the same methicillin resistance status.

**Fig. 5:**
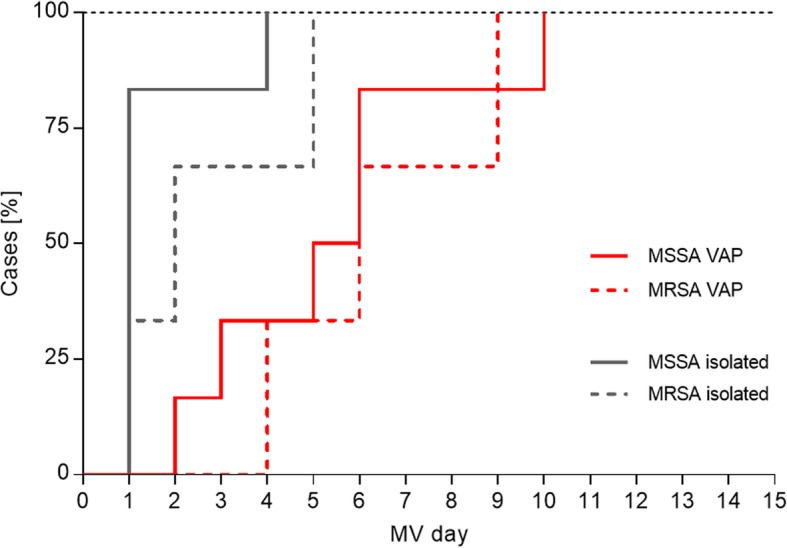
First detection of *S. aureus* lower airway colonization and progression to *S. aureus* VAP. Cumulative curves of first *S. aureus* ETA colonization detection and first day of *S. aureus* monomicrobial VAP diagnosis shown against MV days. One of 10 *S. aureus* VAP subjects was excluded: no ETA was collected during the first 5 days of MV

The first isolation of *S. aureus* in ETA occurred early during MV (median: 2 days) (Fig. 1), and no temporal difference was observed between MRSA and MSSA carrying patients. In patients who later developed *S. aureus*-only VAP (9 patients: 10 total, and 1 excluded due to insufficient ETA coverage prior to VAP event), detection of *S. aureus* in the ETA preceded VAP clinical symptoms by a median value of 4 days. Of these 9 patients, only 1 did not have an ETA positive for *S. aureus* prior to the *S. aureus*-only VAP event (Fig. 5**,** Additional file 4: Fig. S2).

Out of the 75 patients with ETA samples colonized with any Gram-negative pathogen, 60 were heavily colonized and 15 were lightly colonized. Eighteen of the 60 Gram-negative heavily colonized progressed to Gram-negative VAP (30.0%), while none (0.0%) of the 15 lightly colonized did so (*p* = 0.0156) (Fig. 3). In 94.4% (17/18) of Gram-negative VAP cases patients were colonized with at least one of the species causing the infection. On average, Gram-negative organisms were first detected in the ETA 2.5 days (median value) before the onset of VAP.

### Impact of VAP on duration of ventilation, hospital stay and mortality

**Fig. 6:**
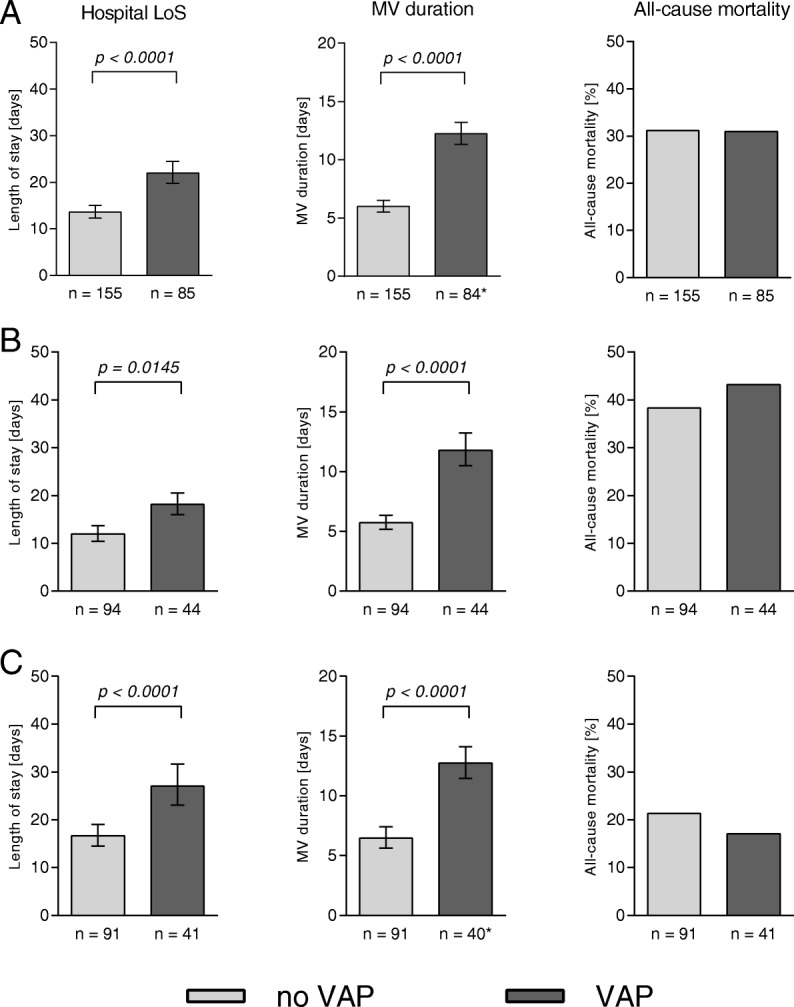
Impact of VAP on hospital LoS, duration of MV, and all-cause mortality in study population. **a:** All subjects; **b:** subjects admitted to the medical ICUs; **c:** subjects admitted to the surgical ICU. Geometric mean +/− 95% confidence interval is shown. Significance, calculated by two-tailed unpaired t-test (hospital LoS, MV duration) or with Fisher’s exact test (mortality) is indicated when *p*-value is < 0.05. *1 patient excluded since MV start date was not recorded

**Fig. 7:**
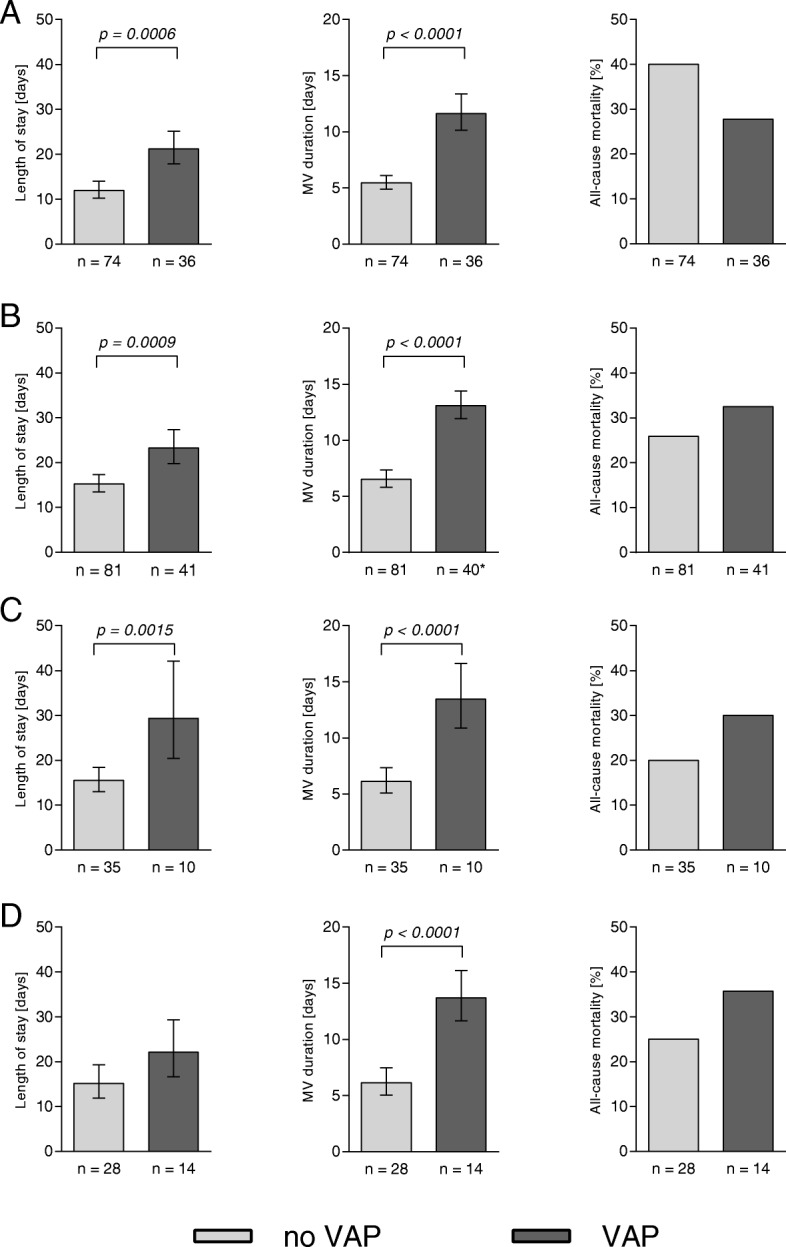
Impact of VAP caused by different pathogens on hospital LoS, duration of MV, and all-cause mortality. **a:** Subjects with no bacteria recovered or only NRF in the ETA. 5 patients with insufficient ETA coverage during VAP, and 3 patients colonized with bacterial pathogens outside of VAP-relevant period, are excluded; **b:** subjects with any pathogenic species in the ETA; **c:** subjects positive for *S. aureus* in ETA, monomicrobial only; **d:** subjects positive for Gram-negative species in ETA, monomicrobial only. Geometric mean +/− 95% confidence interval is shown. Significance, calculated by two-tailed unpaired t-test (hospital LoS, MV duration) or with Fisher’s exact test (mortality) is indicated when p-value is < 0.05. *1 patient excluded since MV start date was not recorded

Regardless of presence of ETA colonization, patients with at least one VAP episode required MV support and hospital care for twice as long (mean 13.0 and 16.6 days, respectively) as those who did not develop VAP (mean 6.9 and 25.1 days) (Fig. 6A). This trend was seen for all VAP patients regardless of the type of ICU (medical or surgical) (Fig. 6B-C), pathogen presence and causative bacterial species (Fig. 7A-D), with the exception of the Gram-negative VAP group, where no significant difference in hospital LoS was seen. Although statistical significance was not reached, notable observations regarding all-cause mortality were made. Whereas overall mortality rate between VAP and non-VAP patients were similar (Fig. 6A**)**, an increase of approximately 10% was seen in VAP patients whenever a pathogen was implicated (Fig. 7B-D). This was not true for the bacteria-negative/NRF VAP group, where VAP was paradoxically associated with a 10% decrease in mortality.

Interestingly, a temporal difference was observed between bacterial pathogen-positive VAP peaking in November–December, and bacterial pathogen-negative ones in the January–February period (Additional file 6: Fig. S4).

## Discussion

In this study, we investigated the microbiology of upper airway colonization in ICU-admitted mechanically ventilated patients. We noted significant differences in MV duration, hospital LoS and mortality between the patients admitted to the surgical or medical ICUs. Similar observations on mortality rates have been reported [16]. Importantly, there were no significant differences in basic demographic parameters (age, gender, BMI, etc.) and in the clinical variables (comorbidities, smoking and alcohol history, etc.). This supports the generalization of our findings on the airway bacterial colonization and VAP incidence to the studied patient population. Approximately one third of all patients developed VAP. When assigning a pathogen to a VAP event, a two-day period prior to the clinical diagnosis has been suggested as clinically appropriate [17]. Patients categorized by ETA bacteriological analysis results as *S. aureus*-positive, yielding Gram-negative organisms, or no bacterial growth/NRF, all developed VAP at about the same rate, 25, 24, and 31%. The principal pathogens implicated in VAP as shown in this study are consistent with universally reported species [9, 10, 18], as is the proportion of VAP without bacterial association [2].

*S. aureus* was the most prominent individual pathogenic species isolated. Higher prevalence of *S. aureus* VAP in the United States compared to Europe has been widely published [19]. The *S. aureus* tracheal colonization rate (~ 30%) from this study was similar to that reported 5 years earlier in the same ICUs [14]. Data on the temporal pattern and onset of *S. aureus* tracheal colonization is limited; however, available studies report both MSSA and MRSA colonization appearing early during the MV period [20–22]. There was a tendency for a higher MSSA-to-MRSA ratio in VAP patients when compared to the ratio of patients colonized without pneumonia, which is consistent with our previous observation, and is in line with reports of MRSA isolates characterized with higher persistence, but lower virulence [14, 23]. Nearly half of the *S. aureus*-positive ETA samples were detected within the first two days of mechanical ventilation, irrespective of methicillin resistance.

A major focus of this study was to evaluate whether differences in semi-quantitative bacterial ETA colonization could support the identification of patients who would more likely progress to VAP with the colonizing bacteria. We found that high ETA bacterial burden increases the likelihood of progression to bacterial VAP, as demonstrated by a significantly higher proportion of patients heavily colonized by Gram-negative bacteria progressing to VAP (30.0%) compared to those lightly colonized (0%). For *S. aureus* this difference did not reach statistical significance, although heavily colonized patients still progressed to *S. aureus* VAP more frequently (32.4%) than lightly colonized (17.6%). The detection of *S. aureus* in ETA preceded the onset of VAP by approximately 4 days, offering clinicians time to identify and characterize the bacterium and apply prophylactic measures. For Gram-negative bacteria, however, this period was shorter, only 2.5 days. Although antibiotic intervention is not recommended as prophylaxis of VAP and has shown to be ineffective [24], the early choice of appropriate antibiotic therapy is expected to reduce LoS, MV duration, and mortality [25, 26]. Moreover, several pathogen-specific non-antibiotic approaches (such as monoclonal antibodies) are being evaluated in preemptive settings, mainly targeting *S. aureus*, but also MDR Gram-negative pathogens [27, 28]. Identifying patients at increased risk of pneumonia allows to focus clinical trial study populations on those patients that would most likely benefit from preemptive intervention.

We observed that heavy colonization by *S. aureus* based on the bacterial load in the ETA was associated with numerically higher VAP rate (32.4%) compared to light colonization (17.6%), when the ETA was also positive during the VAP event. However, similarly high rate (31.3%) of VAP was detected among those patients whose ETA samples were negative for potential bacterial species before VAP diagnosis. This was a surprising finding, and calls for possible explanations. For this study, only ETA samples were considered in assigning a bacterial pathogen to a VAP episode, and the information on deep respiratory sample (such as bronchoalveolar lavage or a protected specimen brush) microbiology was not included. This constitutes one of the limitations of the study, and may explain some of VAP episodes with no causative bacterial species identified. At the same time, apart from technical reasons (e.g., low bacterial inoculum size due to successful antibiotic therapy, suboptimal sampling), this may also be linked to non-bacterial pathogens such as viruses or fungi, not routinely cultured organisms (such as *Legionella pneumophila*), as well as pathogens not covered by the standard microbiological culture methods [5]. Some reports state that as many as 56% of the pathogens causing VAP were not identified by the standard microbiological methods, and suggested a higher complexity of VAP microbiology than currently thought [29]. Additionally, non-infectious lung diseases, and diseases with secondary lung involvement mimicking pneumonia need to be considered. A critically ill, multimorbid patient with signs of infection and chest X-ray infiltrate, but negative bacterial culture of airway secretions presents a diagnostic challenge, and requires a careful diagnostic workup by the treating physician to consider other conditions with clinical presentation similar to VAP [30]. Microbiologically non-confirmed VAP remains under-represented in the literature and needs to be addressed by further research, including.

In one fifth of all monomicrobial VAP cases, pathogens were present at low abundance (1+ or 2+ SQ-ETA) during the VAP-relevant period. Along with the scarce presence of bacterial cells in the respiratory tract, such results can also be explained by suboptimal or difficult sampling, or by the low number of viable bacterial cells as a result of antibiotic therapy, which is frequent in the ICU population. There is no consensus on how to interpret such cases. While targeted antibiotic therapy for VAP is only recommended after confirmatory qualitative respiratory sample with bacterial counts at or above diagnostic threshold [7], lower bacterial burden associated with VAP in certain patients (high clinical suspicion, deteriorating patient, absence of other infection source) cannot be ignored.

Other significant observations included hospital length of stay, time on mechanical ventilation, mortality, and time to onset of VAP. Importantly, significant differences in hospital LoS and MV duration were observed between patients with and without VAP, regardless of the detectability of bacterial pathogens in ETA samples. No significant differences were seen in all-cause mortality as a consequence of VAP, although there was a trend for approximately 10% higher mortality when an ETA bacterial pathogen was detected, and a 10% lower mortality when no bacterial cause was identified. Previous studies on bacterial VAP also reported approximately two-fold increase in MV duration and hospital LoS and significant mortality difference of ~ 10% (attributable mortality) [1, 3, 31]. We observed a temporal difference between the bacterial pathogen-positive and -negative VAP groups, with the former being detected in autumn, and the latter peaking in the winter period, indicating possible seasonality of both groups.

The limitations of the present study need to be acknowledged. First, although data and samples were prospectively collected, diagnosis of VAP was assigned retrospectively by the sponsor based on generally accepted criteria and not by the treating physicians’ diagnosis. Second, the relatively small sample size, confined to a single medical center, limits the generalization of our data to other centers and ICUs. Moreover, analysis of the contribution of individual bacterial species other than *S. aureus* to VAP is limited due to low case number.

## Conclusions

Currently available VAP prevention measures, while effective [4] are suboptimal and may be time consuming for nursing staff [32]. Antibiotic therapy has not demonstrated an effect on VAP prevention and therefore it is not recommended. Continuous SQ-ETA surveillance cultures from the start of mechanical ventilation for those patients expected to be ventilated for more than 2 days to identify bacterial pathogens may be useful to identify patients at high risk for bacterial VAP infection. Moreover, tracheal colonization precedes the clinical signs and symptoms of pneumonia by several days on average providing a window of opportunity for VAP prevention. We found that approximately every third patient with heavy *S. aureus* or Gram-negative colonization in SQ-ETA progressed to VAP with a corresponding pathogen. At the same time, only every sixth patient lightly colonized with *S. aureus* progressed to VAP, and no patient with Gram-negative light colonization did so. This study provides prospective data to support ongoing efforts to identify high risk bacterial colonized patient populations to target and focus pneumonia preventive measures [33].

## Additional files

Additional file 1: **Table S1.** Antibiotic susceptibility of pathogenic bacterial species isolated in Lahey Clinic**.** Global antibiotic susceptibility rates are depicted for the bacterial species most commonly (≥2.5% of all ETA species) isolated from the ETA. Data was collected in the period between 01.01.2013 and 31.12.2013; only 1 isolate per patient and only the first isolate was considered. (DOCX 15 kb)

Additional file 2: **Table S2.** Species isolated from ETA samples of patients with mixed Gram-positive and Gram-negative VAP episodes. (DOCX 13 kb)

Additional file 3: **Figure S1.** Disposition of the patients included in the analysis. (PDF 181 kb)

Additional file 4: **Figure S2.** Patients with *S. aureus* monomicrobial VAP episodes. *S. aureus* burden dynamics (shown as SQ-ETA readout) and VAP clinical diagnosis days with VAP-relevant period highlighted. Only those days when ETA was obtained and analyzed are shown on X-axis. (PDF 80 kb)

Additional file 5: **Figure S3.** Patients with *S. aureus* mixed infection VAP episodes. *S. aureus* and other pathogens are indicated as SQ-ETA readout and VAP clinical diagnosis days with VAP-relevant period highlighted. Only those days when ETA was obtained and analyzed are shown on X-axis. (PDF 74 kb)

Additional file 6: **Figure S4.** Temporal distribution of VAP cases during the study period. **A:** Number of cases detected shown against corresponding time period. **B:** Number of cases normalized against number of recruited patients in the corresponding time period. Study commenced in the middle of June 2014; therefore VAP cases were counted at intervals from 17th of the month 16th of the subsequent month. In the December–January period no bacterial VAP cases were observed, despite active recruitment of patients in the study. (PDF 22 kb)

Additional file 7: Demographic characteristic of the study population. (PDF 584 kb)

Additional file 8: Daily observations, VAP diagnosis and ETA culture results. (PDF 109 kb)

Additional file 9: Methicillin resistance of the received *S. aureus* isolates. The data for the entire study population presented. (PDF 34 kb)

## Data Availability

All data generated or analysed during this study are included in this published article and its supplementary information files.

## Abbreviations

BAL: Bronchoalveolar lavage
CFU: Colony-forming unit
CXR: Chest X-ray
ETA: Endotracheal aspirate
ICU: Intensive care unit
LoS: Length of stay
MDR: Multi-drug resistant
MICU: Medical intensive care unit
MRSA: Methicillin-resistant *Staphylococcus aureus*
MSSA: Methicillin-sensitive *Staphylococcus aureus*
MV: Mechanical ventilation
NBAL: Non-bronchoscopic bronchoalveolar lavage
NRF: Normal respiratory flora
SA: *Staphylococcus aureus*
SICU: Surgical intensive care unit
SQ-ETA: Semi-quantitative endotracheal aspirate
VAP: Ventilator-associated pneumonia
VAT: Ventilator-associated tracheobronchitis
