# Supplementary material for: The utility of endotracheal aspirate bacteriology in identifying mechanically ventilated patients at risk for ventilator associated pneumonia: a single-center prospective observational study
Source: BMC Infect Dis. 2019 Aug 29;19:756. doi: 10.1186/s12879-019-4367-7 (PMC6716855; doi:10.1186/s12879-019-4367-7)
Supplement: Supplementary file 1 — Table S1. Antibiotic susceptibility of pathogenic bacterial species isolated in Lahey Clinic. Global antibiotic susceptibility rates are depicted for the bacterial species most commonly (≥2.5% of all ETA species) isolated from the ETA. Data was collected in the period between 01.01.2013 and 31.12.2013; only 1 isolate per patient and only the first isolate was considered. (DOCX 15 kb) [file 12879_2019_4367_MOESM1_ESM.docx]

**Table S1. Antibiotic susceptibility of pathogenic bacterial species isolated in Lahey Clinic.**

| **Organism** | **Antibiotic and % susceptibility of the isolates** | | | | | | | | | | | | | | | | | |
| --- | --- | --- | --- | --- | --- | --- | --- | --- | --- | --- | --- | --- | --- | --- | --- | --- | --- | --- |
|  | Oxacillin | Ampicillin | Ampicillin/ Sulbactam | Aztreonam | Cefazolin | Ceftriaxone | Cefepime | Meropenem | Trimethoprim/ Sulfamethoxazole | Ciprofloxacin | Levofloxacin | Gentamycin | Tobramycin | Amikacin | Clindamycin | Erythromycin | Tetracycline | Vancomycin |
| **Gram-positive organisms** | | | | | | | | | | | | | | | | | | |
| *Staphylococcus aureus* | 62 |  |  |  | 62 |  |  |  | 99 |  | 68 | 98 |  |  | 62 | 45 | 96 | 100 |
| **Gram-negative organisms** | | | | | | | | | | | | | | | | | | |
| *Klebsiella pneumoniae* |  |  | 84 | 96 | 91 | 97 | 97 | 99 | 91 | 95 | 96 | 97 | 96 | 98 |  |  | 86 |  |
| *Pseudomonas aeruginosa* |  |  |  | 80 |  |  | 96 | 85 |  | 75 | 70 | 87 | 93 | 96 |  |  |  |  |
| *Escherichia coli* |  | 60 | 63 | 94 | 78 | 95 | 95 | 99 | 78 | 81 | 82 | 93 | 92 | 99 |  |  | 77 |  |
| *Haemophilus influenzae* |  | 74 |  |  |  |  |  |  |  |  |  |  |  |  |  |  |  |  |
| *Enterobacter cloacae* |  |  |  | 84 |  | 78 | 92 | 98 | 85 | 89 | 95 | 92 | 93 | 100 |  |  | 84 |  |
| *Enterobacter aerogenes* |  |  |  | 93 |  | 91 | 99 | 100 | 99 | 97 | 97 | 100 | 100 | 99 |  |  | 98 |  |
| *Stenotrophomonas maltophilia* |  |  |  |  |  |  |  |  | 98 |  | 93 |  |  |  |  |  |  |  |

Global antibiotic susceptibility rates are depicted for the bacterial species most commonly (≥2.5% of all ETA species) isolated from the ETA.
Data was collected in the period between 01.01.2013 and 31.12.2013; only 1 isolate per patient and only the first isolate was considered.
