# Supplementary material for: The utility of endotracheal aspirate bacteriology in identifying mechanically ventilated patients at risk for ventilator associated pneumonia: a single-center prospective observational study
Source: BMC Infect Dis. 2019 Aug 29;19:756. doi: 10.1186/s12879-019-4367-7 (PMC6716855; doi:10.1186/s12879-019-4367-7)
Supplement: Supplementary file 2 — Table S2. Species isolated from ETA samples of patients with mixed Gram-positive and Gram-negative VAP episodes. (DOCX 13 kb) [file 12879_2019_4367_MOESM2_ESM.docx]

**Table S2. Species isolated from ETA samples of patients with mixed Gram-positive and Gram-negative VAP episodes.**

| **VAP case no.** | **Gram-positive component** | **Gram-negative component** |
| --- | --- | --- |
| 1. | *S. aureus* | *K. pneumoniae* |
| 2. |  | *K. pneumoniae, A. baumannii* |
| 3. |  | *E. coli* |
| 4. |  | *E. coli, K. oxytoca* |
| 5. |  | *P. aeruginosa* |
| 6. |  | *E. cloacae, H. influenza* |
| 7. |  | *E. aerogenes, S. marcescens* |
| 8. | Β-hemolytic group F Streptococci | *H. influenzae* |
| 9. | Diphteroid | *K. pneumoniae* |
