# Supplementary figures and images for: The utility of endotracheal aspirate bacteriology in identifying mechanically ventilated patients at risk for ventilator associated pneumonia: a single-center prospective observational study
Source: BMC Infect Dis. 2019 Aug 29;19:756. doi: 10.1186/s12879-019-4367-7 (PMC6716855; doi:10.1186/s12879-019-4367-7)

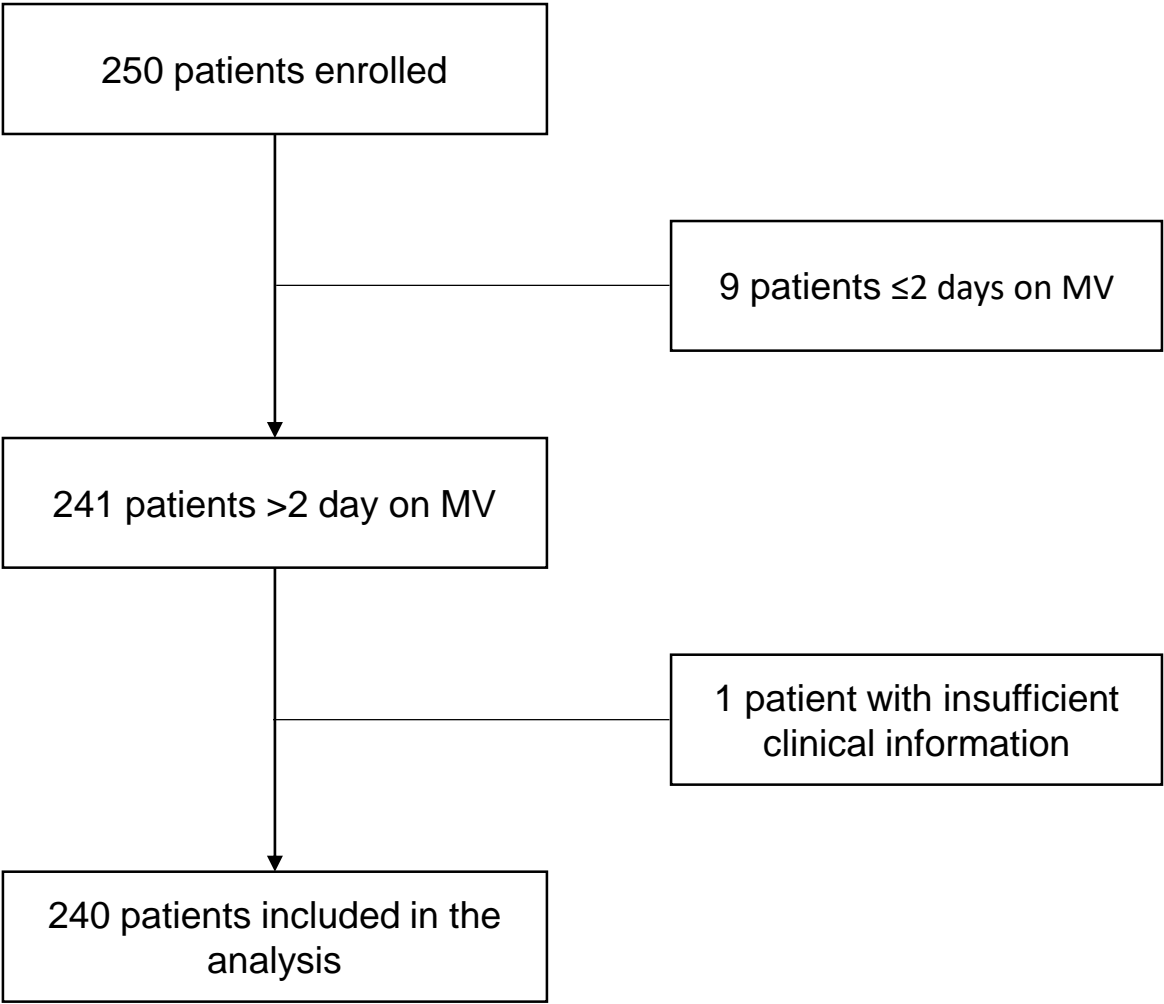

**Figure S1. Disposition of the patients included in the analysis.**

Supplement: Supplementary file 3 — Figure S1. Disposition of the patients included in the analysis. (PDF 181 kb) [file 12879_2019_4367_MOESM3_ESM.pdf]
