# Supplementary material for: The utility of endotracheal aspirate bacteriology in identifying mechanically ventilated patients at risk for ventilator associated pneumonia: a single-center prospective observational study
Source: BMC Infect Dis. 2019 Aug 29;19:756. doi: 10.1186/s12879-019-4367-7 (PMC6716855; doi:10.1186/s12879-019-4367-7)
Supplement: Supplementary file 4 — Figure S2. Patients with S. aureus monomicrobial VAP episodes. S. aureus burden dynamics (shown as SQ-ETA readout) and VAP clinical diagnosis days with VAP-relevant period highlighted. Only those days when ETA was obtained and analyzed are shown on X-axis. (PDF 80 kb) [file 12879_2019_4367_MOESM4_ESM.pdf]

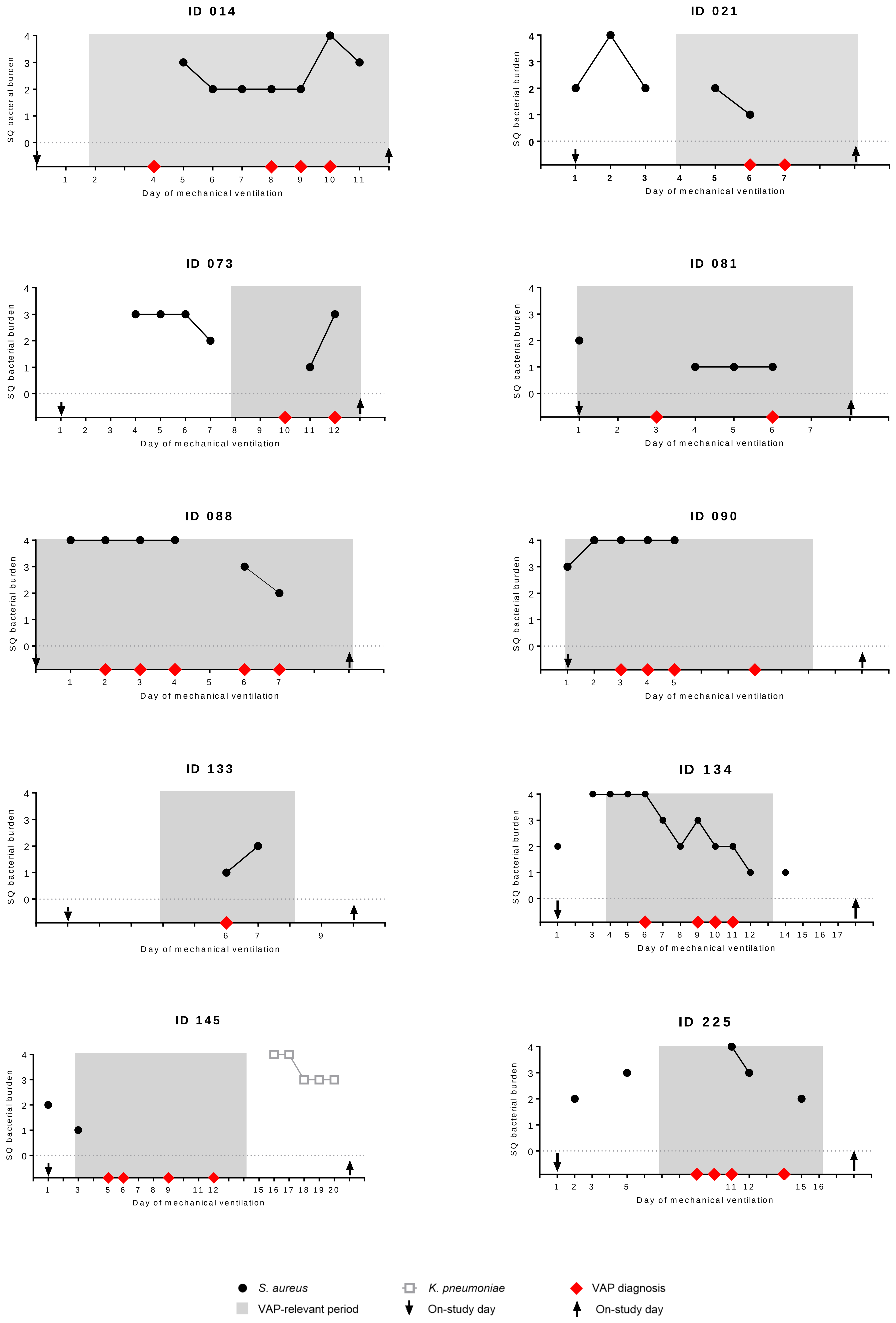


**Figure S2. Patients with *S. aureus* monomicrobial VAP episodes.** *S. aureus* burden dynamics (shown as SQ-ETA readout) and VAP clinical diagnosis days with VAP-relevant period highlighted. Only those days when ETA was obtained and analyzed are shown on X-axis.
