# Supplementary material for: The utility of endotracheal aspirate bacteriology in identifying mechanically ventilated patients at risk for ventilator associated pneumonia: a single-center prospective observational study
Source: BMC Infect Dis. 2019 Aug 29;19:756. doi: 10.1186/s12879-019-4367-7 (PMC6716855; doi:10.1186/s12879-019-4367-7)
Supplement: Supplementary file 5 — Figure S3. Patients with S. aureus mixed infection VAP episodes. S. aureus and other pathogens are indicated as SQ-ETA readout and VAP clinical diagnosis days with VAP-relevant period highlighted. Only those days when ETA was obtained and analyzed are shown on X-axis. (PDF 74 kb) [file 12879_2019_4367_MOESM5_ESM.pdf]

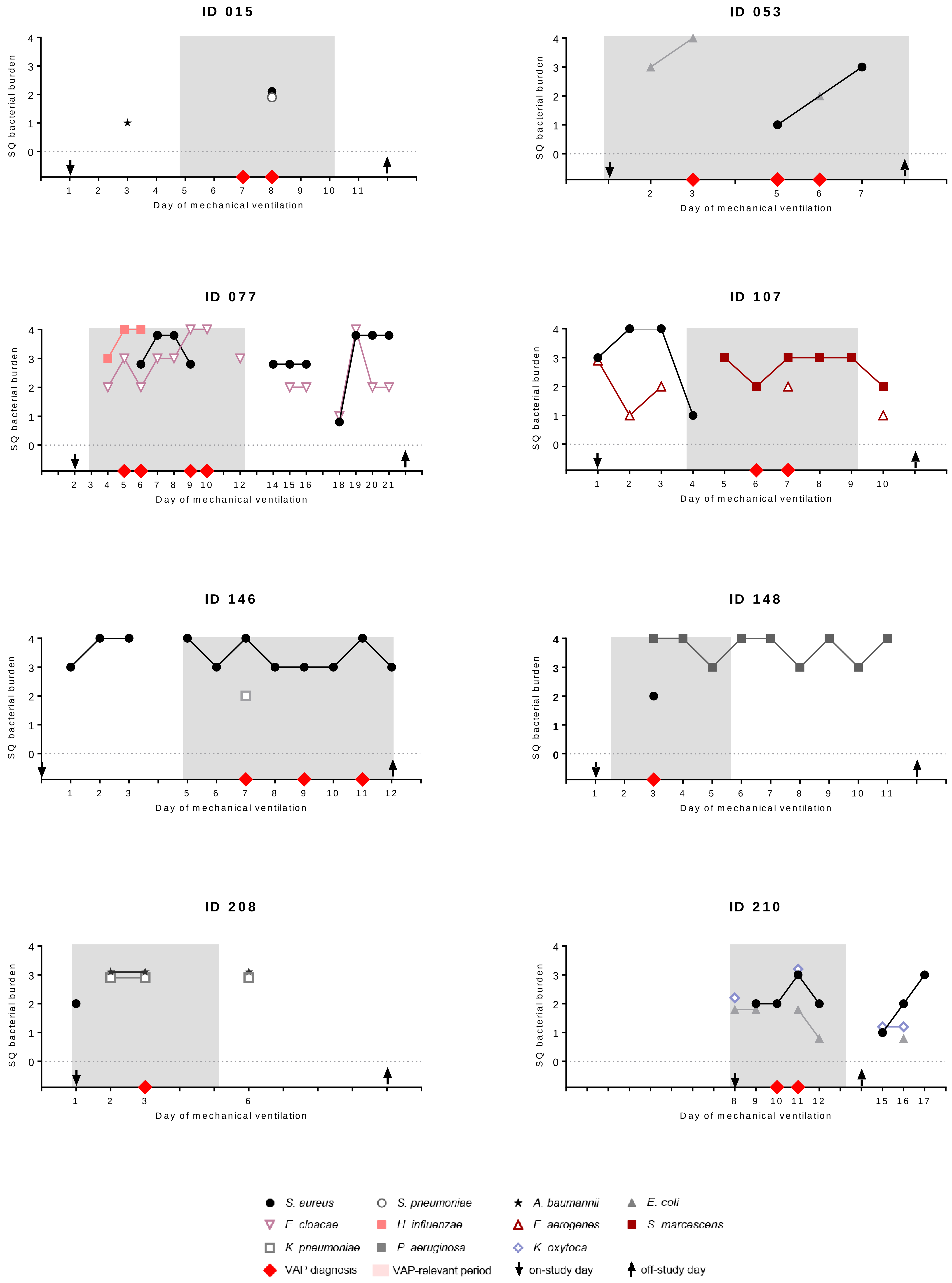

Figure S3. Patients with *S. aureus* mixed infection VAP episodes. *S. aureus* and other pathogens are indicated as SQ-ETA readout and VAP clinical diagnosis days with VAP-relevant period highlighted. Only those days when ETA was obtained and analyzed are shown on X-axis.
