# Supplementary material for: The utility of endotracheal aspirate bacteriology in identifying mechanically ventilated patients at risk for ventilator associated pneumonia: a single-center prospective observational study
Source: BMC Infect Dis. 2019 Aug 29;19:756. doi: 10.1186/s12879-019-4367-7 (PMC6716855; doi:10.1186/s12879-019-4367-7)
Supplement: Supplementary file 6 — Figure S4. Temporal distribution of VAP cases during the study period. A: Number of cases detected shown against corresponding time period. B: Number of cases normalized against number of recruited patients in the corresponding time period. Study commenced in the middle of June 2014; therefore VAP cases were counted at intervals from 17th of the month 16th of the subsequent month. In the December–January period no bacterial VAP cases were observed, despite active recruitment of patients in the study. (PDF 22 kb) [file 12879_2019_4367_MOESM6_ESM.pdf]

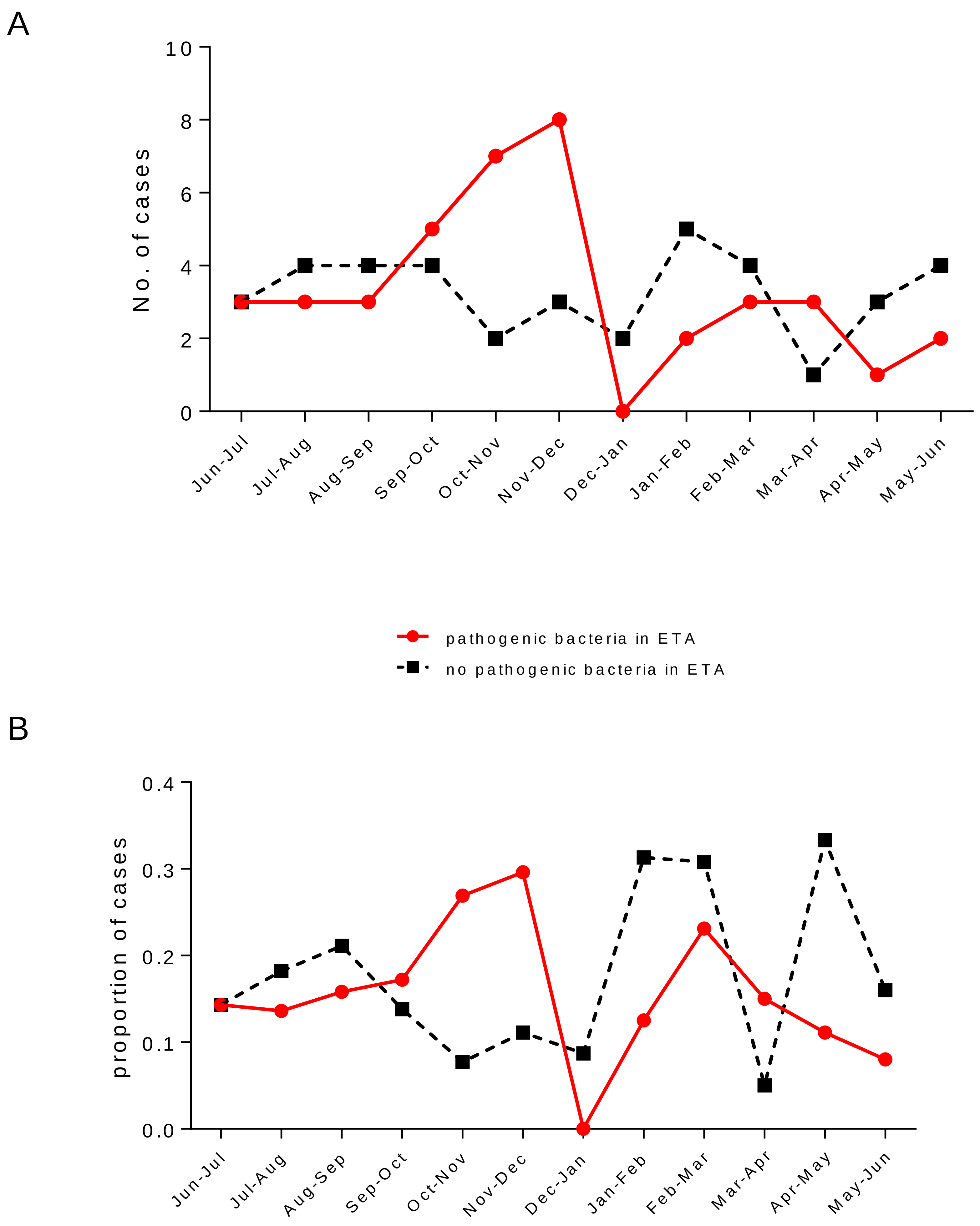

Figure S4. Temporal distribution of VAP cases during the study period. A: Number of cases detected shown against corresponding time period. **B:** Number of cases normalized against number of recruited patients in the corresponding time period. Study commenced in the middle of June 2014; therefore VAP cases were counted at intervals from 17th of the month 16th of the subsequent month. In the December-January period no bacterial VAP cases were observed, despite active recruitment of patients in the study.
