# Supplementary material for: The utility of endotracheal aspirate bacteriology in identifying mechanically ventilated patients at risk for ventilator associated pneumonia: a single-center prospective observational study
Source: BMC Infect Dis. 2019 Aug 29;19:756. doi: 10.1186/s12879-019-4367-7 (PMC6716855; doi:10.1186/s12879-019-4367-7)
Supplement: Supplementary file 7 — Demographic characteristic of the study population. (PDF 584 kb) [file 12879_2019_4367_MOESM7_ESM.pdf]

ID	ICU Location	On Study Date	Off Study Date	Age	Hosp Admit Date	ICU Admit Date	Ventilation Start Date	Hosp Admit Dx	PMH	Prior Hosp Admit	Nursing home resident	Medication on Admission	Medication last 90 days	ICU TX Disposition	Hosp DC Date	Hosp DC Disposition	Off Study Reason	ETOH	Current Smoker	Past Smoker	Emerg surgery	Trauma	Height inch	Weight lbs	BMI
		mddyy	mddyy	Range	mddyy									1 = tx to floor<br>2 = death<br>3 = tx to outside facility<br>4 = home<br>5 = other	mddyy	1 = tx to floor<br>2 = death<br>3 = tx to outside facility 4 = home<br>5 = other	1 = CMO<br>2 = Trach<br>3 = Death<br>4 = ICU D/C<br>5 = Extubation	0 = No<br>1 = Yes	0 = No 1 = Yes	0 = No 1 = Yes	0 = No<br>1 = Yes	0 = No 1 = Yes			
1	ICU	6/17/14	6/20/14	81 - 90	6/17/14	6/17/14	6/17/14	PNEUMONIA/SEPSIS	Hypertension<br>DM<br>Asthma<br>CHF<br>Dyslipidemia<br>Atrial Fibrilation<br>Gout	30 - 90 days	Never	Anticoagulants<br>Statins	Anticoagulants<br>Statins	FLOOR	6/25/14	Rehab	EXTUBATION	0	0	1	0	0	66	132	21.3
2	SICU	6/18/14	6/20/14	51 - 60	6/6/14	6/18/14	6/17/14	Fluid overload/worsening hepatic function/liver transplant	Cirrhosis<br>Hepatorenal syndrome<br>Leukemia<br>Esophageal varices<br>Benign colonic polyps<br>DM 2	30-90 days	Unknown	Insulin<br>Diuretics	insulin<br>Diuretics<br>Antibiotics	Floor	7/4/14	Home	Extubated	0	0	1	0	0	71	223.3	31.3
3	MICU	6/19/14	6/26/14	51 - 60	6/16/14	6/17/14	6/18/14	Pneumonia/Confusion and gait abnormalilty/Migraine	Colitis<br>COPD<br>Depression<br>Ulcerative Esophagitis<br>Restless leg syndrome	30 - 90 days	Never	Steroids<br>Immunosupressants	Immunosupressants	N/A	7/3/14	OUTSIDE FACILITY	EXTUBATION	1	0	1	0	0	63	208.3	36.9
4	SICU	6/19/14	6/20/14	61 - 70	6/18/14	6/18/14	6/19/14	Unruptured anterior communicating artery anuerysm/Post operative respiratory insuffiency	Hypertension<br>DM<br>Depression	Unknown	Never			FLOOR	6/23/14	HOME	EXTUBATION				1		64	340	58.3
5	SICU	6/20/14	7/2/14	81 - 90	6/19/14	6/19/14	6/19/14	Rectal perforation/fecal peritonitis/septic shock	Pneumonia<br>Anemia<br>Respiratory Failure<br>Dementia<br>Hypertension<br>Peri-splenic abscess s/p percutaneous drainage	30 - 90 days				OUTSIDE FACILITY	7/10/14	OUTSIDE FACILITY	ICU/DC				1		58	100	23.3
6	ICU	6/24/14	6/28/14	61 - 70	6/23/14	6/23/14	6/23/14	Hypoxic respiratory failure/Septic shock/Urosepsis/acute on chronic renal failure	Hypertension<br>Hyperlipidemia<br>Chronic kidney disease	> 1 year	Never	Statins	Statins	FLOOR	7/7/14	HOME	EXTUBATION	0	0	0	0	0	67	145	30.1
7	SICU	6/25/14	6/30/14	61 - 70	6/23/14	6/24/14	6/24/14	acute cholecystitis/Sepsis	Hyperlipidemia<br>Hypothyroidism<br>Diverticulitis s/p hepatico jejunostomy<br>CAD	30 - 90 days	Unknown			FLOOR	7/5/14	Home	EXTUBATION				1				
8	ICU	6/27/14	6/28/14	51 - 60	6/27/14	6/27/14	6/27/14	ACUTE REP FAILURE<br>METABOLIC ACIDOSIS<br>ACUTE RENAL FAILURE	Hypertension<br>DM<br>Hyperlipidemia<br>Depression<br>GI Bleed<br>Diverticulitis	> 1 year	Never			DECEASED	6/28/14	DECEASED	DECEASED	1	0	0	0	0	68	176.4	26.8
9	SICU	6/27/14	7/5/14	61 - 70	6/26/14	6/27/14	6/26/14	Abdominal aortic aneurysm/Gram negative pneumonia	Hypertension<br>COPD<br>OSA<br>DM<br>Atrial Fibrilation<br>Heart Failure<br>Gout	> 1 year				FLOOR	7/14/14	Rehab	EXTUBATED	0	1		1		70	259	37.6
10	MICU	6/30/14	7/14/14	71 - 80	6/29/14	6/29/14	6/29/14	Acute respiratory failure/Encephalopathy/Liver disease/MI/Sepsis and shock	DM<br>Partial thyroidectomy<br>ETOH abuse	Unknown	Never				7.21.14	DECEASED	EXTUBATED	1	0	0			70	208	29.9
11	ICU	7/2/14	7/11/14	51 - 60	7/1/14	7/1/14	7/1/14	Heart failure/respiratory failure/renal failure	Hypertension<br>DM 2<br>Hyperlipidemia<br>Gilbert disease	Last 30 days	Never	Statins		DECEASED	7/10/14	DECEASED	DECEASED	0	0	0	0	0	68	189	28.7
12	MICU	7/4/14	7/14/14	61 - 70	7/4/14	7/4/14	7/4/14	Hypoxic respiratory failure/pneumonia/Septic shock	Hyperlipidemia<br>DM<br>Pneumonia<br>Parkinsons<br>Myesthenia gravis	90 days to 1 year	Current	Insulin<br>Statins<br>Probiotics		FLOOR	7/19/14	Skilled Nursing Facility	Extubated						72	176.8	24
13	ICU	7/4/14	7/6/14	71 - 80	6/13/14	6/17/14	7/4/14	Hematuria/hemoptysis	Hypertension<br>Right sjogrens raynauds<br>GERD<br>Institial lung disease	30 - 90 days	Never	Probiotics<br>Statins		DECEASED	7/6/14	DECEASED	DECEASED	0	0	0	0	0	59	147	29.8
14	SICU	7/6/14	7/17/14	71 - 80	7/1/14	7/6/14	7/6/14	Ureteral blockage/hydronephrosis	Chronic kidney disease<br>COPD<br>Hypertension<br>Hypercholesterolemia	90 days to 1 year	Unknown	Statins	Insulin<br>Antibiotics<br>Anticoagulants	FLOOR	7/28/14	Long term care facility	EXTUBATED		0	0		1	69	209	31
15	SICU	7/7/14	7/17/14	41 - 50	7/6/14	7/6/14	7/6/14	Necrotizing fasciitis	CHF<br>Anxiety<br>Hypertension<br>Hyperlipidemia	Unknown	Never	Antibiotics		FLOOR	7/30/14	REHAB	EXTUBATED					1	74	220	28.2
16	MICU	7/11/14	7/15/14	51 - 60	7/9/14	7/11/14	7/11/14	Hypovolumic Shock	GERD	Unknown	Unknown			FLOOR	7/18/14	HOME	EXTUBATED	0	0	0	0	0	60	99	19.6
17	ICU	7/11/14	7/15/14	51 - 60	7/8/14	7/8/14	7/10/14	Thrombocytopenia/anemia	ITP<br>Atypical CLL<br>Hypertension<br>Hyperlipidemia<br>COPD<br>Hepatitis B<br>s/p spenectomy	Last 30 days	Never			Outside Hospital	7/14/14	Outside Hospital	Transfer to outside hospital	0	0	0	0	0	64	157	26.9
18	ICU	7/13/14	7/22/14	71 - 80	7/12/14	7/12/14	7/12/14	Cardiac arrest/respiratory failure	Anemia<br>Pulmonary embolism<br>Breast Cancer<br>CHF<br>Mitral valve regurgitation	Unknown	Never	Statins	Statins<br>Chemotherapeutics	DECEASED	7/22/14	DECEASED	CMO	0	0	1	0	0	61	132	24.9
19	SICU	7/14/14	7/25/14	51 - 60	7/12/14	7/13/14	7/13/14	Perforated gastric ulcer	COPD<br>CHF	Unknown	Unknown			Rehab	8/28/14	Rehab	TRACH	0	1	0	1	0	71	224	31.5
20	MICU	5/20/15	5/21/15	51 - 60	5/19/15	5/20/15	5/19/15	Unresponsiveness<br>Seizures	Unknown	Unknown	Unknown	Unknown		FLOOR	5/22/15	HOME	EXTUBATED	0	0	0	0	0	72	154	20.9
21	MICU	7/15/14	7/23/14	51 - 60	7/14/14	7/15/14	7/14/14	ETOH/respiratory failure/renal failure/Sepsis/Pneumonia	Alcohol dependence	Last 30 days	Never			FLOOR	7/29/14	HOME	EXTUBATED	1	1	0	0	0	69	154	22.7

22	SICU	7/15/14	7/19/14	81 - 90	7/7/14	7/15/14	7/15/14	Cholelithiasis/ cholecystitis secondary to pancreatic mass	DM<br>Gallstone/cholecystitis<br>GERD<br>Pancreatic cancer<br>Gilbert syndrome	Last 30 days	Unknown	Insulin	Antibiotics	DECEASED	7/19/14	DECEASED	CMO	0	0	0	1	0	59	119	24
23	ICU	7/20/14	8/1/14	61 - 70	7/19/14	7/19/14	7/19/14	Multisystem failure/Septic shock/MSSA pneumonia	CAD<br>Hypertension<br>Hyperlipidemia<br>COPD<br>Alcohol and tobacco dependence	> 1 year	Never	Statins	Statins	DECEASED	8/1/14	DECEASED	DECEASED				0	0	71	174	24.4
24	MICU	7/20/14	7/26/14	51 - 60	7/11/14	7/20/14	7/20/14	Liver cirrohsis/ARF/Hepatorenal syndrome	Atrial Fibrilation<br>CHF<br>Cirrhosis	Last 30 days	Unknown	Statins	Antibiotics<br>Statins	FLOOR	8/5/14	DECEASED	EXTUBATED				0	0	60	101.9	20
25	MICU	5/20/15	5/23/15	71 - 80	5/18/15	5/18/15	5/18/15	Subdural Hematoma	HTN<br>DVT<br>Kidney Transplant<br>Aortic Stenosis<br>Focal Sclerosing GN<br>Cholecystistis<br>Pnemonia<br>PE<br>Angina<br>Diastolic CHF<br>Skin Cancer<br>CKD stage 3<br>High Cholesterol	Last 30 days	Unknown	Albeutrol<br>Steriod<br>Anti-Coaugulation		Deceased	5/22/15	Deceased	Deceased	0	0	0	0	0	70	208	29.8
26	ICU	7/22/14	7/25/14	71 - 80	7/21/14	7/21/14	7/21/14	Acute respiratory failure/Septic shock/UTI/Pneumonia	Atrial Fibrilation<br>Cirrhosis<br>DM 2<br>Hypothyroidism<br>Cancer<br>Gastric Ulcer	> 1 year	Never	Statins	Antibiotics	FLOOR	8/2/14	Long Term Care Facility	EXTUBATED	0	0	0	0	0	62	211.2	38.9
27	MICU	7/22/14	7/24/14	71 - 80	7/21/14	7/22/14	7/21/14	Seizures/ARF/Pleural effusion/Shock	Pneumonia<br>C. Difficile infection<br>Hypertension<br>Hyperlipidemia<br>Chronic kidney disease<br>GERD	Last 30 days	Never	Antibiotics<br>Statins	Antibiotics<br>Statins	FLOOR	8/1/14	HOME	EXTUBATED	0	0	1	0	0	60	121.3	23.7
28	MICU	7/22/14	7/23/14	61 - 70	7/15/14	7/21/14	7/21/14	Septic shock secondary to pneumonia	CHF<br>Pneumonia<br>Hypertension<br>COPD	> 1 year	Never	Statins	Antibiotics<br>Immunosupressants	DECEASED	7/30/14	DECEASED	EXTUBATED	0	0	1	0	0	72	309.3	42.4
29	SICU	7/22/14	7/30/14	51 - 60	7/21/14	7/21/14	7/21/14	Polytrauma		Unknown	Never			TRANSFER	8/1/14	OUTSIDE HOSP	EXTUBATED	0	0	0	1	1	62	169.8	31.1
30	ICU	7/25/14	7/27/14	41 - 50	7/19/14	7/19/14	7/19/14	Varicella infection/ARDS	Fibromyalgia<br>Anemia<br>COPD<br>Sarcodosis<br>Brain aneurysm s/p clipping	Last 30 days	Never	Immunosupressants<br>Steroids	Antibiotics	FLOOR	7/30/14	REHAB	EXTUBATED	0	1	0	0	0	64	185.7	31.8
31	MICU	7/25/14	8/2/14	71 - 80	7/24/14	7/24/14	7/24/14	Acute respiratory failure/cardiogenic shock	Hypertension<br>Hyperlipidemia<br>Pneumonia<br>CHF<br>Osteoarthritis	Last 30 days	Unknown	Statins	Antibiotics	FLOOR	8/9/14	REHAB	EXTUBATED	0	0	0	0	0	59	156	31.4
32	ICU	7/26/14	7/29/14	61 - 70	7/25/14	7/25/14	7/25/14	Acute respiratory failure/shock	Pulmonary embolism<br>Thyroid cancer<br>Cardiomyopathy<br>basal cell carcinoma<br>Metastatic breast cancer	Unknown	Never	Anticoagulants<br>Chemotherapeutics	Anticoagulants<br>Chemotherapeutics	FLOOR	8/15/14	DECEASED	EXTUBATED	0	0	0	0	0	64	110	18.8
33	SICU	7/31/14	8/3/14	51 - 60	7/26/14	7/30/14	7/30/14	Respiratory failure/Sepsis/Renal failure	Hypertension<br>Cirrhosis<br>Depression<br>GERD	Last 30 days	Current			DECEASED	8/3/14	DECEASED	CMO	1	0	1	0	0	67	235	37
34	SICU	7/31/14	8/6/14	81 - 90	7/30/14	7/30/14	7/30/14	Peforated colon	S/post Pacemaker<br>Hypothyroidism<br>B-cell lymphoma<br>Melanoma	Last 30 days	Never	Chemotherapeutics	Antibiotics	FLOOR	8/13/14	DECEASED	EXTUBATED	0	0	0	1	0	64	161	27.5
35	MICU	7/31/14	8/3/14	> 91	7/31/14	7/31/14	7/31/14	Pneumonia/Sepsis	CAD<br>CHF<br>Atrial Fibrilation<br>Pulmonary embolism<br>Hypertension<br>Dementia<br>BPH<br>Colon adenoma	Unknown	Unknown	Statins<br>Anticoagulants	Statins<br>Anticoagulants	FLOOR	8/9/14	REHAB	EXTUBATED	0	0	1	0	0	69	198	29.2
36	MICU	8/3/14	8/12/14	71 - 80	8/2/14	8/2/14	8/2/14	Acute hypoxic respiratory failure/E.coli septicemia	CAD<br>Atrial Fibrilation<br>Hyperlipidemia<br>CHF<br>Pneumonia<br>Hypothyroidism<br>oligodendroglioma	> 1 year	Unknown			FLOOR	8/22/14	SKILLED NURSING FACILITY	EXTUBATED	0	0	0	0	0	69	158.4	23.5
37	SICU	8/7/14	8/25/14	61 - 70	8/6/14	8/6/14	8/6/14	Necrotizing fasciitis/Septic shock	Hypertension<br>Atrial Fibrilation<br>Gout	Unknown	Unknown			Long term care facility	9/25/14	Long term care facility	Tracheostomy				1	0	69	231	34.3
38	MICU	8/10/14	8/16/14	61 - 70	8/9/14	8/9/14	8/9/14	Acute respiratory failure/Septic shock	Crest syndrome<br>Esophageal strictures<br>Breast cancer s/p masectomy	Unknown	Never			Floor	8/21/14	Rehab	Extubated	0	0	0	0	0	63	123	21.9
39	MICU	8/10/14	8/17/14	61 - 70	8/9/14	8/9/14	8/10/14	Encephalopathy/GI bleed/Septic shock	Hypertension<br>Cirrhosis<br>Depression<br>Chronic kidney disease	Never	Never			Deceased	8/16/14	Deceased	CMO	0	0	0	0	0	63	156	27.7
40	SICU	8/11/14	8/19/14	81 - 90	8/9/14	8/9/14	8/9/14	Small bowel obstruction	Hyperlipidemia<br>Prostate cancer<br>Hypertension<br>H.Pylori<br>Colon polyp	> 1 year	Unknown	Statins		Floor	8/29/14	Skilled Nursing Facility	Extubated	0	0	0	1	0	69	169.4	25.1
41	SICU	8/11/14	8/21/14	61 - 70	8/9/14	8/9/14	8/11/14	Polytrauma	CAD<br>Hypertension<br>DM<br>S/p CABG<br>Gout	Unknown	Unknown	Statins		Long term care facility	8/28/14	Long term care facility	Tracheostomy	1	0	0	0	1	69	259.6	38.5
42	SICU/MICU	8/12/14	8/23/14	51 - 60	8/9/14	8/9/14	8/10/14	Subdural hematoma/Acute respiratory failure/GNR pneumonia	COPD	Unknown	Unknown	Anticoagulants		Floor	8/27/14	Deceased	Extubated	0	1	0	0	0	63	173.1	30.7

									Hepatocellular Carcinoma<br>Cirrhosis<br>Hypothyroidism<br>Rheumatologic disorder<br>Hypercoagulable state<br>DVT<br>Hyperlipidemia<br>Colangio carcinoma																
43	SICU	8/14/14	8/16/14	51 - 60	8/14/14	8/14/14	8/14/14	Dislodged IR drain/ Septic shock/Cardiogenic shock/Multiple organ failure	COPD<br>Hypothyroidism<br>Hepatocellular Carcinoma<br>Hepatitis C<br>GERD<br>s/p live donor liver transplant 2009	Last 30 days	Unknown	Antibiotics	Antibiotics<br>Immunosupressants	Deceased	8/15/14	Deceased	CMO	1	0	1	0	0	65	139	23.2
44	MICU	8/14/14	8/15/14	41 - 50	8/14/14	8/14/14	8/14/14	Seizure like activity/ severe headaches/Cryptococcal meningitis/Respiratory failure	Hypothyroidism<br>Fibromyalgia<br>Hypopituitarism s/p pitiutary tumor resection	Unknown	Unknown	Anticoagulants	Antibiotics<br>Immunosupressants	Deceased	8/15/14	Deceased	CMO	0	0	0	0	0	62	125	23.1
45	ICU	8/16/14	9/4/14	51 - 60	8/14/14	8/15/14	8/15/14	RLL mass vs. pneumonia	Hypercholesterolemia<br>Paranoid schizophrenia	Unknown	Current	Statins	Antibiotics	Long term care facility	9/19/14	Long term care facility	Tracheostomy	0	1	0	0	0	69	152	22.5
46	SICU	5/21/15	5/23/15	71 - 80	5/3/15	5/11/15	5/21/15	Atherosclerosis of Arteries of Extremities with Gangrene	PVD<br>CAD<br>DM<br>HTN<br>Hyperlipdimia<br>Venous insufficiency	Unknown	Never	Insulin<br>Statin		Skilled nursing Facility	6/22/15	Skilled nursing Facility	Extubated	0	1	0	0	0	65	216	35.9
47	MICU	8/18/14	8/19/14	71 - 80	8/15/14	8/16/14	8/17/14	Acute respiratory failure	Hypertension<br>Asthma<br>Dyslipidemia	Unknown	Never	Statins	Antibiotics	Floor	8/26/14	Home w/ VNA	Extubated	0	0	1	0	0	73	171	22.7
48	MICU	8/20/14	8/26/14	31 - 40	8/16/14	8/16/14	8/18/14	Respiratory failure/Pneumonia/Septic shock/Acute renal failure	Miscarriage	30 - 90 days	Never	Antibiotics	Antibiotics	Deceased	8/26/14	Deceased	Deceased				0	0	62	178	32.9
49	ICU	8/21/14	8/25/14	31 - 40	8/17/14	8/17/14	8/18/14	Hepatic failure/Sepsis/Severe shock/multiorgan failure	Bipolar Depression<br>Anxiety	Unknown	Never	Statins	Antibiotics	Deceased	8/25/24	Deceased	CMO	0	0	0	0	0	64	183	31.2
50	SICU	8/22/14	9/3/14	71 - 80	8/21/14	8/22/14	8/22/14	Postoperative sepsis/gastric leak	COPD<br>Hypertension<br>Hypothyroidism	Unknown	Unknown	Statins<br>Antibiotics	Antibiotics	Long term care facility	10/7/14	Long term care facility	Tracheostomy	0	0	1	0	0	67	284	44.7
51	MICU	8/26/14	8/29/14	71 - 80	8/26/14	8/26/14	8/26/14	Acute respiratory failure/Septic shock/CHF	DM<br>Hypertension<br>Recurrent UTI<br>Atrial Fibrilation<br>Hypothyroidism<br>Anemia<br>Osteoarthritis<br>CAD	90 days to 1 year	Never	Statins<br>Anticoagulants<br>Antibiotics	Antibiotics	Floor	9/4/14	REHAB	Extubated	0	0	1	0	0	60	136	26.7
52	MICU	8/26/14	9/2/14	61 - 70	8/25/14	8/25/14	8/25/14	Acute hypoxic respiratory failure/Pneumonia	Bladder Cancer<br>Gastric ulcers	90 days to 1 year	Unknown	Antibiotics		FLOOR	9/8/14	Home	Extubated	0	1	0	0	0	69	220	32.7
53	MICU	8/27/14	9/3/14	51 - 60	8/26/14	8/26/14	8/26/14	Lower extremity paralysis/ epidural/spinal abscess/MRSA bacteremia	Depression<br>Hep C Cirrhosis	Unknown	Current		Antibiotics	Deceased	9/3/14	Deceased	CMO	1	0	0	0	0	73	213	28.2
54	SICU	8/27/14	8/30/14	51 - 60	8/26/14	8/26/14	8/26/14	s/p right partial nephrectomy	Hypertension<br>Osteoarthritis<br>Renal Cancer	Unknown	Unknown	Statins	Antibiotics	Floor	9/2/14	Home	Extubated	0	0	1	0	0	71	251	35.2
55	SICU	8/29/14	8/31/14	71 - 80	8/27/14	8/27/14	8/28/14	PEA Arrest	Cardiomyopathy<br>Anemia<br>Malignant colon polyp<br>Tachyarrythmia<br>H.pylori	Last 30 days	Unknown		Antibiotics	Floor	9/16/14	REHAB	Extubated	0	0	0	0	0	63	99	17.5
56	SICU	8/30/14	9/5/14	81 - 90	8/29/14	8/29/14	8/28/14	Right hepatic artery transection s/p laparoscopic cholecystectomy	Atrial Fibrilation<br>CHF<br>Lymphoma<br>Hyperlipidemia<br>Hypertension<br>Hypothyroidism	Unknown	Unknown	Antibiotics	Antibiotics	Floor	9/23/14	Nursing Facility	EXTUBATED	0	0	0	1	0	66	110	17.7
57	MICU	9/2/14	9/4/14	71 - 80	9/1/14	9/1/14	9/1/14	Altered mental status/Right sided weakness/Unresponsive /Viral meningitis/ARF	Atrial Fibrilation<br>Stroke<br>Hypertension<br>Hyperlipidemia<br>Breast cancer s/p right masectomy	> 1 year	Never	Statins		Floor	9/9/14	Rehab	Extubated	0	0	0	0	0	65	145	24.2
58	SICU	9/2/14	9/16/14	61 - 70	8/30/14	9/2/14	9/2/14	Polytrauma following a fall	ETOH abuse<br>Hypertension<br>C2 fracture<br>Inguinal hernia repair	>1 year	Never			Long term care facility	9/26/14	Long term care facility	Extubated	1	0	0	0	0	73	169	22.5
59	MICU	9/3/14	9/18/14	51 - 60	9/2/14	9/2/14	9/2/14	Acute hypoxic respiratory failure/Right sided opacification	Hypertension<br>Alcohol/tobacco use	Unknown	Unknown	None	Antibiotics	Long term care facility	10/1/14	Long term care facility	Tracheostomy	1	1	0	0	0	72	165	22.4
60	ICU	9/4/14	9/5/14	41 - 50	9/4/14	9/4/14	9/4/14	Altered mental status	Crohn's with ileocolic resection<br>Obstructive ureterolithiasis<br>s/p right nephrectomy	30-90 days	Never	Pain medication		Floor	9/7/14	Home	Extubated	0	1	0	0	0	65	121	20.1
61	SICU	9/5/14	9/8/14	81 - 90	9/4/14	9/4/14	9/4/14	Perforated bowel	Aortic stenosis<br>Breast cancer s/p lumpectomy<br>Hypertension<br>Hypercholesterolemia	Unknown	Uknown	Statins<br>Pain medication		Floor	9/18/14	Long term care facility	Extubated	0	0	0	1	0	62	173	32
62	SICU	9/9/14	9/18/14	61 - 70	9/8/14	9/8/14	9/8/14	Enterocutaneous fistula	Cholectomy	Unknown	Unknown	Pain medication<br>Statins<br>Anticoagulant	Antibiotics	Deceased	9/17/14	Deceased	CMO		0	0	0	0	61	160	30.4
63	MICU	5/21/15	6/4/15	61 - 70	5/19/15	5/20/15	5/20/15	Alcoholic Ketoacidosis	Respiratory Failure<br>DVT<br>Alcoholic Hepatitis<br>SVT	90 days to 1 year	No	Albuterol<br>Metoprolol<br>Steriod<br>CYP17 inhibitor (Abiraterone		Long Term Care	6/17/15	Long term Care	Transfer to another	1	1	0	0	0	69	170	25.1

									Metastatic CA Prostate<br>Cardiomyopathy<br>PEA																
64	MICU	9/11/14	9/25/14	61 - 70	9/10/14	9/10/14	9/10/14	Septic shock/C.Diff colitis/Coagulopathy	Cirrhosis<br>s/p liver transplant<br>s/p kidney transplant<br>IDDM 2<br>Anemia<br>VRE endocarditis<br>Hypertension<br>Gout	30-90 days	Never	Immunosuppresants<br>Pain medication	Antibiotics<br>Immunosuppresants	Long term care facility	10/8/14	Long term care facility	Tracheostomy	0	0	1	0	0	70	180	25.9
65	SICU	9/11/15	9/15/14	81 - 90	9/10/14	9/10/14	9/10/14	Found unrepsonsive/ seizure	Hypertension<br>Hyperlipidemia<br>Fibromyalgia<br>GERD<br>Angina pectoris<br>Dementia<br>Essential tremor<br>CKD stage III<br>Left ventricular hypertrophy<br>Ovarian cancer<br>Aortic stenosis	Unknown	Current	Corticosteroids<br>Anticoagulant		Deceased	9/16/14	Deceased	Extubated	0	0	0	1	0	66	179.5	28.9
66	SICU	9/13/14	9/17/14	51 - 60	9/11/14	9/13/14	9/13/14	Wound Dehiscence	Metastatic rectal cancer<br>Hypertension<br>s/p laparoscopic cholecystectomy<br>Hyperlipidemia<br>CAD, s/p MI, s/p stent<br>COPD	30-90 days	Never	Antibiotics<br>Pain Medication	Antibiotics	Floor	9/22/14	Home with VNA	Extubated	0	1	0	1	0	69	189.2	28.1
67	SICU	9/19/14	10/3/14	71 - 80	9/18/14	9/18/14	9/18/14	Acute right subdural hemorrhage	Stroke<br>End stage renal disease<br>Dementia	Unknown	Unknown		Heparin drip	Rehab	10/2/14	Rehab	Tracheostomy				1		66	97	15.7
68	SICU	9/19/14	9/21/14	71 - 80	9/19/14	9/19/14	9/19/14	Expanding abdominal aortic aneurysm with evidence of type 2 endoleak	Hypertension<br>Hypercholesterolemia<br>Chronic lymphoid leukemia<br>s/p appendectomy<br>s/p hemorrhoidectomy<br>Polymyalgia rheumatica	1 year	Never	Statins<br>Steroids		Floor	9/29/14	Home	Extubated				1		70	198	28.4
69	ICU	9/20/14	9/23/14	51 - 60	9/18/14	9/19/14	9/19/14	Pneumonia/fevers	Myotonic dystrophy<br>Closed fracture of the cervical vertebral body, dysphagia, s/p G tube placement<br>Aspiration pneumonia	1 year	Unknown	Vasopressors	Antibiotics	Deceased	10/2/14	Deceased	CMO	0	0	0	0	0	73	143	19
70	MICU	9/21/14	9/23/14	61 - 70	9/20/14	9/20/14	9/20/14	Acute respiratory failure/presumed seizure	Depression/anxiety<br>Chronic left hip wound<br>Hypertension<br>Narcotic dependence	Unknown	Never	Diuretic<br>Antibiotics		Floor	9/26/14	Rehab	Extubated	0	1	0	0	0	66	101.9	16.4
71	SICU	9/22/14	9/26/14	61 - 70	9/17/14	9/21/14	9/21/14	Elective robotic-assisted sigmoid colectomy	Diverticulitis<br>Community aquired pneumonia<br>Shingles<br>Nasal hemorrhage<br>Pancreatic cyst<br>Ovarian cyst<br>Vertigo	30-90 days	Unknown	Antibiotics<br>Probiotcs	Antibiotics<br>Immunosuppressants	Deceased	9/26/14	Deceased	CMO				1		62	116.8	21.5
72	ICU	5/21/15	5/23/15	31 - 40	5/20/15	5/20/15	5/20/15	Hyponatremia	Cerebral Palsy<br>Schizo affective<br>Schizophrenia<br>Depression , Anxiety<br>Left eye blindness secondary to glaucoma<br>GERD<br>Narcotic abuse	Unknown		Unknown		Floor	6/1/15	Home with Health Care service	Extubated	0	0	0	0	0	61	119	22.5
73	SICU	9/25/14	10/7/14	71 - 80	9/24/14	9/24/14	9/24/14	Abdominal aortic aneurysm	CAD s/p CABG<br>Afib<br>Hypertension	Unknown	Unknown			Long term care facility	10/20/14	Long term care facility	Extubated				1		64	193.6	33.1
74	SICU	9/25/14	9/27/14	61 - 70	9/22/14	9/23/14	9/25/14	Abdominal aortic aneurysm	COPD<br>GERD<br>Hypertension	>1 year	Never			Floor	10/3/14	Rehab	Extubated	1	0	1	1	0	67	145	22.8
75	SICU	9/26/14	9/27/14	41 - 50	9/20/14	9/24/14	9/24/14	Small bowel obstruction secondary to umbilical hernia	Perforated mucinous adenocarcinoma, s/p appendectomy<br>s/p laparoscopic right hemicolectomy<br>s/p hot intraperitoneal chemotherapy<br>Small bowel obstruction	30-90 days	Never	None	Antibiotics	Floor	10/7/14	Home	Extubated	0	0	0	1	0	70	229.5	32.9
76	MICU	9/27/14	10/5/14	81 - 90	9/27/14	9/27/14	9/27/14	PEA arrest/acute respiratory failure	Diabetes<br>Prostate enlargement<br>Cervical bone spur s/p surgery<br>Dysphagia	Unknown	Never	Hypoglycemics		Deceased	10/4/14	Deceased	CMO	0	0	1	0	0	64	100.1	17.1
77	SICU	5/23/15	6/12/15	71 - 80	5/21/15	5/22/15	5/21/15	Primary Adenocarcinoma of Pancreas	Pancreatic CA<br>HTN<br>Hyperlipidemia<br>CKD<br>Peripheral Neuropathy<br>DM,<br>GOUT<br>BPH	Unknown		Statins		Deceased	6/20/15	Deceased	Tracheostomy	0	0	1	0	0	67	194	30.4
78	ICU	9/27/14	9/29/14	31 - 40	9/27/14	9/27/14	9/27/14	Altered mental status/medication overdose	Nonischemic cardiomyopathy<br>ICD placement in 2009<br>Hyperlipidemia<br>Obesity<br>OSA<br>Depression	30-90 days	Never	Statins		Floor	10/4/14	Home	Extubated	0	0	0	0	0	70	220	31.6
79	ICU	9/27/14	9/28/14	51 - 60	8/6/14	9/25/14	9/25/14	Exploratory laparotomy of extensive mass	Chronic kidney disease<br>Diabetes, complicated by retinopathy, neuropathy, nephropathy<br>Hyperlipidemia<br>s/p adrenalectomy	Last 30 days	Unknown	Hypotensives	Antibiotics<br>Immunosuppresants<br>Pressors	Deceased	9/28/14	Deceased	CMO	1	0	0	1	0	65	217.8	36.4

									Right adrenal tumor																
80	SICU	9/28/14	9/30/14	81 - 90	9/26/14	9/27/14	9/27/14	Left temporal hemmorhage	COPD<br>AAA<br>Diabetes<br>GI bleed<br>TIA<br>Hypertension<br>CAD<br>Alzheimer dementia	30-90 days	Unknown	Anticoagulants<br>Statins		Deceased	9/30/14	Deceased	CMO						73	196.2	25.9
81	SICU	9/30/14	10/7/14	61 - 70	9/17/14	9/29/14	9/29/14	Increased output from abscess drain	Percolonic abscess<br><br>Complicated diverticulitis<br>Seizure disorder<br>Atypical meningioma<br>MDD<br>Hypothyroidism<br>Diabetes	Last 30 days	Current	Antibiotics	Antibiotics<br>Immunosuppressants	Floor	12/20/14	Rehab	Extubated	0	0	1	1	0	60	159.9	31.5
82	MICU	10/1/14	10/4/14	18 - 30	9/30/14	9/30/14	9/30/14	Presumed heroin overdose/acute respiratory failure/coma	Depression<br>Drug use<br>Hepatitis C<br>Question endocarditis in 2012	Unknown	Never	Unknown		Deceased	10/3/14	Deceased	CMO	0	1	0	0	0	N/A	194	N/A
83	SICU	10/3/14	10/4/14	61 - 70	10/1/14	10/1/14	10/1/14	Peripheral aterial occulsive disease	CAD s/p MI<br>Hypertension<br>Lumbar radiculopathy	>1 year	Unknown			Floor	10/9/14	Rehab	Extubated		1		1		72	176.4	23.9
84	SICU	10/3/14	10/10/14	31 - 40	10/3/14	10/3/14	10/3/14	Acute respiratory failure/sepsis	Depression<br>GERD<br>Obesity	Unknown	Never	Antibiotics<br>Pressors<br>Sedatives		Floor	10/29/14	Rehab	Extubated	0	0	0	1	0	64	382.8	65.5
85	SICU	10/4/14	10/8/14	61 - 70	10/3/14	10/3/14	10/3/14	Right lower extremity pain	CAD<br>Cervical disk disorder with myelopathy<br>MI in 1989 and 2009<br>Lumbar laminectomy<br>Hyperlipidemia<br>Hypertension<br>COPD	Last 30 days	Unknown			Floor	10/25/14	Rehab	Extubated	0	0	1	1	0	63	121	21.5
86	ICU	10/5/14	10/8/14	51 - 60	10/4/14	10/4/14	10/4/14	Acute respiratory failure/Hypoxic brain injury/Septic shock	Type II diabetes<br>Hypertension<br>Stage IV renal disease	Unknown	Never	Statins	Antibiotics	Deceased	10/8/14	Deceased	CMO				0	0	63	140.8	25
87	SICU	10/8/14	10/10/14	81 - 90	9/20/14	10/8/14	10/8/14	Urosepsis/bilteral inguinal hernia/small bowel obstruction	Bladder outlet obstruction<br>Afib<br>Basal cell carcinoma w/ actinic keratosis<br>Prostate cancer<br>Cervical radiculopathy<br>Heart block<br>Hypertension<br><br>Lumbar disk degeneration	90 days-1 year	Never	Statins<br>Steroids	Antibiotics	Floor	10/16/14	Care facility	Extubated	0	0	0	1	1	70	176	25.2
88	ICU	10/8/14	10/17/14	51 - 60	10/8/14	10/8/14	10/8/14	Altered mental status/ride sided facial droop	None known	Unknown	Never	None			11/8/14	Deceased	Tracheostomy	0	0	0	0	0	72	314.6	42.7
89	SICU	10/9/14	10/23/14	81 - 90	10/9/14	10/9/14	10/8/14	Motor vehicle accident/subdural hematoma	Arthritis<br>Afib<br>Parkinsons	Unknown	Never	Statins<br>Anticoagulants		Floor	10/31/14	Hospice	Extubated				1	1	62	148.1	27.3
90	SICU	10/10/14	10/21/14	18 - 30	10/9/14	10/9/14	10/9/14	Motor vehicle accident	Hepatitis C<br>Heroin addiction<br>Depression/anxiety	Unknown	Current	Unknown		Floor	11/13/14	Home	Extubated				0	1	64	154	26.3
91	SICU	10/10/14	10/12/14	71 - 80	10/9/14	10/9/14	10/9/14	Large right occipital intracerebral hemmorrhage	GI bleed<br>Osteoarthritis<br>Pseudogout<br>Mirgraines	>1 year	Unknown	Unknown	Antibiotics	Deceased	10/13/14	Deceased	CMO	0	0	0	1	0	66	165	26.6
92	SICU	10/10/14	10/11/14	41 - 50	8/26/14	10/10/14	10/10/14	Fournier gangrene	Type II diabetes<br>Recurrent scrotal infections<br><br>Lower extremity cellulitus and MRSA infection	Unknown	Unknown	Unknown	Antibiotics	Floor	10/18/14	Rehab	Self-Extubated				1		72	371.8	50.5
93	SICU	10/10/14	10/25/14	81 - 90	10/10/14	10/10/14	10/10/14	Mechanical fall/multiple fractures		>1 year					11/10/14	Rehab	Extubated						71	220	30.5
94	SICU	10/12/14	10/13/14	51 - 60	10/11/14	10/11/14	10/11/14	Colon perforation s/p colonoscopy	Metastatic uterine cancer<br>Radiation colitis<br><br>Irritable bowel syndrom<br>Hyperlipidemia	Last 30 days	Unknown	Hypotensives		Floor	10/26/14	Home	Extubated	0	0	0	1	0	64	199.5	34.1
95	MICU	10/13/14	10/15/14	51 - 60	10/12/14	10/12/14	10/12/14	Acute respiratory failure/septic shock/bacteremia	Diabetes<br>End stage renal disease<br>Hypertension<br>Hyperlipidemia<br><br>Peripheral vascular disease<br>CAD s/p CABG<br>CHF	90 days-1 year	Never	Antibiotics<br>Pressors		Floor	10/24/14	Home w/ VNA	Extubated	0	0	0	0	0	69	232.3	34.5
96	SICU	10/16/14	10/29/14	61 - 70	9/26/14	10/11/14	10/14/14	Perihepatic/peribiliary abscesses	Chronic pain syndrome<br>Hyperlipidemia<br>Trigeminal neurlgia<br>Hypertension<br>s/p laminectomy<br>s/p mitral valve replacement	30-90 days	Never	Anticoagulants<br>Pain meds	Antibiotics	Floor	11/17/14	Rehab	Extubated	0	0	1	1	0	69	224.4	33.3
97	MICU	10/16/14	10/22/14	41 - 50	10/3/14	10/15/14	10/15/14	Encephalopathy/increasing edema	Metastatic rectal cancer<br>Sclerosing cholangitis secondary to chemotherapy<br>Sarcoilitis<br>Nephrolithiasis<br>Recurrent cholangitis w/ biliary stents<br>Common bile duct stricturing	30-90 days	Never	Chemotherapy<br><br>Hypotensives	Antibiotics<br><br>Immunosuppressants	Deceased	10/22/14	Deceased	CMO	0	0	0	0	0	62	140.8	26
98	SICU	10/17/14	10/30/14	51 - 60	10/15/14	10/16/14	10/16/14	Elective right retrosigmoid craniotomy	Hypertension<br><br>Glaucoma<br><br>Many bladder surgeries	30-90 days	Never	Hypotensive	Antibiotics<br>Immunosuppressants	Floor	1/7/15	Rehab	Tracheostomy	0	0	0	1	0	63	143.4	25.2
99	SICU	10/20/14	10/30/14	31 - 40	10/20/14	10/20/14	10/20/14	Cholecystitis/Severe sepsis	Prediabetes	Uknown	Never	None		Floor	11/8/14	Rehab	Extubated	0	0	0	1	0	70	239.8	34.4

	SICU	10/22/14	10/23/14	18 - 30	10/22/14	10/22/14	10/22/14	Polytrauma s/p crushed by car	None	Never	Never	None		Floor	10/27/14	Home	Extubated	0	0	0	0	1	69	132	19.6
101	MICU	10/24/14	10/29/14	41 - 50	10/14/14	10/24/14	10/24/14	Jaundice/Left knee pain	Hepatitis C s/p transplant in 2013<br><br>ITP<br>Diabetes	Last 30 days	Never	Insulin	Antibiotics<br>Immunosuppressants<br>Insulin	Floor	12/11/14	Deceased	Extubated	0	0	0	0	0	66	136.4	22
102	MICU	10/24/14	10/30/14	61 - 70	10/23/14	10/23/14	10/23/14	Respiratory failure/lactic acidosis	Asthma<br><br>Diabetes<br>Hypertension<br>Depression	>1 year	Never	Statins	Anibiotics<br>Immunosuppressants	Floor	11/17/14	Rehab	Extubated		0	0	0	0	73	224.4	29.8
103	ICU	10/25/14	10/26/14	61 - 70	10/24/14	10/24/14	10/24/14	Acute respiratory failure/Seizures	Polysubstance abuse<br>Seizure<br>PTSD<br>Depression<br>GI bleed<br>Neuropathy	>1 year	Never		Antibiotics	Floor	10/27/14	Home	Extubated	1			0	0			
104	SICU	10/25/14	11/13/14	41 - 50	10/23/14	10/23/14	10/24/14	Fournier gangrene	Diabetes type I<br>Obesity<br>Hypertension<br>Diverticulitis<br>Asthma<br>COPD<br>PE<br>OSA<br>Hyperlipidemia<br>Depression	Unknown	Never	Anticoagulants<br>Statins<br>Insulin	Antibiotics	Deceased	11/12/14	Deceased	Deceased	0	0	0	1	0	72	497.2	67.5
105	ICU	10/26/14	10/31/14	61 - 70	10/25/14	10/25/14	10/25/14	Acute respiratory failure	Inoperable nonsmall cell lung cancer<br>COPD<br>Pneumonia<br>Neurofibromatosis<br>Hyperlipidemia<br>Osteoarthritis<br>s/p appendectomy<br>s/p hysterectomy	Last 30 days	Current	Statins<br>Antibiotics	Sedatives<br>Atibiotics	Outside hospital	10/31/14	Outside hospital	Hospital transfer	0	1	0	1	0	61	101.2	19.1
106	ICU	10/27/14	11/3/14	71 - 80	10/16/14	10/25/14	10/25/14	Bilateral inguinal hernia incarceration	CHF<br><br>s/p pericardial tamponade<br><br>Bilateral fem-fem bypass<br>Afib<br>Hypertension<br>CAD<br>Chronic kidney disease<br>Anemia<br>Dyslipidema<br>Recurrent PE<br>Hypothyroidism<br>Gout<br>BPH	Last 30 days	Never	Anticoagulants<br><br>Statins	Antibiotics<br>Immunosuppressants	Floor	11/11/14	Rehab	Extubated	0	0	1	1	0	68	180.8	27.5
107	MICU	10/28/14	11/7/14	61 - 70	10/26/14	10/26/14	10/27/14	Acute pancreatitis	COPD<br><br>ETOH dependence<br>Hypertension<br>Anxiety<br>Hyperthyroid	>1 year	Unknown	Statins<br><br>Steroids	Antibiotics<br>Immunosuppressants	Floor	11/12/14	Rehab	Extubated	1	1	0	0	0	63	179.5	31.9
108	MICU	10/28/14	11/2/14	71 - 80	10/18/14	10/28/14	10/28/14	Increasing left side subdural hematoma s/p fall	Multiple falls<br>Subdural hematoma<br>Diabetes<br>Hypertension<br>Hypercholesterolemia	Last 30 days	Never	Statins<br>Insulin	Antibiotics	Floor	11/7/14	Rehab	Extubated				1	1	63	184.6	32.8
109	ICU	5/23/15	5/31/15	71 - 80	5/15/15	5/22/15	5/22/15	Shortness of Breath	Asthma<br>COPD<br>CHF<br>DM<br>HTN<br>Thyroid Disease<br>Anemia<br>Aortic Valve disorder<br>Aspergillosis<br>Cellulitis	Last 30 days		Antibiotic		Deceased	5/30/15	Deceased	CMO	0	0	0	0	0	63	126	22.3
110	SICU	5/24/15	6/2/15	61 - 70	5/22/15	5/23/15	5/23/15	Renal Failure	CA prostate<br><br>Dysmetabolic Syndrom X<br>Hypogonadism<br><br>Obstructive Sleep Apnea<br>Extrinsic Asthma<br>COPD<br>Diastolic CHF<br>HTN<br>A-fib<br>Periphral Neuropathy<br>Morbid Obesity<br>Hyperlipedemia<br>DM	unknown	yes	Unknown	Unknown	Floor	6/9/15	Rehab	Extubated	1	0	1	0	0	66	243	39.2
111	MICU	11/1/14	11/6/14	61 - 70	10/27/14	10/27/14	11/1/14	Septic shock	Diabetes<br>CAD s/p CABG<br>Hyperlipidema<br>Obesity<br>Neuropathic pain<br><br>Peripheral vascular disease<br>Cardiomyopathy	Last 30 days	Never	Insulin	Antibiotics	Floor	11.20.14	Rehab	Extubated		1	0	0	0	70	209	30
112	ICU	11/1/14	11/5/14	71 - 80	10/31/14	10/31/14	10/31/14	Upper GI bleed	Diverticulitis<br>Colovaginal fistula<br><br>Acute blood loss anemia<br>Mallory-weiss tear<br>Esophageal varices<br>Diabetes<br>UTI<br>CHF<br>Thrombocytopenia	Last 30 days	Current	Insulin<br>Anticoagulants	Antibiotics	Floor	11/10/14	Deceased	Extubated	0	0	0	0	0	64	172.9	29.6
113	SICU	5/25/15	6/4/15	71 - 80	5/24/15	5/24/15	5/24/15	Acute Abdomen	AAA<br>CA Bladder<br>CAD<br>Kidney Disease<br>Neuropathy	Unknown	no	unknown		Floor	6/19/15	Rehab	Extubated	0	1	0	0	0	69	160	23.6
114	MICU	11/4/14	11/8/14	81 - 90	11/2/14	11/2/14	11/2/14	s/p PEA arrest	Interstitial lung disease<br>CHF<br>Hypertension<br>Chronic kidney disease<br>Diabetes<br>Hyperlipidemia<br>Hypothyroidism<br>Lumbar radiculopathy	30-90 days	Never	Statins<br>Insulin	Antibiotics	Deceased	11/7/14	Deceased	CMO	0	0	1	0	0	62	145.2	26.8
115	MICU	11/6/14	11/8/14	81 - 90	11/5/14	11/5/14	11/5/14	Acute hypoxemic respiratory failure/Shock	Hip fixation surgery<br>Myelofibrosis<br>Chronic anemia<br>CAD<br>Hypertension<br>Afib<br>CHF<br>GERD	Last 30 days	Current	Anticoagulants		Deceased	11/10/14	Deceased	Extubated	0	0	0	0	0	64	113.5	19.4

116	ICU	11/6/14	11/19/14	61 - 70	11/6/14	11/6/14	11/6/14	Hemmorhagic shock/GI bleed	Cryptogenic cirrhosis<br>Anemia<br>Hypothyroidism<br>Rectal cancer<br>Diverticulitis<br>Inguinal hernia<br>s/p TIPS	>1 year	Never			Deceased	11/19/14	Deceased	CMO	1	0	1	0	0	62	158.4	29.2
117	ICU	11/9/14	11/11/14	61 - 70	11/8/14	11/8/14	11/8/14	Acute respiratory failure/unresponsiveness	CAD<br><br>STEMI s/p stent placement<br>CHF<br>Hypertension<br>Hyperlipidemia<br>COPD	>1 year	Never	Statins	Antibiotics<br>Immunosuppressants	Floor	11/13/14	Rehab	Extubated	0	1	0	0	0	65	207.2	34.6
118	SICU	11/10/14	11/15/14	61 - 70	11/4/14	11/4/14	N/A	Polytrauma s/p mechanical fall	Lupus<br>PE<br>GERD<br>Afib	Unknown	Never	Steroids	Immunosuppressants		12/4/14	Acute Rehab	Tracheostomy	0	0	0	0	1	67	114.4	18
119	ICU	5/27/15	5/28/15	61 - 70	5/26/15	5/26/15	5/26/15	A-fib	DM type II<br>Diabetic Nephropathy<br>HTN<br>Metastatic CA Breast<br>Paraoxysomal A-fib<br>CAD<br>GOUT<br>CKD on HD<br>Pulmonary HTN	Unknown	Never	Amiodrone<br>Insulin<br>Statin<br>Anticoagulant<br>Denosumab		Floor	5/30/15	Home	Extubated	0	0	0	0	0	61	152	28.7
120	SICU	11/12/14	11/14/14	61 - 70	11/11/14	11/12/14	11/12/14	Parastomal hernia	COPD<br>CHF<br>Diabetes<br>Hypertension<br>Anemia<br>Chronic kidney disease<br>Right colon cancer	>1 year	Unknown	Statins<br>Antibiotics	Antibiotics	Floor	11/17/14	Home	Extubated	0	0	0	1	0	69	235.4	34.9
121	SICU	11/12/14	11/22/14	51 - 60	11/11/14	11/11/14	11/11/14	Polytrauma	Hypertension<br>Depression	Unknown	Never		Antibiotics		11/26/14	Home	Extubation	0	1	0	1	1	69	260.7	38.7
122	MICU	5/27/15	5/28/15	81 - 90	5/25/15	5/25/15	5/25/15	Bilateral Pneumonia	COPD<br>DM<br>A-fib<br>Subdural Hygroma<br>CO2 Retention<br>CHF<br>Subdural Hematoma<br>Hyperlipidemia<br>Depression<br>Emphysema<br>CA Prostate	Unknown	yes	Steriod<br>Anticoagulation<br>Insulin		Floor	6/1/15	Nursing Home	Extubation	0	0	1	0	0	67	226	35.4
123	SICU	11/13/14	11/22/14	81 - 90	10/30/14	11/12/14	11/12/14	Extruding bowel through defect	Colon resection 1 year ago	>1 year	Unknown	Steroids	Antibiotics<br>Immunosuppressants		12/4/14	Rehab	Extubation	0	0	0	1	0	70	187.7	26.9
124	SICU	11/13/14	11/24/14	51 - 60	11/10/14	11/10/14	11/10/14	Ruptured aneurysm	Unknown	Unknown	Never	Unknown	Antibiotics<br>Immunosuppressants		12/8/14	Rehab	Extubation				1		70	149.6	22.7
125	SICU	11/14/14	11/24/14	71 - 80	11/12/14	11/12/14	11/13/14	Oral cavity bleeding	Hyperlipidemia<br>Hypothyroidism<br>Anxiety<br>Basal cell carcinoma of nose<br>Benign prostatic hyperplasia<br>Prostate cancer<br>Epiglottic cancer<br>Base of tongue cancer	>1 year	Unknown	Probiotics	Antibiotics		11/28/14	Rehab	Extubation	0	0	1	0	0	73	150	19.9
126	SICU	11/14/14	11/19/14	41 - 50	11/4/14	11/4/14	11/14/14	Pancreatitis/ascites	Hypothyroidism<br>Hypertension<br>Hyperlipidemia<br>Diabetes<br>Alcohol cirrhosis	Last 30 days	Never	Insulin<br>Antibiotics		Deceased	11/19/14	Deceased	CMO	1	0	0	0	0	65	177.1	29.6
127	SICU	11/15/14	11/16/14	71 - 80	11/13/14	11/14/14	11/14/14	Ventral hernia repair	COPD<br>OSA<br>Type II diabetes<br>Hypothyroidism	30-90 days		Insulin<br>Statins	Antibiotics	Floor	11/24/14	Home w/VNA	Extubated	0	0	1	1	0	63	246.6	43.8
128	ICU	11/16/14	11/21/14	61 - 70	11/15/14	11/15/14	11/15/14	Cardiac arrest	Prostate cancer w/ bone metastasis<br>DVT<br>Cardiomyopathy<br>s/p cholecystectomy<br>Aspiration PNA	90 days-1 year	Unknown	Anticoagulants<br>Chemotheraputics			11/26/14	Rehab	Extubation	1	1	0	0	0	70	171.6	24.6
129	MICU	11/17/14	11/27/14	61 - 70	11/16/14	11/16/14	11/16/14	Myasthenic crisis/Acute respiratory failue	Myasthenia gravis<br>Type II diabetes<br>Hypertension<br>Hyperlipidemia<br>Chronic venous insufficiency<br>Obesity<br>Micronodular cirrhosis<br>Diverticula	Last 30 days	Never	Insulin	Immunosuppressants		12/5/14	Long-term assisted care facility	Tracheostomy	0	0	0	0	0	64	204.6	35
130	MICU	11/18/14	11/27/14	51 - 60	11/17/14	11/17/14	11/13/14	Septic shock/bacteremia	Seizure disorder	Unknown	Never		Antibiotics		11.27.14	Deceased	CMO	1	1	0	0	0	69	193.6	28.7
131	SICU	11/20/14	11/26/14	71 - 80	11/17/14	11/17/14	11/18/14	Septic shock/colitis/AKI/ acidosis	Chronic kidney disease<br>Ulcerative colitis<br>Anemia<br>Optic atrophy<br>Hypertension	<30 days	Current	Anticoagulants	Antibiotics	Floor	12/12/14	Long term care facility	Extubated	0	0	0	1	0	59	145	29.7
132	SICU	11/22/14	11/26/14	51 - 60	11/10/14	11/22/14	11/22/14	Hypoxia and new large left PE	Hep C cirrhosis<br>Depression	Unknown	Never	Omeprazole	Antibiotics	Floor	12/22/14	Rehab	Extubated	1	0	0	1	0	61	145	27.4
133	ICU	11/24/14	12/3/14	31 - 40	11/21/14	11/21/14	11/21/14	Acute respiratory failure/status epilepticus	ETOH abuse<br>Seizure diorder	Unknown	Never		Antibiotics	Floor	12/11/14	Rehab	Extubated	1	1	0	0	0	62	134.6	24.6
134	MICU	5/28/15	6/14/15	41 - 50	5/27/15	5/27/15	5/27/15	GI Bleed	Viral Hepatitis<br>Cirrhosis<br>EtOH Abuse<br>Varices<br>COPD	1 year	unknown	Steriod		Deceaseed	6/14/15	Deceased	CMO	1	1	0	0	0	70	242	34.7
135	MICU	5/28/15	5/29/15	41 - 50	5/27/15	5/27/15	5/27/15	Siezures	Seizures<br>PTSD<br>GERD<br>EtOH Abuse<br>Pancreatitis	30 days	Never	Unknown		Floor	6/11/15	Home	Extubated	0	1	0	0	0	72	165	22.4

136	MICU	5/30/15	5/31/15	61 - 70	5/29/15	5/29/15	5/29/15	Respiratory Failure	pneumonia<br>Pleural effusion<br>GERD	30-90 days	no	insulin<br>Nicotine Patch		Floor	6/11/15	Long Term Acute care Facility	Extubated		0	1			68	173	26.3
137	MICU	11/26/14	12/1/14	81 - 90	11/25/14	11/26/15	11/26/14	Acute respiratory failure	Diastolic heart failure<br>Afib<br>Chronic pain syndrome<br>Rheumatoid arthritis<br>GERD<br>Hyperlipidemia<br>Upper GI bleed	90 days-1yr	Never	Anticoagulants<br>Pain medication	Antibiotics	Floor	12/5/14	Skilled nursing facility	Extubated	0	0	0	0	0	59	204.6	41.3
138	ICU	11/28/14	11/30/14	18 - 30	11/27/14	11/27/14	11/27/14	Change in mental status/AKI	Unknown, substance abuse	Unknown	Unknown	Unknown	Antibiotics	Floor	12/3/14	Home	Extubated				0	0	72	202	27.5
139	SICU	6/18/15	6/25/15	71 - 80	6/17/15	6/17/15	6/17/15	Subdural Hematoma	Unknown	Unknown	Inknown	Anticoagulation<br>Statin		Deceased	6/25/15	Deceased	CMO	0	0	0	0	0	73	155	20.4
140	MICU	11/28/14	12/11/14	51 - 60	11/23/14	11/26/14	11/26/14	Hypercalcemia/right sided neoplasm	Obesity	Unknown	Never		Antibiotics<br>Immunosuppressants	Deceased	12/11/14	Deceased	CMO	0	1	0	0	0	60	206.8	40.7
141	MICU	11/28/14	12/5/14	71 - 80	11/27/14	11/27/14	11/27/14	Trauma s/p fall	Breast cancer w/ metastasis to liver and lungs<br>Sciatica<br>Hypertension<br>Diabetes	30-90 days	Never	Pain medication		Hospital transfer	12/4/14	Hospital transfer	Hospital transfer	0	0	0	0	1	65	171.6	28.7
142	ICU	11/29/14	12/1/14	61 - 70	11/28/14	11/28/14	11/28/14	Acute respiratory failure/Septic shock	End stage renal disease<br>Cervial laminectomy<br>Hypertension<br>Hyperlipidemia<br>Diabetes type II<br>Anxiety<br>Depression<br>GERD<br>Colovaginal fistula s/p colostomy<br>CAD<br>Heart failure s/p pacemaker	90 days-1 year	Current	Anticoagulants<br>Pain medication	Antibiotics	Floor	12/9/14	Skilled nursing facility	Extubated	0	0	1	0	0	63	140	24.8
143	MICU	11/29/14	12/5/14	51 - 60	11/29/14	11/29/14	11/19/14	Acute respiratory failure/encephalopathy	Genotype 1a hepatitis C cirrhosis<br><br>Hepatocellular carcinoma<br>Anxiety<br>Chronic pain<br>IV drug abuse	90 days-1 year	Never	Pain medication		Floor	12/9/14	Home	Extubated	0	1	0	0	0	71	231	32.4
144	ICU	11/30/14	12/2/14	81 - 90	11/26/14	11/30/14	11/30/14	s/p fall/acute renal failure/right PE	CAD w/ LAD stent<br>CHF<br>Afib<br><br>Multiple falls/contusions<br><br>Right bundle branch block<br>Hypertension<br>Patent foramen ovale<br>Chronic kidney disease<br><br>Myelodysplastic anemia<br><br>Subarachnoid hemorrhage<br>Hiatal hernia<br>Spinal stenosis<br>Gout<br>BPH	30-90 days	Never	Statins		Floor	12/17/14	Home with VNA	Extubated	0	0	0	1	1	66	172.7	27.8
145	ICU	11/30/14	12/20/14	51 - 60	11/29/14	11/29/14	11/29/14	s/p Cardiac arrest	Diabetes Type I<br>Hyperlipidemia<br>Chronic back pain<br>Hypertension<br>Retinopathy	Unknown	Never	Statins<br>Insulin		Deceased	12/20/14	Deceased	CMO		1	0	0	0	69	169.4	25
146	SICU	11/30/14	12/12/14	81 - 90	11/29/14	11/30/14	11/30/14	Subarachnoid hemorrhage s/p fall	Hypertension<br>Hypercholesterolemia<br>Afib<br>Appendectomy<br>Cholecystectomy<br>Hyterectomy<br>CHF	Unknown	Unknown	Lopressor		Deceased	12/12/14	Deceased	CMO					1	58	207.9	43.7
147	SICU	11/30/14	12/3/14	71 - 80	11/26/14	11/29/14	11/29/14	Small bowel obstruction	Hypertension<br><br>Hypothyroidism<br>Rheumatoid arthritis<br>Diverticulitis<br>Diabetes	>1 year	Unknown	Steroids	Antibiotics<br>Immunosuppressants	Floor	12/9/14	Rehab	Extubated	0	0	0	1	0	62	259.2	47.8
148	SICU	12/1/14	12/12/14	61 - 70	11/28/14	11/28/14	11/30/14	Spinal cord hemorrhage	Pantocytopenia<br>Hepatitis C<br>Porphyria cutanea<br>Upper GI bleed	Unknown	Unknown			Deceased	12/17/14	Deceased	Tracheostomy	1	1	0	0	0	72	176.4	23.9
149	SICU	12/5/14	12/14/14	61 - 70	12/1/14	12/4/14	12/4/14	Elective urinary diversion	CAD<br><br>Peripheral vascular disease<br>Cervical cancer<br>ARDS<br>Hypothyroidism<br>Urinary incontinence<br>GERD<br>Peripheral neuropathy<br>Cervical radiculopathy<br>Vitamin B12 deficieny<br>Hyperlipidemia<br>Hypertension<br>Laparoscopic cholecystectomy	Unknown	Unknown	Statins	Antibiotics	Floor	12/20/14	Rehab	Extubated						64	160.6	27.5
150	SICU	12/3/14	12/12/14	61 - 70	12/2/14	12/2/14	12/3/14	Pneumonia/Sepsis	Hemochromatosis and Hepatopulmonary syndrome s/p liver donor transplant in 2007<br>Bilateral ORIF<br>Right middle lobe adenocarcinoma<br>Reccurent upper left lobe pneumonia<br>Diabetes type II<br>Hypertension<br>Asthma<br>C.diff colitis<br>Tonsillectomy	>1 year	Never	Lopressor<br>Pain medication	Antibiotics	Rehab	12/17/14	Rehab	Tracheostomy	0	0	1	0	0	62	137.7	25.4

151	MICU	12/6/14	12/9/14	61 - 70	12/5/14	12/5/14	12/5/14	Acute respiratory failure/Encephalopathy	Breast cancer w/ wide spread metastasis<br><br>Asthma<br>Glaucoma<br>Hypothyroidism	30-90 days	Never	Chemotheraputics	Antibiotics<br>Immunosuppressants	Floor	12/30/14	Hospice	Extubated	0			0	0	63	141.7	25.2
152	ICU	12/3/14	12/10/14	41 - 50	12/2/14	12/2/14	12/2/14	Anterior wall STEMI/cardiogenic shock	Upper extremity abscess	Unknown	Unknown	None		Floor	12/17/14	Home	Extubated	0	1	0	0	0	70	180.4	25.9
153	SICU	12/7/14	12/9/14	71 - 80	12/3/14	12/3/14	12/6/14	Elective ureteroscopy cytoscopy laster lithotripsy	Hypertension<br>Dyslipidemia<br><br>Symptomatic bradycardia treated with pacemaker<br>BPH<br>Seizure disorder<br>Mild Parkinson's<br>Hyperthyroidism	<30 days	Never	Statins	Immunosuppressants	Floor	12/15/14	Rehab	Extubated	1	0	0	1	0	64	174.9	29.9
154	SICU	6/2/15	6/4/15	61 - 70	6/1/15	6/1/15	6/1/15	Carcinoma Tongue	Asthma<br>COPD<br>HTN<br>Hyperlipidemia<br>A-fib and flutter<br>Arthritis<br>polycythemia	less than 30 days	Never	Unknown		Floor	6/9/15	Home with Care	Extubated	1	0	1	0	0	69	212	31.3
155	MICU	12/7/14	12/16/14	71 - 80	12/3/14	12/3/14	12/6/14	Respiratory distress/Fever	Hypothyroidism<br><br>Cholecystectomy s/p colon resection for cancer<br>AML	<30 days	Never			Deceased	12/16/14	Deceased	CMO	0	0	0	0	0	62	194	35.8
156	ICU	12/8/14	12/14/14	41 - 50	12/8/14	12/8/14	12/8/14	Right sided weakness/facial droop	Hypertension<br>Depression<br>ETOH abuse	Unknown	Never	Lopressor		Deceased	12/14/14	Deceased	CMO	1	0	0	0	0	62	220	40.6
157	MICU	12/10/14	12/12/14	61 - 70	12/9/14	12/9/14	12/9/14	Acute respiratory failure/Shock	Hypertension<br>Chronic kidney disease 2/2 diabetes<br>Hyperlipidemia	Unknown	Never	Lopressor<br><br>Insulin		Floor	12/23/14	Rehab	Extubated	1	0	0	1	0	66	106.7	17.2
158	MICU	6/3/15	6/14/15	71 - 80	5/29/15	6/2/15	6/3/15	Aphasia	Colectomy	Unknown	not before			Floor	6/19/15	Nursing Home	Extubated	1	0	1	0	0	72	213	28.9
159	SICU	12/13/14	12/15/14	81 - 90	12/9/14	12/9/14	12/9/14	Polytrauma	Type II Diabetes<br>Hypertension<br>GERD<br>Anemia	Unknown	Unknown	Hypoglycemics	Antibiotics	Floor	12/23/14	Rehab	Extubated						62	169.4	31.2
160	MICU	12/15/14	12/17/14	61 - 70	12/15/14	12/15/14	12/15/14	Acute respiratory failure/Sepsis	COPD<br>Type II Diabetes<br>Hypertension<br>Lung nodule that is PET positive	>1year	Never	Statins<br>hypoglycemics		Floor	12/20/14	Home	Extubated	0	0	1	0	0	66	156	25.2
161	MICU	12/16/14	12/21/14	51 - 60	12/15/14	12/16/14	12/16/14	Right basal ganglia hemorrhage	Unknown	Unknown	Unknown			Deceased	12/21/14	Deceased	CMO						70	226.6	32.5
162	MICU	6/3/15	6/7/15	71 - 80	5/29/15	6/3/15	6/3/15	Hip Fracture	Asthma<br>Carcinoma kidney<br>Heart Transplant<br>Arthritis<br>DM<br>GERD<br>HTN<br>Diabetic Nephropathy<br>CHF<br><br>Esophageal reflux diseas	less than 30 (	Never	Statins<br>Immunosuppresent		Deceased	6/7/15	Deceased	CMO	1	0	1	0	0	67	145	22.7
163	ICU	12/19/14	12/24/14	51 - 60	12/12/14	12/16/14	12/17/14	Altered mental status/Acute kidney injury	Large C-cell lymphoma w/ renal involvement<br><br>Left third nerve paralysis and right brachial plexus<br>Type II diabetes<br>Hyperlipidemia<br>Gout<br>Hypertension<br>Anemia	90days-1year	Never		Antibiotics<br><br>Immunosuppressants	Deceased	12/25/14	Deceased	CMO		0	0	0	0	74	308	39.2
164	SICU	12/21/14	1/8/15	51 - 60	12/17/14	12/20/14	12/19/14	Right lower extremity ischemia and non dopplerable pulses	Diabetes<br>Hypertension<br>CAD<br>Hyperlipidemia<br>COPD<br>Diabetic Retinopathy<br>OSA<br><br>Peripheral vascular disease	>1 year	Unknown	Statins<br>Antibiotics<br>Insulin<br>Steroids<br>Anticoagulants		Long term care facility	1/30/15	Long term care facility	Tracheostomy	1	0	1	1	0	68	264	40.1
165	MICU	12/21/14	12/23/14	61 - 70	12/20/14	12/20/14	12/20/14	Hypoxic respiratory failure/Shock	COPD<br><br>Electrolyte abnormalities 2/2 SIADH<br>ETOH hepatitis	<30 days	Unknown	Vasopressors		Floor	1/22/15	Long term care facility	Extubated	1	0	1	0	0	66	136.4	22
166	MICU	12/27/14	1/1/15	51 - 60	12/26/15	12/26/15	12/26/14	Acute renal failure/Shock	End stage liver disease 2/2 ETOH cirrhosis<br>Esophageal variceal bleeding<br>Portosystemic encephalopathy<br>Chronic ascites and peripheral edema	90days-1year	Never		Antibiotics	Floor	2/11/15	Long term care facility	Extubated	1	0	1	1	0	71	338.8	47.5
167	ICU	12/28/14	1/7/15	51 - 60	12/7/14	12/26/14	12/27/14	Purulent discharge	SLE<br><br>Rheumatoid arthritis<br>Glaucoma<br>Hyperlipidemia<br>Hypertension<br>Depression<br><br>IgA lambda and kappa monoclonal gammopathy<br>s/p cholecystectomy<br>s/p appendectomy x2	Unknown	Never	Statins	Antibiotics<br>Immunosuppressants	Floor	1/15/15	Rehab	Extubated	0	0	1	1	0	64	242	41.4
168	MICU	12/28/14	1/1/15	71 - 80	12/26/14	12/28/14	12/28/14	Community acquired pneumonia	CAD s/p CABG<br>Hypertension<br>Dyslipidemia	>1 year	Unknown	Lopressor	Antibiotics	Deceased	12/30/14	Deceased	CMO	0	0	1	1	0	65	227.7	38
169	SICU	12/29/14	1/6/15	61 - 70	12/27/14	12/27/14	12/28/14	Trauma s/p MVA	Type II Diabetes	90days-1year	Last 30 days	Statins		Rehab	1/13/15	Rehab	Extubated	1	0	1	0	1	66	165.3	26.7

									Afib<br>CVA			Anticoagulants													
170	ICU	12/29/14	1/12/15	51 - 60	12/29/14	12/29/14	12/29/14	Acute hypoxemic respriatory failure/Shock	CHF<br>Nonischemic cardiomyopathy<br>Afib<br>COPD<br>Hypothyroidism<br>Dyslipidemia<br>Obesity<br>CKD<br>Chronic right pleural effusion<br>OSA<br>Gout<br>Lymphadenopathy and splenomegaly	Unknown	Never	Anticoagulants		Floor	1/30/15	Long term care facility	Extubated	0	0	0	1	0	66	264	42.5
171	ICU	1/1/15	1/4/15	71 - 80	12/27/14	12/31/14	12/31/14	Left hydropneumothorax	Head and neck cancer<br><br>Diabetes<br>Pneumonia<br>Dysphagia 2/2 head and neck cancer<br>BPH<br>s/p pancreatectomy<br>Left leg DVT	Unknown	Never	Insulin<br><br>Immunosuppressants	Antibiotics<br>Immunosupp ressants	Deceased	1/3/15	Deceased	CMO	1	0	1	0	0	72	165	22.4
172	MICU	12/30/14	1/22/15	71 - 80	12/30/14	12/30/14	12/30/14	Altered mental status and hypoxemia	Epilepsy<br>CAD<br>BPH<br>Anxiety<br>Pneumonia<br>Ulcerative colitis	Unknown	Unknown	Statins		Deceased	1/22/15	Deceased	CMO				0	0	70	164.6	23.6
173	SICU	12/30/14	1/4/15	71 - 80	12/28/14	12/29/14	12/29/14	Dysphagia	Afib<br>Anemia<br><br>CHF<br>Dysphagia<br>Esophageal stenosis<br>Gout<br>Arthrosis of the knee<br>Hypertension<br>Psoriasis<br>Cataracts	Unknown	Never	Antibiotics<br>Anticoagulants<br><br>Immunosuppressants		Long term care facility	1/26/15	Long term care facility	Tracheostomy	0	0	1	1	0	67	198.4	31.2
174	SICU	12/30/14	1/1/15	71 - 80	12/28/14	12/29/14	12/29/14	Change in mental status following mechanical fall	None known	Unknown	Unknown	None	Antibiotics	Floor	1/13/15	Skilled nursing facility	Extubated				1	1	73	134.2	17.8
175	SICU	12/30/14	1/4/15	61 - 70	12/27/14	12/29/14	12/29/14	Combined systolic and diastolic heart failure/UTI	CHF<br>Afib<br>Cardiomyopathy<br><br>Metastatic breast cancer<br>Anemia<br>Metabolic acidosis<br><br>Chronic renal insufficiency<br>Morbid obesity	Last 30 days	Last 30 days	Anticoagualants	Antibiotics	Floor	1/8/15	Rehab	Extubated	0	0	1	0	0	63	218.3	38.7
176	SICU	12/30/14	1/4/15	31 - 40	12/27/14	12/29/14	12/29/14	Cellulitis	Type II Diabetes, insulin dependent<br>Bipolar disorder<br>Hyperlipidemia<br>GERD<br>Chronic back pain	Unknown	Never	Insulin<br>Statins	Antibiotics	Floor	1/7/15	Home w/ VNA	Extubated	0	1	0	1	0	71	220	30.9
177	MICU	1/3/15	1/6/15	81 - 90	1/2/15	1/2/15	1/2/15	Dyspnea and GI bleed	CVA and right MCA distribution<br>Seizures<br>Hypertension<br>Hypercholesterolemia<br>COPD<br>Anxiety<br>Enterococcal UTI<br>MRSA positive sputum	Unknown	Current	Statins<br>Steroids		Floor	1/7/15	Deceased	Extubated	0	0	0	0	0	64	149.6	25.6
178	ICU	1/3/15	1/6/15	71 - 80	12/31/14	1/2/15	1/3/15	New onset atrial flutter and rapid ventricular response	Hyperlipidemia<br>Hypertension<br>Osteoarthritis<br>Trigeminal neuroalgia<br>Open-angle glaucoma	>1 year	Unknown	Statins		Floor	1/14/15	Skilled nursing facility	Extubated	0	0	0	0	0	61	158.4	30
179	MICU	6/4/16	6/16/15	41 - 50	5/22/15	5/22/15	6/3/15	Altered Mental Status	Epilepsy<br>Concussion<br>Convulsions<br>Spasmatic torticollis<br>Constipation	Unknown	Never	Gabapentin<br>Divalproex		Long term care fa	7/2/15	Long term care facility	Tracheostomy	0	1	0	0	0	62	134	24.5
180	MICU	1/4/15	1/7/15	41 - 50	1/4/15	1/4/15	1/4/15	Drug overdose	OCD, Bipolar disorder x2 overdose<br>Cholecystectomy<br>Gastric bypass<br>Hypertension	>1 year	Never	Anticoagulants<br>Antibiotics		Floor	1/16/15	Psych hospital	Extubated	1	1	0	0	0	N/A	136.4	N/A
181	MICU	6/6/15	6/27/15	51 - 60	5/28/15	6/5/15	6/5/15	Sepsis	HTN<br>Depression<br>Bipolar<br>Thyroid Disease	less than 30 days		Antibiotic Topical<br>Divalproex<br>Antiepileptic		Deceased	6/27/15	Deceased	CMO	0	1	0	0	0	68	200	30.4
182	ICU	1/7/15	1/10/15	51 - 60	12/30/14	1/6/14	1/6/15	Mechanical fall/Acute kidney injury	Type II diabetes<br><br>s/p cholecystectomy<br>Arthritis<br>Right leg superficial thrombophlebitis<br><br>Right peroneal neuropathy<br>Anxiety/depression<br>Morbid obesity	Unknown	Never	Insulin<br><br>Statins	Antibiotics<br>Immunosupp ressants	Floor	1/15/15	Rehab	Extubated		1	0	0	1	66	415.6	66.9
183	ICU	1/7/15	1/28/15	41 - 50	1/5/15	1/6/15	1/6/15	Panniculitis/Cellulitis/ hyperglycemia	Morbid Obesity<br>GERD<br>Hyperlipidemia<br>Hypertension<br>OSA<br>Type II diabetes	Unknown	Never	Insulin	Antibiotics	Long term care facility	2/6/15	Long term care facility	Tracheostomy	0	0	0	0	0	64	455.4	77.9
184	MICU	1/11/15	1/15/15	41 - 50	1/6/15	1/6/15	1/11/15	Upper GI bleed	ETOH hepatitis<br>Psoriasis<br>Bipolar	30-90 days	Unknown		Antibiotics	Deceased	1/14/15	Deceased	CMO	1			0	0	61	145.4	27.5

185	MICU	1/11/15	1/13/15	71 - 80	1/10/15	1/10/15	1/10/15	Ischemic stroke/Acute respiratory failure	Gout<br>Dyslipidemia<br>Prostate cancer<br>Vitamin D deficiency<br>s/p appendectomy<br>s/p inguinal hernia repair<br>s/p prostatectomy	>1 year	Unknown	Statins	Antibiotics	Floor	1/14/15	Home w/ VNA	Extubated	1	0	1	0	0	71	198	27.8
186	SICU	1/12/15	2/5/15	41 - 50	1/12/15	1/12/15	1/12/15	Acute respiratory failure/Septic shock	COPD with alpha-1 antitrypsin deficiency<br>Chronic hypoxemic respiratory failure<br>Pleurisy<br>GERD<br>Urinary retention<br>PTSD<br>Orthostatic hypotension	Last 30 days	Unknown			Rehab	3/4/15	Rehab	Tracheostomy		0	1	1	0	62	202.4	37.3
187	MICU	6/8/15	6/19/15	51 - 60	6/7/15	6/7/15	6/7/15	Seizures	EtOH Abuse<br>Depression<br>Mood disorder	Unknown	No			Floor	6/25/15	Other Hospital	Extubated	0	0	0	0	0	67	183	28.7
188	SICU	1/16/15	1/20/15	51 - 60	1/14/15	1/14/15	1/14/15	Epidural abscess	COPD<br>CAD<br>Diabetes<br>Hypertension<br>TIA<br>IV drug abuse	>1 year	Unknown	Anticoagulants<br>Insulin	Antibiotics	Floor	1/29/15	Rehab	Extubated				1	0	72	237.6	32.2
189	SICU	1/17/15	1/21/15	18 - 30	1/13/15	1/13/15	1/13/15	Multiple trauma	None	Never	Never	None	Antibiotics	Floor	2/2/15	Rehab	Extubated				1	1	74	193.6	24.9
190	MICU	1/17/15	1/29/15	31 - 40	1/15/15	1/15/15	1/16/15	Hypercapnic hypoxic respiratory failure	Panniculitis<br>Splenomegaly<br>Diabetes<br>Schizophrenia<br>Hypertension<br>Hyperlipidemia<br>GERD<br>COPD<br>Morbid obesity	>1 year	Never	Unknown	Antibiotics<br>Immunosupp<br>ressants	Hospital transfer	1/28/15	Hospital Transfer	Hospital Transfer	0	1	0	0	0	72	499.4	67.7
191	ICU	1/18/15	2/1/15	71 - 80	1/17/15	1/17/15	1/17/15	Respiratory failure	Bipolar disorder<br>Asthma<br>Hypothyroidism<br>Hypertension<br>Tremor	Unknown	Never	Antibiotics	Antibiotics<br>Immunosupp<br>ressants	Floor	2/10/15	Deceased	Extubated	0	0	0	0	0	65	193.6	32.3
192	SICU	1/19/15	1/22/15	51 - 60	1/18/15	1/18/15	1/18/15	Sigmoid volvulus	Sigmoid volvulus s/p colonoscopic decompression<br>Autism<br>Constipation<br>Asthma	>1 year	Current		Antibiotics	Floor	1/29/15	Back to group home	Extubated				1	0	69	176	26.1
193	SICU	6/9/15	6/10/15	61 - 70	5/28/15	6/8/15	6/8/15	Wound Dehiscence	CHF<br>CAD<br>DM<br>HTN<br>CKD	less than 30 (	no	Statin<br>Antibiotic<br>Insulin<br>Anticoagulation		Deceased	6/10/15	Deceased	Deceased	0	0	1	1	1	71	218	30.4
194	ICU	1/22/15	1/24/15	71 - 80	1/21/15	1/21/15	1/21/15	Acute massive pulmonary emboli/Acute respiratory failure	Lewy body dementia<br>Right acute subdural hematoma<br>Left lower extremity DVT<br>Hyponatremia<br>UTI	>1 year	Never		Antibiotics	Deceased	1/23/15	Deceased	CMO	0	0	1	0	0	62	132	24.3
195	SICU	1/29/15	2/7/15	71 - 80	1/3/15	1/27/15	1/28/15	Large bowel obstruction	Hypothyrodism<br>GERD	>1 year	Unknown		Antibiotics	Floor	2/27/15	Home	Extubated	0	0	1	1	0	67	158.6	24.9
196	SICU	1/31/15	2/14/15	51 - 60	1/26/15	1/26/15	1/29/15	Cystectomy and ileal loop	Hypertension<br>Chronic kidney disease s/p transplant in 2002<br>Hypothyroidism<br>Seizure disorder<br>Anemia<br>BKA nephropathy<br>Bladder cancer	Never	Unknown	Immunosuppressants	Antibiotics	Long term care facility	2/18/15	Long term care facility	Tracheostomy		0	0	1	0	65	123.2	20.6
197	SICU	2/1/15	2/4/15	61 - 70	1/31/15	1/31/15	1/31/15	Chronic aneurysmal changes with a contained rupture of right common iliac aneurysm	Hypertension<br>End stage renal disease s/p radiocephalic AV fistula<br>rheumatoid arthritis<br>TIAs and CVAs<br>Type B aortic dissection, s/p aortic fenestration	Unknown	Never		Antibiotics<br>Immunosupp<br>ressants	Floor	2/20/15	Home	Extubated	0	0	1	1	0	73	136.4	18.1
198	MICU	2/8/15	2/16/15	> 91	2/6/15	2/8/15	2/8/15	Heart failure exacerbation	Hypertension<br>CKD stage I secondary to hypertension<br>Edema	Unknown	Never	Lopressor		Floor	2/24/15	Deceased	Extubated		0	0	0	0	63	120	21.3
199	MICU	2/8/15	2/26/15	81 - 90	2/6/15	2/7/15	2/5/15	Acute on chronic diastolic heart failure/Rapid Afib with rapid ventricular response	Paroxysmal Afib<br>Rheumatoid arthritis<br>Hypertension<br>Pericarditis<br>Moderate to severe aortic stenosis<br>Diastolic CHF	Last 30 days	Current	Lopressor<br>Anticoagulant<br>Antibiotics	Antibiotics	Floor	3/5/15	Skilled nursing facility	Extubated	0	0	1	0	0	65	176	29.4
200	ICU	2/8/15	2/16/15	18 - 30	2/6/15	2/7/15	2/6/15	Septic shock/Acute respiratory failure	ADHD<br>Opiate abuse<br>Depression/anxiety<br>Borderline personality disorder<br>OSA<br>GERD<br>Recurrent respiratory tract infections	Unknown	Never		Antibiotics	Floor	2/23/15	Rehab	Extubated	1			0	0	68	319	48.4
201	MICU	2/11/15	2/20/15	81 - 90	2/10/15	2/10/15	2/10/15	Acute hypoxemic respiratory failure	Multiple myeloma<br>Hypertension<br>Prostate cancer<br>Osteoporosis<br>Peripheral neuropathy<br>Psoriasis	>1 year	Unknown		Antibiotics	Floor	2/26/15	Rehab	Extubated	0	0	1	0	0	67	187	29.4

202	MICU	2/11/15	2/13/15	61 - 70	2/6/15	2/11/15	2/11/15	Right hepatic artery aneurysms/Sepsis/Acute respiratory failure	Hyperlipidemia<br>Grand mal seizures<br>Depression/PTSD	Unknown	Never	Anticoagulants	Antibiotics	Deceased	2/12/15	Deceased	CMO	0	0	0	0	0	67	157.7	24.8
203	SICU	6/12/15	6/13/15	51 - 60	6/12/15	6/12/15	6/12/15	Trauma<br>Found on Floor<br>Unconcious	Unknown	Unknown	No	Unknown		Deceased	6/13/15	Deceased	CMO	0	0	0	0	1	68	111	16.9
204	SICU	2/13/15	2/17/15	61 - 70	2/12/15	2/12/15	2/12/15	Fournier gangrene	Liver cirrhosis<br>Esophageal varices<br>Hypertension<br>Hyperlipidemia<br>Morbid obesity	Unknown	Unknown	Statin	Antibiotics	Rehab	2/26/15	Rehab	Extubated	0	0	0	1	0	74	310.2	39.9
205	ICU	2/14/15	2/18/15	61 - 70	2/13/15	2/13/15	2/13/15	Right mainstem broncus mass/Hypoxic respiratory failure	Asthma<br>Hypertension<br>Hyperlipidemia<br>CAD s/p MI in 2004<br><br>Ischemic cardiomyopathy<br>Intracardiac apical thrombus<br><br>Hereditary spherocytosis	Unknown	Never	Antibiotics	Antibiotics	Floor	2/25/15	Home w/ VNA	Extubated	0	0	1	0	0	71	195.8	37.5
206	SICU	2/16/15	2/23/15	81 - 90	2/10/15	2/14/15	2/14/15	Closure of ileostomy	Benign prostatic hypertrophy<br>Nephrolithiasis<br>Ischemic colitis<br>CVA<br>CAD<br>Diverticulitis<br>Hypothyroidism<br><br>Chronic renal insufficiency	90 days-1 year	Unknown	Statins<br>Probiotics	Antibiotics	Floor, then back to SICU	3/21/15	Deceased	Extubated		0	1	1	0	69	173.8	25.8
207	MICU	2/17/15	2/20/15	41 - 50	2/16/15	2/16/15	2/16/15	Acute respiratory failure/Shock	Hypertension<br><br>Congenital abnormality of the urethra	Unknown	Never		Antibiotics	Floor	2/25/15	Home w/ VNA	Extubated	0	0	0	0	0	59	174.2	35.2
208	SICU	2/18/15	2/26/15	81 - 90	2/17/15	2/17/15	2/17/15	Elective right shoulder arthroplasty	Heart failure<br><br>Angina<br>Afib<br>Embolic CVA<br>Multinodular goiter<br>Uterovaginal prolapse<br>Anxiety/depression<br>Appendectomy<br>Hip replacement	>1 year	Unknown	Anticoagulants	Antibiotics<br>Immunosuppressants	Floor	3/9/15	Home w/ VNA	Extubated	0	0	0	1	0	65	118.8	19.6
209	SICU	2/21/15	3/3/15	61 - 70	2/20/15	2/20/15	2/19/15	Explorative laparotomy and partial colectomy	Diabetes<br>OSA<br>Morbid obesity<br><br>Hypothyroidism<br>Hypertension<br>Recent MRSA infection	30-90 days	Current		Antibiotics<br>Pressors<br>Sedation<br>Anticoagulants	Floor	3/10/15	Long term care facility	Extubated	0	0	0	1	0	66	330	53.1
210	SICU	2/27/15	3/5/15	61 - 70	2/19/15	2/19/15	2/19/15	Ruptured abdominal aortic aneurysm<br>Left	Hypertension<br>BKA<br>s/p cataract surgery	Unknown	Unknown	Statins	Antibiotics	Long term care facility	3/13/15	Long term care facility	Tracheostomy	0	1	0	1	0	70	242	34.7
211	SICU	2/27/15	3/5/15	61 - 70	2/26/15	2/26/15	2/26/15	Small bowel obstruction	Laparoscopic bypass surgery<br>Diverticulitis s/p colon resection<br>Neck surgery<br>GI bleed	Unknown	Never	Statins	Antibiotics	Floor	3/12/15	Rehab	Extubated	1	0	0	1	0	68	215.6	32.7
212	SICU	2/27/15	3/12/15	41 - 50	2/24/15	2/24/15	2/24/15	Intraparenchymal hemorrhage	Alcoholic cirrhosis	Unknown	Unknown			Long term care facility	3/17/15	Long term care facility	Tracheostomy	1				1	64	158.2	27.1
213	MICU	3/3/15	3/12/15	71 - 80	2/26/15	3/3/15	3/3/15	Acute hypoxic respiratory failure	AKI<br>Hypercalcemia<br>Developmental delay<br>Legally blind<br>Multiple myeloma<br>Diastolic CHF<br>Hypertension<br>Endometrial polyposis<br>Obesity	Last 30 days	Current	Lopressor	Antibiotics	Floor	3/14/15	Deceased	Extubated	0	0	0	0	0	65	194.5	32.5
214	MICU	3/5/15	3/9/15	51 - 60	3/5/15	3/5/15	3/5/15	Intracranial hemorrhage	Diabetes<br>Cirrhosis: NASH vs. alcoholic<br>Obesity<br>OSA<br>Hypertension<br>Dyslipidemia	Unknown	Never	Lopressor<br><br>Antibiotics	Antibiotics	Deceased	3/8/15	Deceased	CMO	1			1	0	68	356.4	54.1
215	MICU	3/9/15	3/13/15	18 - 30	3/8/15	3/8/15	3/8/15	Acute respiratory failure/Cardiac arrest/Drug OD	Drug abuse	Unknown	Never	None		Deceased	3/13/15	Deceased	Deceased	0	1	0	0	0	71	148.3	20.7
216	MICU	3/10/15	3/14/15	41 - 50	3/7/15	3/7/15	3/7/15	Altered mental status/Sepsis/Pneumonia	Seizures<br>Hypertension<br>Alcohol abuse<br>Liver disease<br>Bipolar	Unknown	Never	Lopressor	Antibiotics	Floor	3/20/15	Rehab	Extubated	1			0	0	68	162.8	24.7
217	SICU	3/10/15	3/21/15	51 - 60	3/7/15	3/8/15	3/9/15	Necrotizing soft tissue infection	Hypertension<br><br>GERD<br>Alcohol abuse<br>Left hip replacement<br>Depression	>1 year	Unknown	Lopressor	Antibiotics<br>Immunosuppressants		4/10/15	Rehab	Extubated	1			1	0	72	209	28.7
218	SICU	3/15/15	3/25/15	71 - 80	3/15/15	3/15/15	3/15/15	Polytrauma s/p fall	Esophageal cancer s/p esophagectomy<br>COPD<br>Stomach cancer	Unknown	Unknown				3/31/15	Acute Rehab	Extubated					1			
219	SICU	3/15/15	3/28/15	61 - 70	3/5/15	3/7/15	3/15/15	Deceased donor liver transplant	Hepatitis C<br><br>Hepatocellular carcinoma s/p transarterial chemoembolizatoin	Last 30 days	Never	Antibiotics	Immunosuppressants		5/12/15	Long term care	Tracheostomy	1	0	1	1	0	68	200.2	30.4

									Cirrhosis<br>Bipolar disorder<br>Esophageal varices<br>Hepatic encephalopathy<br>Spontaneous bacterial peritonitis<br>Gout<br>Hypertension<br>OSA																
220	SICU	6/14/15	6/19/15	71 - 80	6/5/15	6/6/15	6/9/15	Flial Chest<br>Pneumothorax<br>Trauma	Pneumonia<br>UTI<br>Asthma<br>Pulmonary Emphysema<br>Periphral neuropathy<br>Hep C infection	Unknown	Never				6.24.15	Long term care	Tracheostomy	1	1	0	0	1	70	144	20.66
221	ICU	3/18/15	3/30/15	71 - 80	3/17/15	3/17/15	3/17/15	Acute hypoxemic respiratory failure	Dementia<br>Dysphagia<br>Diabetes<br>Hypothyroidism<br>Hypertension	30-90 days	Current	Antibiotics<br>Lopressor			4/5/15	Rehab	Self-Extubated						52	98.8	25.8
222	SICU	3/19/15	3/29/15	61 - 70	3/19/15	3/19/15	3/19/15	Obstructive left ureteral stone/Sepsis	Primary progressive multiple sclerosis<br>Dysphagia<br>Depression<br>PTSD<br>Neurogenic bladder<br>Herpes zoster<br>Hypothyroidism<br>Hypertension	>1 year	Unknown	Statins	Antibiotics		4/3/15	Assisted living	Extubated	0	0	0	0	0	65	176	29.4
223	MICU	3/21/15	3/24/15	61 - 70	3/20/15	3/20/15	3.20.15	Acute hypoxemic respiratory failure	Pulmonary hypertension<br>Asthma<br>Diabetes<br>Diastolic CHF<br>Anxiety/Depression<br>CAD<br>Chronic renal insufficiency<br>Hypertension<br>Bilateral ischemic stroke<br>Paget disease	90 days-1 year	Never		Antibiotics<br>Immunosupressants	Floor	3/28/15	Rehab	Extubated						65	196.9	32.9
224	ICU	3/22/15	3/27/15	51 - 60	3/21/15	3/21/15	3.21.15	Hypoxic respiratory failure	CVA with residual hemiplegia<br>Recurrent Aspiration Pneumonia<br>Cirrhosis(EtOH and Hep-C)<br>COPD	30-90 days	Never	Antibiotic<br>Bronchodialator		Deceased	3.26.15	Deceased	Deceased						66	154	24.9
225	MICU	3/22/15	4/8/15	51 - 60	3/21/15	3/21/15	3/22/15	Flu<br>Pneumonia	HTN<br>Hypokalemia<br>Obesity	unknown	never			Long term Care	4/30/15	Long term Care	Tracheostomy	0	0	1	0	0	68	260	39.5
226	SICU	3/26/15	3/28/15	61 - 70	3/24/15	3/24/15	3/25/15	Recurrent diverticulitis<br>Septic shock<br>Peritonitis	Prolapsed mitral valve leaflet syndrome<br>Diverticulitis of colon<br>GERD<br>Atrial fibrillation	unknown	Never			Deceased	4/22/15	Deceased	Extubated	0	0	0	0	0	65	116	19.3
227	SICU	6/16/15	6/19/15	18 - 30	6/14/15	6/14/15	6/15/15	Epidural hematoma	NASH<br>Depression<br>Anorexia<br>Alcoholism<br>RLS	Unknown	Never				7/1/15	Acute Rehab	Extubated	1	1	1	1	1	70	131	18.8
228	MICU	3/26/15	4/7/15	61 - 70	3/25/15	3/25/15	3/25/15	Acute respiratory failure with hypoxia<br>Status post cardiac arrest<br>Flu pneumonia	ALS<br>Multiple myeloma<br>Peripheral neuropathy	Unknown		Antibiotic<br>Immunosuppressant		Long term acute care	4/29/15	Long term acute care	Tracheostomy	0	0	0	0	0	68	162	24.6
229	MICU	4/6/15	4/9/15	61 - 70	4/5/15	4/5/15	4/1/15	Acute respiratory failure<br>Shock<br>Renal failure	Alcohol abuse<br>Cirrhosis<br>Hepatitis C<br>Peripheral Vascular disease<br>COPD<br>CAD	30 days	Unknown	Antibiotic<br>Steroids		Deceased	4/9/15	Deceased	CMO	1	1	1	0	0	72	183	24
230	ICU	4/6/15	4/16/15	41 - 50	4/5/15	4/5/15	4/5/15	Hepatic failure	Hypothyroidism<br>Hemochromatosis carrier	30 days	Yes	Antibiotic		Long term care	5/11/15	Long term care	Extubated	1	0	0	0	0	65	181	30.2
231	ICU	4/6/15	4/9/15	41 - 50	4/5/15	4/5/15	4/5/15	Alcoholic cirrhosis with ascites	Alcoholic cirrhosis<br>VRE<br>Esophageal varices	30 days	Never			Deceased	4/9/15	Deceased	CMO	1	0	1	0	0	72	225	30.5
232	SICU	4/8/15	4/25/15	81 - 90	4/6/15	4/7/15	4/7/15	Ruptured AAA	Hypertension<br>Hypercholesterolemia<br>Depression	Unknown	Never	Unknown		Long term care	5/6/15	Long term care	Tracheostomy	1	1	1	1	0	73	202	27
233	SICU	4/8/15	4/16/15	71 - 80	4/2/15	4/2/15	4/2/15	AAA	Arthritis<br>Prostate cancer<br>Aortic Stenosis<br>Peripheral Vascular disease<br>Sleep apnea<br>GERD<br>DM	30 days	Never	Statin		Long term care	4/30/15	Long term care	Tracheostomy	1	0	1	0	0	69	207	30.6
234	ICU	4/10/15	4/17/15	41 - 50	4/9/15	4/10/15	4/10/15	Pneumonia	Alzheimer disease<br>Development delay<br>Bundle Branch Block	30-90 days		Donepezil		Deceased	4/16/15	Deceased	CMO	1	0	0	0	0	60	143	27
235	SICU	4/12/15	4/14/15	71 - 80	4/10/15	4/11/15	4/11/15	Lumbar Radicculopathy	Sciatica	Unknown				step-down	4/22/15	Rehab	Extubated	0	0	0	0	0	62	243	44.4
236	ICU	4/14/15	4/17/15	61 - 70	4/13/15	4/13/15	4/13/15	Respiratory Failure	CVA<br>CAD<br>HTN<br>Ischemic Cardiomyopathy	30-90 days	never	Statin		floor	4/19/15	Home self care	Extubated	0	0	0	0	0	72	292	39.6
237	ICU	4/15/15	4/25/15	71 - 80	4/14/15	4/15/15	4/14/15	Encephalopathy<br>AcuteRespiratory failure<br>Shock	COPD<br>Seizure disorder<br>CKD<br>HTN<br>BPH<br>recurrent UTI	90 days to 1 year	Yes	Statin		Floor	5/5/15	other hospital	Extubated	0	0	1	0	0	70	271	38.9
238	SICU	4/15/15	5/3/15	81 - 90	4/13/15	4/13/15	4/13/15	SBO	Ischemic Bowel Disease<br>DM<br>HTN<br>Heart failure rEF<br>BPH<br>Urethral stricture	90 days to 1 year	Yes	anticogulant		Deceased	5/3/15	Deceased	CMO	0	0	0	0	0	70	186	30.82
239	ICU	4/16/15	4/30/15	71 - 80	4/14/15	4/15/15	4/15/15	Flu<br>Weakness	HTN<br>Obesity hypoventilation Syndrom<br>DM	90 days to 1 year	never	Statin<br>Insulin<br>Lopressor		Deceased	4/29/15	Deceased	CMO	0	0	1	0	0	64	277	47.5

									PVD<br>CAD															
240	ICU	4/16/15	4/18/15	41 - 50	4/14/15	4/14/15	4/14/15	Hypercapneic Respiratory Failure<br><br>Altered Mental Status	COPD<br>Cellulitis<br><br>Proteinuria<br>DM<br>HTN<br>Sleep Apnea<br>Venous insufficiency	30-90 days	yes	Insulin	Floor	4/21/15	Other Health Care Facility	Extubated	0	0	0	0	0	70	270	38.7
241	ICU	4/16/15	4/18/15	71 - 80	4/15/15	4/15/15	4/15/15	Septic Shock<br>Pneumonia<br>Acute Resoiratory Failure<br>AKI	HTN<br>Dyslipidemia	Unknown	Never	Statins	Floor	4/26/15	Home with Health Care services	Extubated	0	1	0	0	0	63	118	20.9
242	MICU	5/13/15	5/19/15	61 - 70	5/12/15	5/12/15	5/12/15	Respiratory failure with Hypoxia	Obesity hypoventilation syndrom<br>A-fib<br>CAD s/p CABG<br>CHF<br>DM	Unknown	Yes	Statin<br>Insulin<br>Anticoagulant<br>Lopressor	Floor	6/8/15	Nursing Home	Extubated	0	0	0	0	0	70	314	42.6
243	MICU	5/14/15	5/16/15	71 - 80	4/30/15	5/12/15	5/12/15	COPD	COPD<br>CHF<br>DM<br>HTN<br>Hyperlipidemia<br>Asthma	Unknown	Never	Antibiotic<br>Insulin<br>Steriods	Deceased	5/15/15	Deceased	CMO	0	0	1	0	0	62	194	35.5
244	SICU	4/18/15	4/25/15	18 - 30	4/11/15	4/17/15	4/17/15	Sepsis<br>Right leg infection	DM<br>Asthma<br>Morbid Obesity<br>HTN<br>Venous Insuffciency b/l lower extremities	Never	Never	albuterol	Deceased	4/24/15	Deceased	Deceased	1	0	0	0	0	70	511	73.3
245	ICU	4/20/15	4/25/15	51 - 60	4/19/15	4/19/15	4/20/15	Liver Cirrhosis<br><br>Hepatorenal Syndrom	Alcoholic Cirrhosis<br><br>Hypothyroidism<br>HLD<br>Esophageal varices	30 days	Never	Nadolol	Deceased	5/3/15	Deceased	Extubated	1	1	0	0	0	64	157	26.9
246	SICU	4/23/15	5/8/15	51 - 60	4/22/15	4/22/15	4/22/15	Liver Failure	Cirrhosis<br>Chronic Hep C<br>Ascites<br>Anemia<br>DM<br><br>Hepatic Encephalopathy	90 days to 1 year		Antibiotic<br>Insulin	Rehab	5/21/15	Rehab	Tracheostomy		0	1			61	108	20.4
247	MICU	4/29/15	5/1/15	41 - 50	4/27/15	4/27/15	4/27/15	Acute Respiratory failure	HTN<br>Diastolic heart failure<br>Paraoxysomal atrial fib<br>CKD<br>Brain Leision<br>Seizures<br>Immune deficiency disorder	30 days	Yes	Statin<br>Antibiotic<br>Steriods<br>Anticoagulation	Floor	5/9/15	Rehab	Self Extubated	1	0	0			70	220	27.3
248	MICU	4/29/15	5/1/15	71 - 80	4/13/15	4/20/15	4/24/15	Shortness of breath<br><br>Interstial Lung Disease	Arthritis<br><br>Asthma<br>COPD<br>DM<br>h/o transfusion	>1 year	never	Statin<br><br>Salmetrol Inhaler	Deceased	5/1/15	Deceased	Deceased	1	0	0			64	176	30.2
249	MICU	5/7/15	5/15/15	51 - 60	5/3/15	5/4/15	5/4/15	Hyponatremia	HTN<br>GOUT<br>Dyslipedemia	Unknown	never before	Fibrate<br>Statin<br>ACE inhibitor	Floor	5/22/15	Nursing Home	Extubated	1	0	1			74	199	25.5
250	MICU	5/8/15	5/24/15	71 - 80	5/8/15	5/8/15	5/8/15	Sepsis	COPD<br>HTN<br>A-fib<br>Morbid Obesity<br>Cellulitis<br>Anemia<br>GERD<br>Depression	Last 30 days	Acute Rehab	Antibiotic<br>Salmetrol Inhaler<br>Insulin<br>Anticogulation	Long Term Care	5/28/15	Long Term Acute Care	Tracheostomy						72	275	37.3
