# Supplementary material for: The utility of endotracheal aspirate bacteriology in identifying mechanically ventilated patients at risk for ventilator associated pneumonia: a single-center prospective observational study
Source: BMC Infect Dis. 2019 Aug 29;19:756. doi: 10.1186/s12879-019-4367-7 (PMC6716855; doi:10.1186/s12879-019-4367-7)
Supplement: Supplementary file 8 — Daily observations, VAP diagnosis and ETA culture results. (PDF 109 kb) [file 12879_2019_4367_MOESM8_ESM.pdf]

	ID	Date of VAP assessment	Presence of (new or progressive) infiltrate on CXR or CT scan	Leukocyte count	Temperature / daily high	Purulent respiratory secretions	Temperature Fahrenheit	CLINICAL VAP	SAUR	Other	NBAL (only >10^4 shown)	BAL (only >10^4 shown)
Format / value(s)		mm/dd/yy	No<br>Yes<br>Yes (not new or progressive)<br>Yes (progressive)	xx.x	xx.x	No<br>Yes<br>N/A	xx.x					
Type (S = Source)		Derived	Derived	S	Derived, converted from Fahrenheit	Derived (CPIS score)						
	001	17/06/14	Yes (not new or progressive)	14.04	37.5	Yes	99.5		0	3+ HFLU		
		18/06/14	Yes (not new or progressive)	14.75	37.2	Yes	98.9		0	0		
		19/06/14	Yes (not new or progressive)	8.01	37.5	No	99.5		0	0		
		20/06/14	N/A	9.19	37.2	No	98.9		0	0		
	002	18/06/14	No	6.29	36.9	No			0	0		
		19/06/14	No	11.45	36.4	No			0	0		
		20/06/14	No	7.97	37.0	No			1+	0		
	003	19/06/14	Yes (not new or progressive)	18.8	37.4	No	99.3		0	0		
		20/06/14	Yes (not new or progressive)	17.64	37.4	No	99.4		N/A	N/A		
		21/06/14	Yes (not new or progressive)	15.6	37.5	No	99.5		N/A	N/A		
		22/06/14	Yes (not new or progressive)	14.28	38.6	No	101.5		N/A	N/A		
		23/06/14	Yes (not new or progressive)	14.1	37.9	Yes	100.2		0	0		
		24/06/14	Yes (not new or progressive)	16.65	37.9	Yes	100.2		0	0		
		25/06/14	Yes (not new or progressive)	16.63	37.6	No	99.7		1+	0		
		26/06/14	Yes (not new or progressive)	13.06	37.4	N/A	99.3		N/A	N/A		
	004	19/06/14	No	9.99	37.4	Yes	99.4		0	3+ ENTC		
		20/06/14	No	13.58	37.6	No	99.6		N/A	N/A		
	005	20/06/14	Yes (not new or progressive)	8.98	37.4	No	99.4		0	0		
		21/06/14	Yes (not new or progressive)	13.58	37.2	No	98.9		0	0		
		22/06/14	Yes (not new or progressive)	21.52	36.9	No	98.4		0	0		
		23/06/14	Yes (not new or progressive)	24.65	37.2	Yes	99		0	0		
		24/06/14	Yes (not new or progressive)	14.1	36.9	No	98.5		N/A	N/A		
		25/06/14	Yes (not new or progressive)	16.56	37.0	Yes	98.6		0	0		
		26/06/14	Yes (not new or progressive)	17.01	36.9	Yes	98.5		0	0		
		27/06/14	Yes (not new or progressive)	16.47	37.6	No	99.6		N/A	N/A		
		28/06/14	Yes (not new or progressive)	17.34	38.2	No	100.8		0	0		
		29/06/14	Yes (not new or progressive)	17.38	37.2	No	99		0	0		
		30/06/14	Yes (not new or progressive)	17.29	37.2	Yes	99		0	0		
		01/07/14	Yes (not new or progressive)	15.95	37.7	Yes	99.9		0	0		
		02/07/14	Yes (not new or progressive)	16.9	38.1	Yes	100.5		0	0		
	006	24/06/14	No	30.63	37.8	No	100		0	0		
		25/06/14	No	28.56	37.8	Yes	100		0	0		
		26/06/14	No	30.86	37.3	No	99.1		N/A	N/A		
		27/06/14	N/A	N/A	N/A	Yes			0	0		
		28/06/14	N/A	N/A	N/A	N/A			N/A	N/A		
	007	25/06/14	Yes (not new or progressive)	16.17	37.8	Yes			0	3+ KP		
		26/06/14	Yes (not new or progressive)	15.78	37.7	No			0	3+ KP		
		27/06/14	Yes (not new or progressive)	17.85	38.0	No			N/A	N/A		
		28/06/14	Yes (not new or progressive)	15.54	37.7	Yes			0	2+ KP		
		29/06/14	Yes (not new or progressive)	15.66	37.8	Yes	100		0	2+ KP		
		30/06/14	Yes (not new or progressive)	19.61	37.7	Yes	99.8		0	0		
	008	27/06/14	No	11.34	35.5	Yes			0	0		
		28/06/14	N/A	N/A	37.2	N/A	99		N/A	N/A		
	009	27/06/14	Yes (not new or progressive)	15.48	38.2	Yes			0	0		
		28/06/14	No	14.83	37.6	No			0	1+ ENTA; 2+ PSA		
		29/06/14	No	11.13	37.8	Yes			0	2+ ENTA; 2+ PSA		
		30/06/14	No	10.96	38.4	Yes			0	3+ ENTA; 2+ PSA; 4+ HFLU		
		01/07/14	Yes (new)	8.69	38.1	Yes		VAP	0	3+ ENTA; 2+ PSA		
		02/07/14	Yes	10.13	37.8	No	100.1		0	3+ ENTA		
		03/07/14	Yes	13.99	37.9	No	100.2		0	3+ ENTA; 3+ PSA		
		04/07/14	Yes	15.69	37.8	Yes	100	VAP	0	2+ ENTA; 2+ PSA		
		05/07/14	Yes	14.29	37.1	N/A	98.8		N/A	N/A		
	010	30/06/14	No	25.26	38.8	Yes	101.9		0	0		
		01/07/14	No	17.56	38.6	No	101.4		N/A	N/A		
		02/07/14	No	18.69	37.6	Yes	99.6		0	0		
		03/07/14	No	18.01	36.9	Yes	98.5		0	0		
		04/07/14	No	13.58	37.1	No	98.8		0	0		
		05/07/14	No	13.23	37.7	No	99.8		0	0		
		06/07/14	Yes (new)	18.52	37.6	No	99.6		0	0		
		07/07/14	Yes	20.18	37.2	No	99		0	0		
		08/07/14	Yes	21.21	38.1	Yes	100.6	VAP	0	0		
		09/07/14	Yes	19.54	38.0	Yes	100.4	VAP	0	0		
		10/07/14	Yes	21.38	38.3	Yes	101	VAP	0	0		
		11/07/14	Yes	22.84	37.2	Yes	98.9	VAP	0	0		
		12/07/14	Yes	15.97	37.2	No	98.9		0	0		
		13/07/14	Yes	15.81	37.1	No	98.7		0	0		
		14/07/14	Yes	14.22	36.7	N/A	98		N/A	N/A		
	011	02/07/14	No	15.3	38.1	No	100.6		0	0		
		03/07/14	Yes (new)	15.66	38.3	No			0	0		
		04/07/14	Yes	13.98	37.9	No			0	0		
		05/07/14	Yes	14.83	37.7	No			0	0		
		06/07/14	No	12.17	37.7	No			0	0		
		07/07/14	Yes (new)	14.84	38.0	No			0	0		
		08/07/14	Yes	15.21	35.2	Yes		VAP	0	0		
		09/07/14	Yes	26.78	38.2	Yes		VAP	0	0		
		10/07/14	Yes	20.91	37.4	No			0	0		
		11/07/14	N/A	N/A	N/A	N/A			N/A	N/A		
	012	04/07/14	Yes (not new or progressive)	11.35	37.5	No	99.5		0	2+ PSA		
		05/07/14	Yes (not new or progressive)	12.81	37.4	No	99.3		0	3+ PSA		
		06/07/14	Yes (not new or progressive)	12.64	37.2	No	99		0	2+ PSA		
		07/07/14	Yes (not new or progressive)	9.69	38.3	No	101		0	3+ EC; 3+ PSA		
		08/07/14	Yes (not new or progressive)	12.17	37.7	Yes	99.8		0	3+ EC; 3+ PSA		
		09/07/14	Yes (not new or progressive)	10.29	37.2	No	99		0	3+ EC; 3+ PSA		
		10/07/14	Yes (not new or progressive)	11.33	37.3	Yes	99.2		0	3+ EC; 3+ PSA		
		11/07/14	Yes (not new or progressive)	12.3	37.2	Yes	99		0	4+ EC; 4+ PSA		
		12/07/14	N/A	14.4	37.6	Yes	99.7		0	1+ EC; 4+ PSA		
		13/07/14	Yes (not new or progressive)	14.67	37.2	Yes	99		0	3+ PSA		
		14/07/14	Yes (not new or progressive)	12.96	36.8	Yes	98.2		N/A	N/A		
	013	04/07/14	Yes (not new or progressive)	3.54	37.4	Yes	99.4		0	3+ Asperg. Fum.		
		05/07/14	Yes (not new or progressive)	11.39	37.9	Yes	100.2		0	3+ Asperg. Fum.		
		06/07/14	N/A	9.68	32.6	N/A	90.7		N/A	N/A		
	014	06/07/14	No	22.18	29.1	No			N/A	N/A		
		07/07/14	Yes (new)	15.55	36.0	No			0	0		
		08/07/14	Yes	11.93	38.2	Yes			0	0		
		09/07/14	Yes (progressive)	10.35	38.3	No			N/A	N/A		
		10/07/14	Yes	10.41	38.1	Yes		VAP	0	0		
		11/07/14	No	9.21	38.3	Yes	100.9		3+	0		
		12/07/14	No	9.82	37.3	No	99.1		2+	0		
		13/07/14	No	9.97	39.1	No	102.3		2+	0		
		14/07/14	Yes (new)	10.07	38.6	Yes	101.4	VAP	2+	0		

	15/07/14	Yes	11.84	38.4	Yes	101.2	VAP	2+	0	
	16/07/14	Yes	11.35	38.4	Yes	101.2	VAP	4+	0	
	17/07/14	No	11.33	37.6	No	99.7		3+	0	
015	07/07/14	No	18.23	39.9	No			0	0	
	08/07/14	No	14.35	39.1	Yes			0	0	
	09/07/14	No	10.55	38.4	Yes			0	0	
	10/07/14	No	10.24	38.5	Yes			0	1+ ACCA	
	11/07/14	No	10.58	38.8	No	101.9		0	0	
	12/07/14	No	10.57	38.3	No	100.9		0	0	
	13/07/14	Yes (new)	12.59	38.6	No	101.5	VAP	0	0	
	14/07/14	Yes (progressive)	12.53	39.6	Yes	103.2	VAP	0	0	
	15/07/14	Yes	10.36	38.8	No	101.8		2+	2+ SPNE	
	16/07/14	N/A	N/A	N/A	No			0	0	
	17/07/14	N/A	N/A	N/A	No			0	0	
016	11/07/14	Yes (not new or progressive)	13.27	39.3	Yes	102.8		N/A	N/A	
	12/07/14	No	13.9	36.9	No	98.4		0	0	
	13/07/14	Yes (new)	14.59	37.1	No	98.8		0	0	
	14/07/14	Yes	12.71	37.1	Yes	98.8	VAP	0	0	
	15/07/14	N/A	12.22	36.6	N/A	97.9		N/A	N/A	
017	11/07/14	Yes (not new or progressive)	10.67	37.1	No	98.8		0	0	
	12/07/14	Yes (not new or progressive)	11.24	37.4	No	99.3		0	1+ KP	
	13/07/14	Yes (not new or progressive)	20.46	37.5	No	99.5		0	4+ KP	
	14/07/14	Yes (not new or progressive)	23.05	37.1	No	98.7		0	4+ KP	
	15/07/14	N/A	N/A	N/A	N/A			N/A	N/A	
018	13/07/14	No	3.25	32.8	Yes			0	0	
	14/07/14	Yes (new)	1.37	38.2	Yes			0	0	
	15/07/14	No	1.86	38.6	No			0	0	
	16/07/14	Yes (new)	1.87	38.0	No	100.4		0	0	
	17/07/14	Yes	2.31	39.4	Yes	103	VAP	0	0	
	18/07/14	No	4.04	39.0	Yes	102.2		0	0	
	19/07/14	No	3.73	37.9	No	100.3		0	0	
	20/07/14	No	2.38	39.3	No	102.7		0	0	
	21/07/14	No	1.57	38.3	No	101		0	0	
	22/07/14	No	0.76	37.2	No	98.9		0	0	
019	14/07/14	No	11.62	37.7	No			0	0	
	15/07/14	Yes (new)	9.86	38.0	No			0	0	
	16/07/14	Yes	8.18	38.3	No	101		0	0	
	17/07/14	Yes	10.15	37.9	No	100.2		0	0	
	18/07/14	Yes	10.02	37.4	No	99.4		0	0	
	19/07/14	Yes	11.14	38.1	No	100.5		0	0	
	20/07/14	Yes	8.84	38.2	Yes	100.7		0	0	
	21/07/14	Yes	12.05	38.2	No	100.8		0	0	
	22/07/14	No	12.37	38.9	No	102		0	0	
	23/07/14	Yes (new)	10.97	36.9	Yes	98.5		0	1+ mold	
	24/07/14	Yes	10.91	38.0	Yes	100.4		0	1+ mold	
	25/07/14	Yes	N/A	38.8	No	101.9		0	0	
020	20/05/15	No	22.9	38.8	Yes	101.8		0	0	
	21/05/15	N/A	21.53	37.9	N/A	100.2		N/A	N/A	
021	15/07/14	Yes (not new or progressive)	1.93	38.0	Yes	100.4		2+	0	
	16/07/14	Yes (not new or progressive)	3.21	39.5	No	103.1		4+	0	
	17/07/14	Yes (not new or progressive)	4.98	37.9	No	100.3		2+	0	
	18/07/14	Yes (not new or progressive)	6.31	38.0	No	100.4		0	0	
	19/07/14	No	7.69	37.8	No	100.1		2+	0	
	20/07/14	Yes (new)	11.22	39.0	No	102.2	VAP	1+	0	
	21/07/14	Yes	13.88	39.3	No	102.8	VAP	0	0	
	22/07/14	Yes	13.01	37.6	N/A	99.6		N/A	N/A	
	23/07/14	Yes	11.06	37.0	N/A	98.6		N/A	N/A	
022	15/07/14	Yes (not new or progressive)	20.86	36.9	No	98.5		3+	4+ PSA; 1+ KP	PSA, KP
	16/07/14	Yes (not new or progressive)	20.26	38.1	Yes	100.6		3+	4+ PSA; 2+ KP	
	17/07/14	Yes (not new or progressive)	25.25	38.7	Yes			3+	3+ PSA; 3+ KP	
	18/07/14	Yes (not new or progressive)	14.99	36.9	Yes			3+	3+ PSA	
	19/07/14	No	20.4	36.6	Yes	97.9		1+	1+ PSA; 1+ KP	
023	20/07/14	Yes (not new or progressive)	13.07	37.7	Yes	99.9		3+	0	
	21/07/14	Yes (not new or progressive)	26.62	37.3	No	99.1		2+	0	
	22/07/14	Yes (not new or progressive)	20.3	37.3	Yes	99.1		1+	0	
	23/07/14	Yes (not new or progressive)	14.49	37.1	No	98.8		0	0	
	24/07/14	Yes (not new or progressive)	10.95	37.4	Yes	99.4		0	0	
	25/07/14	Yes (not new or progressive)	8.57	37.6	Yes	99.6		0	0	
	26/07/14	Yes (not new or progressive)	8.75	37.8	No	100		0	0	
	27/07/14	Yes (not new or progressive)	5.95	38.6	No	101.5		0	0	
	28/07/14	Yes (not new or progressive)	5.91	37.8	No	100		0	0	
	29/07/14	Yes (not new or progressive)	3.68	38.4	No	101.1		0	0	
	30/07/14	Yes (not new or progressive)	3.77	38.0	Yes	100.4		0	0	
	31/07/14	Yes (not new or progressive)	5.2	37.5	Yes	99.5		0	0	
	01/08/14	Yes (not new or progressive)	3.43	37.4	No	99.4		0	0	
024	20/07/14	No	4.94	36.7	N/A	98		N/A	N/A	
	21/07/14	Yes (new)	9.12	36.9	Yes			0	0	
	22/07/14	Yes	5.03	37.3	No	99.2		0	0	
	23/07/14	No	3.67	37.4	Yes	99.3		0	0	
	24/07/14	Yes (new)	3.45	39.4	Yes	102.9	VAP	0	0	
	25/07/14	Yes	11.25	39.6	Yes	103.2	VAP	0	0	
	26/07/14	Yes	5.68	36.8	N/A	98.2		N/A	N/A	
025	20/05/15	Yes (not new or progressive)	15.48	37.7	No	99.9		0	0	
	21/05/15	Yes (not new or progressive)	10.77	37.9	Yes	100.3		0	0	
	22/05/15	Yes (not new or progressive)	8.91	37.8	N/A	100.1		N/A	N/A	
	23/05/15	N/A	N/A	N/A	N/A			N/A	N/A	
026	22/07/14	No	9.19	38.3	Yes	101		0	4+ KP	
	23/07/14	Yes (new)	7.76	37.9	Yes	100.3		0	3+ KP	
	24/07/14	Yes	9.17	38.1	Yes	100.6		0	1+ KP	
	25/07/14	Yes	6.51	37.4	N/A	99.3		N/A	N/A	
027	22/07/14	Yes (not new or progressive)	5.71	37.8	No	100		0	0	
	23/07/14	Yes (not new or progressive)	5.02	37.7	N/A	99.9		0	0	
	24/07/14	No	8.83	37.3	N/A	99.2		0	0	
028	22/07/14	Yes (not new or progressive)	38.18	37.2	Yes	99		0	0	
	23/07/14	Yes (not new or progressive)	25.41	37.0	N/A	98.6		N/A	N/A	
029	22/07/14	No	8.86	38.7	Yes	101.6		0	0	
	23/07/14	No	10.81	37.9	Yes	100.3		0	0	
	24/07/14	No	10.62	38.6	Yes	101.4		0	0	
	25/07/14	No	10.02	37.3	Yes	99.1		0	0	
	26/07/14	No	13.66	37.6	Yes	99.7		0	0	
	27/07/14	No	12.26	37.4	No	99.4		0	0	
	28/07/14	No	12.81	37.6	Yes	99.7		0	0	
	29/07/14	No	13.47	37.3	Yes	99.1		0	0	
	30/07/14	No	15.87	37.3	Yes	99.1		0	3+ HFLU	
030	25/07/14	Yes (not new or progressive)	7.06	37.6	Yes	99.7		3+	0	
	26/07/14	Yes (not new or progressive)	9.06	37.7	N/A	99.9		N/A	N/A	
	27/07/14	Yes (not new or progressive)	10.29	37.4	N/A	99.4		N/A	N/A	
031	25/07/14	Yes (not new or progressive)	16.92	37.7	Yes	99.8		N/A	N/A	
	26/07/14	Yes (not new or progressive)	N/A	37.2	Yes	98.9		0	0	
	27/07/14	Yes (not new or progressive)	12.24	37.2	No	98.9		0	0	
	28/07/14	Yes (not new or progressive)	13.35	37.6	Yes	99.6		0	0	
	29/07/14	N/A	11.44	37.8	N/A	100		N/A	N/A	
	30/07/14	No	10.62	37.7	Yes	99.8		0	0	

	31/07/14	No	10.71	37.8	Yes	100.1		0	0	
	01/08/14	No	16.28	37.4	No	99.4		0	0	
	02/08/14	No	11.34	36.6	N/A	97.8		N/A	N/A	
032	26/07/14	No	19.8	37.2	No	98.9		0	0	
	27/07/14	No	15.6	37.7	Yes	99.8		0	0	
	28/07/14	No	17.11	37.0	No	98.6		0	0	
	29/07/14	N/A	N/A	N/A	N/A			N/A	N/A	
033	31/07/14	No	30.46	37.1	Yes	98.7		0	0	
	01/08/14	No	23.5	37.0	No	98.6		0	3+ BKCC	
	02/08/14	No	20.34	36.9	Yes	98.5		0	3+ BKCC	
	03/08/14	No	22.14	37.6	Yes	99.7		0	3+ BKCC	
034	31/07/14	No	11.64	36.7	No	98		0	3+ Mold	
	01/08/14	No	17.06	38.3	No			0	3+ XMAL; 3+ Mold	
	02/08/14	No	13.08	37.6	Yes			0	3+ XMAL	
	03/08/14	No	14.21	37.4	No			0	4+ XMAL	
	04/08/14	No	10.8	36.9	Yes	98.4		0	4+ XMAL	
	05/08/14	No	12.17	37.2	Yes	99		0	4+ XMAL	
	06/08/14	No	18.64	37.0	N/A	98.6		N/A	N/A	
035	31/07/14	Yes (not new or progressive)	26.11	37.5	Yes	99.5		0	2+ HFLU	
	01/08/14	Yes (not new or progressive)	21.66	39.3	No	102.7		0	0	
	02/08/14	Yes (not new or progressive)	9.2	36.6	Yes	97.9		0	0	
	03/08/14	N/A	7.59	36.4	N/A	97.6		N/A	N/A	
036	03/08/14	N/A	14.8	39.4	Yes	103		0	0	
	04/08/14	No	9.42	37.7	No	99.8		0	0	
	05/08/14	No	12.45	38.0	No	100.4		0	0	
	06/08/14	No	9.26	38.1	Yes	100.6		0	0	
	07/08/14	No	9.71	38.1	Yes	100.6		0	0	
	08/08/14	Yes (new)	12.81	37.9	Yes	100.3	VAP	0	0	
	09/08/14	Yes	12.87	37.9	No	100.3		0	0	
	10/08/14	Yes	11.52	37.6	No	99.6		0	0	
	11/08/14	Yes	12.87	37.7	Yes	99.9	VAP	0	0	
	12/08/14	No	12.85	37.8	No	100.1		0	0	
037	07/08/14	Yes (not new or progressive)	15.07	37.5	N/A			N/A	N/A	
	08/08/14	N/A	9.82	37.4	Yes			0	0	
	09/08/14	Yes (not new or progressive)	15.84	37.1	No			0	0	
	10/08/14	Yes (not new or progressive)	18.41	37.6	Yes			0	0	
	11/08/14	Yes (not new or progressive)	22.85	37.4	No			0	0	
	12/08/14	No	25.43	37.2	No			0	0	
	13/08/14	No	22.86	37.4	No	99.3		0	0	
	14/08/14	No	21.24	39.1	Yes	102.3		0	0	
	15/08/14	Yes (new)	18.7	37.1	No	98.7		0	0	
	16/08/14	Yes	15.28	37.9	No	100.2		0	0	
	17/08/14	Yes	15.41	37.8	No	100		0	0	
	18/08/14	No	12.38	37.9	No	100.3		0	0	
	19/08/14	No	8.15	37.7	No	99.8		0	0	
	20/08/14	No	6.66	37.5	N/A	99.5		0	0	
	21/08/14	No	8	37.8	No	100		0	0	
	22/08/14	No	9.18	37.9	No	100.3		0	0	
	23/08/14	No	15.37	37.8	No	100		0	4+ PSA	
	24/08/14	Yes (new)	14.47	37.7	No	99.8		0	4+ PSA	
	25/08/14	Yes	14.72	37.2	Yes	99	VAP	0	4+ PSA-ECAP	
038	10/08/14	Yes (not new or progressive)	21.44	37.4	Yes	99.4		0	0	
	11/08/14	Yes (progressive)	17.59	37.2	Yes	99		0	0	
	12/08/14	Yes	9.01	37.3	No	99.2		0	1+ ENTA	
	13/08/14	No	13.17	37.4	Yes	99.4		0	1+ ENTC	
	14/08/14	No	12.83	37.4	No	99.3		0	1+ ENTC	
	15/08/14	No	13.57	37.7	Yes	99.8		0	2+ ENTC	
	16/08/14	N/A	16.72	37.3	N/A	99.2		N/A	N/A	
039	10/08/14	Yes (not new or progressive)	11.24	36.7	No			0	0	
	11/08/14	No	12.62	36.0	No			0	0	
	12/08/14	No	8.61	37.9	N/A			N/A	N/A	
	13/08/14	Yes (new)	12.7	37.4	No			0	0	
	14/08/14	Yes	14.71	35.7	No			0	0	
	15/08/14	Yes	20.16	36.5	Yes		VAP	0	0	
	16/08/14	No	15.87	36.6	N/A			N/A	N/A	
	17/08/14	N/A	N/A	N/A	N/A			N/A	N/A	
040	11/08/14	Yes (not new or progressive)	17.96	37.5	Yes	99.5		0	3+ ENTA	
	12/08/14	Yes (not new or progressive)	15.09	37.4	No	99.4		0	3+ ENTA	
	13/08/14	Yes (not new or progressive)	11.22	37.4	Yes	99.3		0	3+ ENTA	
	14/08/14	Yes (not new or progressive)	8.66	37.7	Yes	99.8		0	4+ ENTA	
	15/08/14	Yes (progressive)	11.16	37.9	Yes	100.3	VAP	0	4+ ENTA	
	16/08/14	Yes	13.61	38.1	No	100.5	VAP	0	3+ ENTA	
	17/08/14	No	14.24	37.8	Yes	100.1		0	3+ ENTA	
	18/08/14	No	11.17	37.2	No	98.9		0	3+ ENTA	
	19/08/14	No	11.01	37.8	Yes	100.1		0	3+ ENTA	
041	11/08/14	No	17.12	37.6	No	99.6		0	3+ ENTA	
	12/08/14	No	9.71	38.1	No	100.6		0	4+ ENTA	
	13/08/14	No	9.82	37.8	Yes	100.1		0	1+ ENTC	
	14/08/14	Yes (new)	8.44	37.3	Yes	99.2		0	2+ ENTC	
	15/08/14	Yes	8.95	38.0	Yes	100.4		0	2+ ENTC	
	16/08/14	Yes	9.67	38.2	No	100.7		0	2+ ENTC	
	17/08/14	Yes	11.27	38.0	Yes	100.4	VAP	0	3+ ENTC	
	18/08/14	No	12.57	38.7	Yes	101.6		0	0	
	19/08/14	No	13.6	37.0	No	98.6		0	2+ ENTC	
	20/08/14	Yes (new)	12.86	38.0	No	100.4		0	3+ ENTC	
	21/08/14	Yes (progressive)	12.64	38.9	Yes	102.1	VAP	0	3+ ENTC	Enterobacter
042	12/08/14	N/A	6.52	37.7	Yes	99.9		0	3+ KOX	
	13/08/14	No	6.45	37.9	Yes	100.3		0	1+ KOX	
	14/08/14	No	6.86	38.5	Yes	101.3		0	3+ KOX	
	15/08/14	No	6.58	37.8	Yes	100.1		0	1+ KOX	
	16/08/14	No	5.4	38.3	Yes	101		N/A	N/A	
	17/08/14	No	5.12	37.9	Yes	100.2		0	1+ KOX; 2+ ACHD	
	18/08/14	No	4.87	37.7	Yes	99.8		2+	1+ KOX; 3+ ACHD	
	19/08/14	No	4.58	37.8	Yes	100		0	0	
	20/08/14	No	5.38	39.0	Yes	102.2		1+	1+ KOX; 2+ ACHD	
	21/08/14	N/A	6.11	38.4	Yes	101.2		0	1+ KOX; 2+ ACHD	
	22/08/14	No	6.41	38.2	Yes	100.8		0	2+ KOX; 2+ ACHD	
	23/08/14	N/A	7.91	37.1	N/A	98.8		N/A	N/A	
043	14/08/14	Yes (not new or progressive)	13.4	36.9	No	98.4		0	0	
	15/08/14	Yes (not new or progressive)	22.54	36.6	No			0	0	
	16/08/14	N/A	N/A	N/A	N/A			N/A	N/A	
044	14/08/14	No	5.13	38.3	No	100.9		0	0	
	15/08/14	No	10.28	36.7	No	98		0	0	
	16/08/14	N/A	N/A	N/A	N/A			N/A	N/A	
045	16/08/14	Yes (not new or progressive)	27.5	37.3	Yes	99.1		0	3+ BTSF	
	17/08/14	Yes (not new or progressive)	23.15	37.5	Yes	99.5		0	0	
	18/08/14	Yes (not new or progressive)	N/A	37.4	No	99.3		0	0	
	19/08/14	Yes (not new or progressive)	37.36	37.3	No	99.1		0	0	
	20/08/14	Yes (not new or progressive)	37.42	37.9	Yes	100.2		0	0	
	21/08/14	Yes (not new or progressive)	35.77	37.4	Yes	99.3		0	0	
	22/08/14	Yes (not new or progressive)	60.55	37.2	Yes	98.9		0	0	
	23/08/14	Yes (not new or progressive)	39.89	37.7	No	99.8		0	0	
	24/08/14	Yes (not new or progressive)	40.31	37.9	No	100.3		0	0	

	25/08/14	Yes (not new or progressive)	30.71	37.7	Yes	99.8		0	0
	26/08/14	Yes (not new or progressive)	30.95	38.3	Yes	101		0	0
	27/08/14	Yes (not new or progressive)	54.61	37.5	Yes	99.5		0	0
	28/08/14	Yes (not new or progressive)	52.98	37.2	Yes	99		0	0
	29/08/14	Yes (not new or progressive)	40.19	38.2	Yes	100.8		0	0
	30/08/14	Yes (not new or progressive)	50.29	37.8	No	100		0	0
	31/08/14	Yes (not new or progressive)	50.58	37.3	Yes	99.2		0	0
	01/09/14	Yes (not new or progressive)	33.23	37.4	Yes	99.4		0	0
	02/09/14	Yes (not new or progressive)	28.46	37.5	Yes	99.5		0	0
	03/09/14	Yes (not new or progressive)	23.52	37.4	Yes	99.3		0	0
	04/09/14	Yes (not new or progressive)	16.8	37.1	No	98.7		0	0
046	21/05/15	Yes (not new or progressive)	19.87	37.1	Yes	98.8		0	0
	22/05/15	Yes (not new or progressive)	20.02	37.3	Yes	99.1		0	0
	23/05/15	Yes (not new or progressive)	16.47	37.8	N/A	100		N/A	N/A
047	18/08/01	Yes (not new or progressive)	14.43	37.3	Yes	99.1		0	0
	19/08/14	Yes (not new or progressive)	8.45	37.1	N/A	98.8		0	0
048	20/08/14	Yes (not new or progressive)	22.3	38.1	No	100.6		0	0
	21/08/14	No	23.14	38.2	Yes	100.8		0	0
	22/08/14	Yes (new)	25.5	39.5	Yes	103.1	VAP	0	1+ BTSC
	23/08/14	Yes	41.15	38.9	N/A	102.1	VAP	N/A	N/A
	24/08/14	Yes	34	37.9	N/A	100.2		N/A	N/A
	25/08/14	Yes	38.02	37.9	N/A	100.3		N/A	N/A
	26/08/14	Yes	44.74	39.2	N/A	102.5	VAP	N/A	N/A
049	21/08/14	Yes (not new or progressive)	38.99	37.0	No			0	0
	22/08/14	Yes (not new or progressive)	37.69	37.8	No			0	0
	23/08/14	Yes (not new or progressive)	35.28	38.2	No	100.8		0	0
	24/08/14	Yes (not new or progressive)	36.56	38.7	Yes	101.6		0	0
	25/08/14	N/A	N/A	N/A	N/A			N/A	N/A
050	22/08/14	Yes (not new or progressive)	16.99	36.6	Yes	97.8		N/A	N/A
	23/08/14	Yes (not new or progressive)	13.7	37.4	No	99.4		0	0
	24/08/14	Yes (not new or progressive)	12.87	37.1	Yes	98.8		0	0
	25/08/14	Yes (not new or progressive)	11.84	37.4	No	99.4		0	0
	26/08/14	Yes (not new or progressive)	12.75	36.7	N/A	98		N/A	N/A
	27/08/14	Yes (not new or progressive)	14.54	37.1	Yes	98.8		0	2+ EC
	28/08/14	Yes (not new or progressive)	16.43	37.7	Yes	99.8		0	0
	29/08/14	Yes (not new or progressive)	15.21	37.1	Yes	98.7		0	2+ EC
	30/08/14	Yes (not new or progressive)	13.97	37.2	No	98.9		0	2+ EC
	31/08/14	Yes (not new or progressive)	13.06	37.2	No	99		0	0
	01/09/14	No	13.84	37.2	Yes	98.9		0	1+ EC
	02/09/14	Yes (new)	16.43	36.9	No	98.4		0	1+ EC
	03/09/14	Yes	15.3	37.3	No	99.2		0	1+ EC
051	26/08/14	Yes (not new or progressive)	12.91	39.6	Yes	103.2		0	0
	27/08/14	Yes (not new or progressive)	12.18	38.0	Yes	100.4		0	0
	28/08/14	Yes (not new or progressive)	9.12	37.8	Yes	100		0	0
	29/08/14	Yes (not new or progressive)	12	37.6	N/A	99.6		N/A	N/A
052	26/08/14	Yes (not new or progressive)	9.4	38.8	Yes	101.8		0	0
	27/08/14	Yes (not new or progressive)	7.91	37.8	Yes	100.1		0	0
	28/08/14	Yes (not new or progressive)	7.41	37.4	Yes	99.4		0	0
	29/08/14	No	5.81	37.4	Yes	99.4		0	0
	30/08/14	Yes (new)	5.92	37.3	No	99.2		0	0
	31/08/14	Yes	7.85	37.2	Yes	99		0	0
	01/09/14	Yes	10.66	37.6	Yes	99.6		1+	0
	02/09/14	Yes (progressive)	13.47	37.6	N/A	99.7		N/A	N/A
053	27/08/14	No	20.8	37.2	N/A	99		N/A	N/A
	28/08/14	Yes (new)	17.15	37.2	No	99		0	3+ EC
	29/08/14	Yes (progressive)	41.83	38.4	Yes	101.2	VAP	0	4+ EC
	30/08/14	Yes	43.33	37.8	N/A	100.1		N/A	N/A
	31/08/14	Yes	44.41	38.5	No	101.3	VAP	1+	0
	01/09/14	Yes	21.24	37.6	Yes	99.7	VAP	0	2+ EC
	02/09/14	Yes	11.96	36.5	No	97.7		3+	0
	03/09/14	N/A	N/A	N/A	N/A			N/A	N/A
054	27/08/14	No	18.33	38.1	Yes	100.5		0	0
	28/08/14	Yes (new)	17.76	38.5	Yes	101.3		0	0
	29/08/14	No	12.77	37.9	Yes	100.2		0	0
	30/08/14	No	8.26	37.8	No	100.1		0	0
055	29/08/14	Yes (not new or progressive)	10.01	38.4	Yes	101.2		1+	0
	30/08/14	No	8.08	37.8	No	100		0	0
	31/08/14	Yes (new)	9.21	37.9	No	100.3		2+	0
056	30/08/14	No	15.95	37.2	Yes	98.9		0	0
	31/08/14	No	21.05	37.3	Yes	99.1		0	0
	01/09/14	No	21.48	37.4	Yes			0	0
	02/09/14	Yes (new)	15.34	36.8	Yes		VAP	0	0
	03/09/14	Yes	10.9	37.1	Yes			0	0
	04/09/14	Yes	11.01	37.0	No			0	0
	05/09/14	Yes	13.68	36.9	N/A	98.5		N/A	N/A
057	02/09/14	No	13.72	39.3	Yes	102.8		0	0
	03/09/14	No	13.75	37.4	Yes	99.4		0	0
	04/09/14	No	10.47	37.4	N/A	99.4		N/A	N/A
058	02/09/14	No	15.49	38.1	No	100.5		4+	0
	03/09/14	Yes (new)	10.67	38.6	Yes	101.5		4+	0
	04/09/14	Yes	11.29	38.5	Yes	101.3		2+	2+ EC
	05/09/14	Yes	8.87	39.4	Yes	102.9		0	0
	06/09/14	Yes	8.75	38.9	Yes	102		3+	3+ EC
	07/09/14	Yes	9.7	38.6	Yes	101.5		2+	1+ EC
	08/09/14	Yes	8.11	38.7	No	101.7		2+	1+ EC
	09/09/14	Yes	10.16	38.8	Yes	101.9		1+	1+ EC
	10/09/14	Yes	11.03	38.9	No	102		1+	1+ EC
	11/09/14	Yes	9.37	39.3	No	102.7		1+	2+ EC
	12/09/14	Yes	10.75	38.1	Yes	100.5		3+	0
	13/09/14	Yes	10.42	37.9	N/A	100.2		2+	0
	14/09/14	Yes	10.25	38.1	No	100.6		1+	0
	15/09/14	Yes	10.35	38.2	Yes	100.7		2+	0
	16/09/14	Yes	11.52	37.8	No	100		1+	0
059	03/09/14	Yes (not new or progressive)	26.6	37.0	N/A	98.6		N/A	N/A
	04/09/14	Yes (not new or progressive)	22.46	37.2	Yes	98.9		0	0
	05/09/14	Yes (not new or progressive)	15.13	37.4	Yes	99.4		0	0
	06/09/14	Yes (not new or progressive)	17.37	37.2	Yes	99		0	0
	07/09/14	Yes (not new or progressive)	18.11	37.4	No	99.4		0	0
	08/09/14	Yes (not new or progressive)	20.37	37.7	Yes	99.8		N/A	N/A
	09/09/14	Yes (not new or progressive)	19.33	38.4	Yes	101.2		0	0
	10/09/14	Yes (not new or progressive)	19.06	37.7	No	99.8		0	0
	11/09/14	Yes (progressive)	21.1	37.8	Yes	100	VAP	0	0
	12/09/14	Yes	19.34	38.4	Yes	101.2	VAP	0	0
	13/09/14	Yes	17.11	38.3	Yes	100.9	VAP	0	0
	14/09/14	Yes	19.91	37.9	N/A	100.3		N/A	N/A
	15/09/14	Yes	17.76	37.5	Yes	99.5	VAP	0	0
	16/09/14	Yes	14.86	37.3	Yes	99.2	VAP	0	0
	17/09/14	Yes (progressive)	15.58	37.8	No	100		N/A	N/A
	18/09/14	Yes	19.82	37.9	Yes	100.3	VAP	0	0
060	04/09/14	No	10.61	36.8	Yes	98.2		0	0
	05/09/14	No	10.97	37.4	Yes	99.3		0	0
061	05/09/14	No	14.41	37.4	Yes	99.3		N/A	N/A
	06/09/14	No	16.51	37.6	N/A	99.6		0	0

	07/09/14	No	15.6	37.9	No	100.2		0	0	
	08/09/14	Yes (new)	12.1	38.3	Yes	100.9	VAP	0	0	
062	09/09/14	Yes (not new or progressive)	15.16	37.8	No	100.1		N/A	N/A	
	10/09/14	Yes (not new or progressive)	20.71	37.8	No	100		0	0	
	11/09/14	No	20.26	38.4	No	101.1		0	0	
	12/09/14	No	15.04	38.4	Yes	101.2		0	0	
	13/09/14	No	12.18	39.1	Yes	102.4		0	0	
	14/09/14	Yes (new)	19.54	38.8	No	101.9	VAP	0	0	
	15/09/14	No	18.94	38.6	No	101.4		0	0	
	16/09/14	Yes (new)	33.59	37.8	No	100.1		0	0	
	17/09/14	Yes	38.01	36.7	No			0	0	
	18/09/14	N/A	N/A	N/A	N/A			N/A	N/A	
063	21/05/15	No	11.75	39.0	N/A	102.2		N/A	N/A	
	22/05/15	No	10.24	38.3	Yes	101		0	0	
	23/05/15	Yes (new)	15.2	38.4	Yes	101.2	VAP	0	0	
	24/05/15	No	12.62	37.8	Yes	100.1		0	0	
	25/05/15	Yes (new)	11.28	38.1	Yes	100.6	VAP	0	0	
	26/05/15	Yes	9.71	38.2	Yes	100.7	VAP	0	0	
	27/05/15	Yes	9.21	38.4	No	101.1		0	0	
	28/05/15	Yes	9.07	38.8	Yes	101.9	VAP	0	0	
	29/05/15	Yes	10.19	38.3	Yes	100.9	VAP	0	0	
	30/05/15	No	11.37	37.3	Yes	99.2		0	0	
	31/05/15	No	9.94	38.7	Yes	101.7		0	0	
	01/06/15	Yes (new)	15.17	38.0	Yes	100.4	VAP	0	0	
	02/06/15	Yes	14.46	37.9	No	100.2		0	0	
	03/06/15	Yes	16.03	37.6	No	99.7		0	0	
	04/06/15	Yes	11.3	37.7	N/A	99.8		N/A	N/A	
064	11/09/14	Yes (not new or progressive)	30.24	36.4	Yes	97.6		1+	0	
	12/09/14	Yes (not new or progressive)	33.82	37.6	Yes			0	0	
	13/09/14	Yes (not new or progressive)	20.33	37.1	Yes	98.8		0	0	
	14/09/14	Yes (not new or progressive)	16.71	37.0	N/A	98.6		N/A	N/A	
	15/09/14	Yes (not new or progressive)	20.46	37.2	Yes	99		0	0	
	16/09/14	Yes (not new or progressive)	19.83	36.7	Yes	98.1		0	0	
	17/09/14	Yes (not new or progressive)	19.29	36.4	N/A	97.6		N/A	N/A	
	18/09/14	Yes (not new or progressive)	24.71	37.7	Yes	99.8		0	0	
	19/09/14	Yes (not new or progressive)	25.66	36.7	No	98		0	0	
	20/09/14	Yes (not new or progressive)	15.31	37.0	No	98.6		0	0	
	21/09/14	Yes (not new or progressive)	19.88	36.7	Yes	98		0	0	
	22/09/14	Yes (not new or progressive)	21.01	36.7	No	98.1		0	0	
	23/09/14	Yes (not new or progressive)	18.01	37.7	N/A			N/A	N/A	
	24/09/14	Yes (not new or progressive)	15.73	36.9	No	98.5		0	0	
	25/09/14	Yes (not new or progressive)	15.22	37.1	N/A	98.7		N/A	N/A	
065	11/09/14	No	14.76	38.8	Yes	101.9		N/A	N/A	
	12/09/14	Yes (new)	14.99	37.4	Yes	99.3		0	0	
	13/09/14	Yes	7.39	37.3	N/A	99.1		N/A	N/A	
	14/09/14	Yes	8.34	37.8	N/A	100.1		N/A	N/A	
	15/09/14	No	6.51	36.7	Yes	98.1		0	0	
066	13/09/14	Yes (not new or progressive)	9.04	37.2	No	99		0	0	
	14/09/14	Yes (not new or progressive)	11.39	37.5	No	99.5		0	0	
	15/09/14	Yes (not new or progressive)	10.85	37.1	No	98.8		0	0	
	16/09/14	Yes (not new or progressive)	12.84	37.2	No	99		0	0	
	17/09/14	Yes (not new or progressive)	11.02	36.8	N/A	98.2		N/A	N/A	
067	19/09/14	No	11.89	38.0	N/A	100.4		N/A	N/A	
	20/09/14	No	14.21	37.6	No	99.6		0	0	
	21/09/14	Yes (new)	12.56	37.6	Yes	99.7	VAP	0	0	
	22/09/14	Yes	14.32	37.8	No	100		0	0	
	23/09/14	Yes	13.43	37.9	Yes	100.3	VAP	0	0	
	24/09/14	Yes	11.46	39.4	No	102.9	VAP	0	0	
	25/09/14	Yes	28.1	38.3	No	101	VAP	0	0	
	26/09/14	Yes	24.71	37.9	Yes	100.3	VAP	0	0	
	27/09/14	Yes	21.42	37.4	Yes	99.3	VAP	0	0	
	28/09/14	Yes	18.05	37.6	No	99.7		0	0	
	29/09/14	Yes	17.34	36.9	No	98.5		0	0	
	30/09/14	Yes	17.25	37.9	No	100.3		0	0	
	01/10/14	Yes	15.29	37.8	No	100.1		0	0	
	02/10/14	Yes	18.18	38.3	N/A	100.9	VAP	0	0	
	03/10/14	N/A	N/A	N/A	N/A			N/A	N/A	
068	19/09/14	No	14.34	37.0	N/A			N/A	N/A	
	20/09/14	No	8.7	37.8	No			0	1+ PSA	
	21/09/14	No	14.72	37.5	N/A			N/A	N/A	
069	20/09/14	No	20.68	37.9	Yes	100.3		0	0	
	21/09/14	No	11.89	37.7	Yes	99.8		0	3+ PSA; 3+ PSA (OMOR)	
	22/09/14	Yes (new)	6.05	37.1	No	98.8		0	2+ PSA; 2+ PSA (OMOR)	
	23/09/14	N/A	N/A	N/A	N/A			N/A	N/A	
070	21/09/14	No	18.4	37.6	Yes	99.7		0	0	
	22/09/14	No	16.03	37.4	No	99.3		0	0	
	23/09/14	No	13.12	37.1	N/A	98.8		N/A	N/A	
071	22/09/14	Yes (not new or progressive)	3.98	38.4	No	101.2		0	0	
	23/09/14	Yes (not new or progressive)	7.28	37.9	No			0	0	
	24/09/14	Yes (not new or progressive)	4.68	38.5	No			0	2+ EC	
	25/09/14	Yes (not new or progressive)	1.22	38.9	N/A			N/A	N/A	
	26/09/14	Yes (not new or progressive)	13.53	36.8	N/A			N/A	N/A	
072	21/05/15	Yes (not new or progressive)	14.49	37.8	Yes	100		1+	0	
	22/05/15	Yes (not new or progressive)	16.15	38.4	N/A	101.1		N/A	N/A	
	23/05/15	Yes (not new or progressive)	12.82	39.2	N/A	102.6		N/A	N/A	
073	25/09/14	Yes (not new or progressive)	11.88	38.0	No			0	0	
	26/09/14	No	12.4	38.4	Yes	101.1		0	0	
	27/09/14	No	15.97	37.3	Yes	99.2		0	0	
	28/09/14	No	8.08	37.6	Yes	99.7		3+	0	
	29/09/14	No	7.08	38.5	Yes	101.3		3+	0	
	30/09/14	No	12.7	39.2	Yes	102.6		3+	0	
	01/10/14	No	N/A	37.8	Yes	100.1		2+	0	SAUR
	02/10/14	No	13.96	39.2	No	102.6		0	0	
	03/10/14	No	17.49	38.2	Yes	100.8		0	0	
	04/10/14	Yes (new)	16.86	37.7	Yes	99.8	VAP	0	0	
	05/10/14	Yes	17.18	37.7	No	99.8		1+	0	
	06/10/14	Yes	17.32	38.2	No	100.8	VAP	3+	0	
	07/10/14	No	15.82	37.8	N/A	100		N/A	N/A	
074	25/09/14	No	12.48	37.4	No	99.3		0	2+ PSA	
	26/09/14	No	10.98	37.1	Yes	98.7		0	2+ PSA	
	27/09/14	No	10.07	38.3	Yes	100.9		0	3+ PSA	
075	26/09/14	Yes (not new or progressive)	8.94	38.4	Yes	101.1		2+	0	
	27/09/14	Yes (not new or progressive)	10.49	37.7	N/A	99.8		N/A	N/A	
076	27/09/14	Yes (not new or progressive)	13.75	34.1	Yes			N/A	N/A	HFLU
	28/09/14	Yes (not new or progressive)	3.99	37.0	Yes			0	3+ HFLU	
	29/09/14	Yes (not new or progressive)	N/A	37.6	Yes			0	0	
	30/09/14	Yes (not new or progressive)	21.24	37.8	Yes			0	0	
	01/10/14	Yes (progressive)	27.08	38.0	Yes		VAP	0	0	
	02/10/14	Yes	22.55	36.4	No			0	0	
	03/10/14	Yes	21.21	36.7	Yes		VAP	0	0	
	04/10/14	Yes	23.24	36.8	Yes	98.2	VAP	0	2+ EC	

	05/10/14	N/A	N/A	N/A	N/A			N/A	N/A	
077	23/05/15	No	11.62	37.9	Yes	100.2		0	0	
	24/05/15	No	13.71	38.3	Yes	100.9		0	0	
	25/05/15	Yes (new)	12.01	37.7	No	99.9		0	2+ ENTC; 3+ HFLU	
	26/05/15	Yes	11.11	37.9	Yes	100.2	VAP	0	3+ ENTC; 4+ HFLU	
	27/05/15	Yes	12.41	38.3	Yes	100.9	VAP	3+	2+ ENTC; 4+ HFLU	
	28/05/15	No	11.53	37.8	Yes	100.1		4+	3+ ENTC	
	29/05/15	No	12.49	37.4	Yes	99.3		4+	3+ ENTC	
	30/05/15	Yes (new)	12.62	38.9	Yes	102	VAP	3+	4+ ENTC	
	31/05/15	Yes	21.12	38.8	Yes	101.8	VAP	0	4+ ENTC	
	01/06/15	Yes	13.91	37.7	N/A	99.8		N/A	N/A	
	02/06/15	No	11.63	37.3	Yes	99.1		0	3+ ENTC	
	03/06/15	No	12.18	37.7	N/A	99.8		N/A	N/A	
	04/06/15	No	12.58	37.3	Yes	99.1		3+	0	
	05/06/15	No	12.1	36.9	N/A	98.4		3+	2+ ENTC	
	06/06/15	No	12	37.5	Yes	99.5		3+	2+ ENTC	
	07/06/15	No	11.88	37.4	N/A	99.3		N/A	N/A	
	08/06/15	No	10.51	38.3	Yes	100.9		1+	1+ ENTC	
	09/06/15	No	12.15	37.8	No	100		4+	4+ ENTC	
	10/06/15	No	9.61	37.5	Yes	99.5		4+	2+ ENTC	
	11/06/15	No	10.36	38.4	Yes	101.1		4+	2+ ENTC	
	12/06/15	No	14.51	37.8	N/A	100.1		N/A	N/A	
078	27/09/14	No	18.88	38.2	Yes			2+	0	
	28/09/14	No	10.75	38.0	Yes			3+	0	
	29/09/14	No	8.35	37.4	N/A	99.4		N/A	N/A	
079	27/09/14	No	2.56	N/A	No			0	4+ KP	
	28/09/14	N/A	N/A	N/A	N/A			N/A	N/A	
080	28/09/14	Yes (not new or progressive)	7.9	37.1	Yes	98.7		3+	2+ KP; 2+ KP (OMOR)	
	29/09/14	Yes (not new or progressive)	8.37	37.2	N/A	98.9		N/A	N/A	
	30/09/14	N/A	N/A	N/A	Yes			N/A	N/A	
081	30/09/14	No	19.17	37.6	Yes			2+	0	
	01/10/14	No	13.42	37.3	Yes			0	0	
	02/10/14	No	12.17	36.8	Yes			0	0	
	03/10/14	No	12.49	38.1	Yes			1+	0	
	04/10/14	No	11.97	37.2	No			1+	0	
	05/10/14	Yes (new)	12.1	37.4	Yes		VAP	1+	0	
	06/10/14	No	11.95	37.5	Yes	99.5		0	0	
	07/10/14	N/A	10.87	37.1	N/A	98.7		N/A	N/A	
082	01/10/14	N/A	34.26	38.9	Yes	102		N/A	N/A	
	02/10/14	Yes (not new or progressive)	22.17	39.6	Yes	103.3		0	0	
	03/10/14	N/A	19.93	37.1	N/A	98.8		N/A	N/A	
	04/10/14	N/A	N/A	N/A	N/A			N/A	N/A	
083	03/10/14	No	13.53	38.1	Yes			0	0	
	04/10/14	Yes (new)	10.54	37.9	N/A			N/A	N/A	
084	03/10/14	Yes (not new or progressive)	25.2	38.0	Yes	100.4		0	0	
	04/10/14	Yes (not new or progressive)	40.5	38.2	Yes	100.8		0	0	
	05/10/14	Yes (not new or progressive)	45.12	37.4	Yes			0	0	
	06/10/14	No	42.43	37.3	Yes			0	0	
	07/10/14	No	37.64	37.8	Yes	100		0	0	
	08/10/14	Yes (new)	31.02	38.2	No	100.8	VAP	0	0	
	09/10/14	Yes	23.13	38.9	Yes	102.1	VAP	0	0	
	10/10/14	No	21.26	37.6	No	99.6		0	0	
085	04/10/14	No	8.35	38.0	N/A	100.4		N/A	N/A	
	05/10/14	No	10.63	37.7	Yes	99.8		0	3+ SERM	
	06/10/14	No	8.05	37.5	Yes	99.5		0	4+ SERM	
	07/10/14	No	6.37	37.3	Yes	99.1		0	4+ SERM	
	08/10/14	No	9.53	38.2	No	100.7		3+	3+ SERM	
086	05/10/14	Yes (not new or progressive)	7.75	35.4	No			0	0	
	06/10/14	Yes (not new or progressive)	10.6	36.4	No			0	0	
	07/10/14	No	25.5	36.7	No			0	0	
	08/10/14	No	28.17	37.4	N/A	99.4		N/A	N/A	
087	08/10/14	Yes (not new or progressive)	13.95	37.6	No	99.7		0	0	
	09/10/14	Yes (progressive)	12.49	37.9	Yes			0	0	
	10/10/14	Yes	8.04	36.7	N/A			N/A	N/A	
088	08/10/14	No	11.63	37.2	N/A	99		N/A	N/A	
	09/10/14	No	8.19	38.1	Yes	100.5		4+	0	
	10/10/14	Yes (new)	8.28	38.6	Yes	101.5	VAP	4+ (MORP2)	0	
	11/10/14	Yes	7.94	39.1	Yes	102.4	VAP	4+ (MORP2)	0	
	12/10/14	Yes	10.31	39.1	Yes	102.3	VAP	4+ (MORP2)	0	
	13/10/14	Yes	9.99	38.8	No	101.8		0	0	
	14/10/14	Yes	9.07	38.3	Yes	101	VAP	3+	0	
	15/10/14	Yes	10.73	38.9	Yes	102.1	VAP	2+	0	
	16/10/14	Yes	7.82	39.7	N/A	103.5		N/A	N/A	
	17/10/14	Yes	8.5	39.0	N/A	102.2		N/A	N/A	
089	09/10/14	Yes (not new or progressive)	6.53	39.1	Yes	102.3		0	0	
	10/10/14	Yes (not new or progressive)	8.62	39.3	Yes	102.8		0	0	
	11/10/14	Yes (not new or progressive)	7.25	39.2	Yes	102.6		0	0	
	12/10/14	Yes (not new or progressive)	6.63	38.7	Yes	101.7		N/A	N/A	
	13/10/14	Yes (not new or progressive)	6.16	39.3	No	102.7		0	0	
	14/10/14	No	5.22	39.1	Yes	102.4		0	0	
	15/10/14	Yes (new)	8.04	38.2	N/A	100.7		0	0	
	16/10/14	Yes	8.61	38.2	Yes	100.8	VAP	N/A	N/A	
	17/10/14	Yes	9	38.8	Yes	101.8	VAP	0	0	
	18/10/14	Yes	8.94	38.1	No	100.5		0	0	
	19/10/14	Yes	9	37.4	Yes	99.4		0	0	
	20/10/14	Yes	7.74	37.4	No	99.4		0	0	
	21/10/14	N/A	N/A	37.3	No	99.2		0	0	
	22/10/14	No	8.45	37.6	No	99.7		0	0	
	23/10/14	No	7.78	37.3	Yes	99.1		0	0	
090	10/10/14	No	11.76	38.9	Yes	102		3+ (MORP2)	0	
	11/10/14	No	20.88	38.5	Yes	101.3		4+ (MORP2)	0	
	12/10/14	Yes (new)	20.59	38.2	Yes	100.8	VAP	4+	0	
	13/10/14	Yes	16.57	38.6	No	101.5	VAP	4+	0	
	14/10/14	Yes	13.07	39.3	Yes	102.7	VAP	4+	0	
	15/10/14	Yes	17.42	37.7	N/A	99.8		N/A	N/A	
	16/10/14	Yes	16.58	37.8	N/A	100.1		N/A	N/A	
	17/10/14	Yes	12.32	38.1	N/A	100.6	VAP	N/A	N/A	
	18/10/14	Yes	12.09	38.0	N/A	100.4		N/A	N/A	
	19/10/14	No	18.67	37.8	Yes	100.1		N/A	N/A	
	20/10/14	No	20.9	37.5	N/A	99.5		N/A	N/A	
	21/10/14	No	15.39	37.3	N/A	99.2		N/A	N/A	
091	10/10/14	No	8.42	38.7	N/A	101.7		0	0	
	11/10/14	N/A	9.95	39.4	Yes	102.9		0	0	
	12/10/14	No	9.7	37.3	Yes	99.2		0	0	
092	10/10/14	No	7.11	36.8	Yes			4+	0	
	11/10/14	No	15.68	37.8	N/A			N/A	N/A	
093	10/10/14	Yes (not new or progressive)	6.23	37.2	No	99		0	0	
	11/10/14	Yes (progressive)	6.69	37.8	Yes	100		0	3+ KOX	
	12/10/14	No	6.08	37.9	Yes	100.2		0	0	
	13/10/14	Yes (new)	7.17	38.9	No	102.1		0	0	
	14/10/14	Yes	9.29	38.1	Yes	100.6	VAP	0	0	
	15/10/14	Yes	9.47	38.1	Yes	100.6	VAP	0	0	

	16/10/14	Yes	8.08	38.2	Yes	100.8	VAP	0	0
	17/10/14	Yes	8.71	37.9	Yes	100.2		0	0
	18/10/14	Yes	8.81	38.1	No	100.6		0	0
	19/10/14	Yes	8.47	38.0	N/A	100.4		N/A	N/A
	20/10/14	Yes	8.35	38.1	No	100.5		0	0
	21/10/14	Yes	10.42	37.9	No	100.3		0	0
	22/10/14	Yes	N/A	38.1	No	100.5		0	0
	23/10/14	Yes	13.61	37.4	Yes	99.4	VAP	0	0
	24/10/14	Yes	12.81	37.9	Yes	100.3	VAP	0	0
	25/10/14	Yes	12.28	38.2	Yes	100.8	VAP	0	0
094	12/10/14	Yes (not new or progressive)	8.59	37.0	Yes			0	0
	13/10/14	Yes (not new or progressive)	8.13	37.9	N/A			N/A	N/A
095	13/10/14	No	16.75	37.0	No	98.6		0	0
	14/10/14	No	5.75	38.2	Yes	100.8		0	0
	15/10/14	No	13.29	37.5	N/A	99.5		N/A	N/A
096	16/10/14	No	14.82	38.3	Yes	101		0	2+ KP
	17/10/14	No	16.41	39.0	Yes	102.2		0	2+ KP
	18/10/14	No	19.78	39.7	Yes	103.5		0	2+ KP
	19/10/14	Yes (new)	20.34	39.7	Yes	103.5	VAP	0	2+ KP
	20/10/14	Yes	17.98	39.4	No	103	VAP	0	3+ KP
	21/10/14	No	17.8	39.6	No			0	2+ KP
	22/10/14	No	21.03	39.2	No			0	2+ KP
	23/10/14	No	23.49	37.6	Yes			0	0
	24/10/14	Yes (new)	15.41	37.9	Yes		VAP	0	0
	25/10/14	Yes	20.84	37.2	Yes		VAP	0	2+ KP
	26/10/14	Yes	24.95	36.8	Yes		VAP	0	2+ KP
	27/10/14	Yes	18.19	36.9	No			0	3+ KP
	28/10/14	Yes	14.09	36.9	Yes		VAP	0	2+ KP
	29/10/14	Yes	9.43	37.3	N/A			N/A	N/A
097	16/10/14	Yes (not new or progressive)	30.54	37.2	No	98.9		0	2+ KP; 1+ EC
	17/10/14	No	25.05	36.0	No			0	0
	18/10/14	No	16.61	37.0	Yes	98.6		0	0
	19/10/14	No	17.11	37.2	Yes	98.9		0	0
	20/10/14	No	21.38	37.2	No	99		0	2+ ACHX
	21/10/14	No	36.79	36.9	No	98.5		0	2+ ACHX
	22/10/14	No	40.63	37.1	Yes	98.7		0	0
098	17/10/14	No	13.61	37.6	Yes	99.6		0	0
	18/10/14	No	12.29	37.4	Yes	99.4		0	0
	19/10/14	No	10.89	37.3	Yes	99.2		0	0
	20/10/14	Yes (new)	10.99	38.1	Yes	100.6	VAP	0	4+ HFLU; 3+ BTSF
	21/10/14	Yes	13.73	37.8	Yes	100	VAP	0	4+ HFLU
	22/10/14	Yes	12.1	38.5	Yes	101.3	VAP	0	4+ HFLU; 4+ BTSF
	23/10/14	No	14.06	38.7	N/A	101.6		N/A	N/A
	24/10/14	No	15.32	37.9	Yes	100.2		0	4+ HFLU; 4+ BTSF
	25/10/14	No	13.36	37.8	Yes	100		0	0
	26/10/14	Yes (new)	11.83	37.3	No	99.1		0	0
	27/10/14	Yes	11.39	38.6	Yes	101.4	VAP	0	0
	28/10/14	Yes	14.6	38.1	Yes	100.5	VAP	0	0
	29/10/14	Yes	11.28	38.1	No	100.5	VAP	0	0
	30/10/14	No	10.45	37.6	Yes	99.6		0	0
099	20/10/14	No	17.54	38.6	N/A	101.4		0	0
	21/10/14	No	18.31	37.6	No	99.6		0	0
	22/10/14	Yes (new)	13.48	37.0	Yes	98.6	VAP	0	0
	23/10/14	Yes	14.66	37.7	N/A	99.9		N/A	N/A
	24/10/14	Yes	10.87	37.6	Yes	99.6		0	0
	25/10/14	Yes	12.12	37.7	Yes	99.8	VAP	0	0
	26/10/14	Yes	14.2	38.7	Yes	101.6	VAP	0	0
	27/10/14	Yes	14.59	38.2	Yes	100.7	VAP	0	0
	28/10/14	Yes	14.35	37.7	No	99.9		0	0
	29/10/14	Yes	12.63	37.8	Yes	100	VAP	0	0
	30/10/14	Yes	13.32	37.7	N/A	99.9		N/A	N/A
100	22/10/14	No	14.73	38.7	N/A	101.6		N/A	N/A
	23/10/14	No	7.01	37.3	Yes	99.1		0	0
101	24/10/14	No	32.03	36.4	N/A	97.6		N/A	N/A
	25/10/14	No	6.58	36.7	Yes	98		0	0
	26/10/14	No	4.3	36.4	Yes	97.5		0	0
	27/10/14	No	4.38	36.7	N/A	98.1		N/A	N/A
	28/10/14	No	10.73	37.2	N/A	98.9		N/A	N/A
	29/10/14	N/A	6.4	36.7	N/A	98		N/A	N/A
102	24/10/14	Yes (not new or progressive)	24.47	37.2	N/A	99		N/A	N/A
	25/10/14	Yes (not new or progressive)	27.22	37.6	No	99.6		0	1+ ENTC
	26/10/14	Yes (not new or progressive)	17.73	37.4	No	99.4		0	0
	27/10/14	Yes (not new or progressive)	12.24	37.7	N/A	99.9		N/A	N/A
	28/10/14	Yes (not new or progressive)	11.95	37.6	Yes	99.6		0	0
	29/10/14	Yes (not new or progressive)	14.33	37.7	No	99.9		0	2+ ENTC
	30/10/14	No	14.65	37.5	Yes	99.5		0	0
103	25/10/14	No	7.69	36.6	Yes			3+	3+ EC
	26/10/14	N/A	9.68	36.4	N/A	97.6		N/A	N/A
104	25/10/14	Yes (not new or progressive)	16	37.7	No	99.8		0	0
	26/10/14	Yes (not new or progressive)	18.59	36.8	No	98.3		0	0
	27/10/14	Yes (not new or progressive)	16.63	37.1	Yes	98.8		0	0
	28/10/14	Yes (not new or progressive)	12.35	37.2	Yes	98.9		0	0
	29/10/14	Yes (progressive)	13.95	37.6	No	99.6		0	3+ PSA
	30/10/14	No	16.15	37.7	Yes	99.8		0	0
	31/10/14	Yes (new)	18.61	37.3	Yes	99.2	VAP	0	0
	01/11/14	No	15.41	37.3	Yes	99.1		0	0
	02/11/14	Yes (new)	15.6	38.2	Yes	100.7	VAP	0	0
	03/11/14	Yes	14.87	38.6	No	101.5	VAP	0	0
	04/11/14	Yes	22.43	38.8	No	101.9	VAP	0	0
	05/11/14	Yes	13.86	39.3	No	102.8	VAP	0	0
	06/11/14	Yes	11.37	39.6	Yes	103.2	VAP	0	0
	07/11/14	Yes	20.44	38.1	Yes		VAP	0	0
	08/11/14	No	20.48	37.9	N/A			N/A	N/A
	09/11/14	Yes (new)	19.06	37.9	N/A			N/A	N/A
	10/11/14	Yes	18.26	38.4	Yes		VAP	N/A	N/A
	11/11/14	Yes	23.6	38.4	Yes		VAP	0	0
	12/11/14	Yes	25.62	38.3	N/A		VAP	N/A	N/A
	13/11/14	N/A	N/A	N/A	N/A			N/A	N/A
105	26/10/14	Yes (not new or progressive)	39.45	37.8	Yes	100.1		N/A	N/A
	27/10/14	Yes (not new or progressive)	40.84	37.7	N/A	99.8		N/A	N/A
	28/10/14	Yes (not new or progressive)	45.43	37.2	N/A	99		N/A	N/A
	29/10/14	Yes (not new or progressive)	56.2	37.7	No	99.8		2+	0
	30/10/14	Yes (not new or progressive)	55.2	37.6	No	99.6		3+	0
	31/10/14	No	44.16	37.6	Yes	99.6		1+	0
106	27/10/14	Yes (not new or progressive)	27.43	39.0	Yes	102.2		0	2+ PSA
	28/10/14	Yes (not new or progressive)	23.63	36.6	N/A	97.8		0	3+ PSA
	29/10/14	Yes (not new or progressive)	16.53	37.0	N/A	98.6		0	3+ PSA
	30/10/14	No	19.88	37.2	Yes	99		0	3+ PSA
	31/10/14	No	23.47	36.8	Yes	98.2		0	3+ PSA
	01/11/14	Yes (new)	26.13	37.8	Yes	100	VAP	0	3+ PSA
	02/11/14	Yes	20.91	37.3	Yes	99.2	VAP	0	3+ PSA
	03/11/14	No	10.36	37.1	N/A	98.7		N/A	N/A
107	28/10/14	No	6.91	38.0	Yes	100.4		3+	3+ ENTA

	29/10/14	No	7.82	37.9	No	100.3		4+	1+ ENTA	
	30/10/14	No	8.74	38.0	Yes	100.4		4+	2+ ENTA	
	31/10/14	No	7.71	38.0	Yes	100.4		1+	0	
	01/11/14	Yes (new)	8.26	38.6	No	101.4		0	3+ SERM	
	02/11/14	Yes	9.66	38.4	Yes	101.2	VAP	0	2+ SERM	
	03/11/14	Yes	11.07	38.1	No	100.5	VAP	0	3+ SERM; 2+ ENTA	
	04/11/14	No	11.05	38.1	Yes	100.5		0	3+ SERM	
	05/11/14	Yes (new)	9.93	37.9	Yes	100.2		0	3+ SERM	
	06/11/14	No	9.4	37.9	No	100.2		0	2+ SERM; 1+ ENTA	
	07/11/14	No	12.85	38.1	N/A	100.5		N/A	N/A	
108	28/10/14	No	13.86	38.5	Yes	101.3		0	0	
	29/10/14	No	N/A	37.6	No	99.6		0	0	
	30/10/14	No	11.3	37.7	Yes	99.8		0	0	
	31/10/14	No	15.85	37.6	Yes	99.7		0	0	
	01/11/14	No	12.86	37.6	No	99.6		0	0	
	02/11/14	N/A	8.94	37.4	N/A	99.3		N/A	N/A	
109	23/05/15	Yes (not new or progressive)	13.16	38.3	No	100.9		1+	0	
	24/05/15	Yes (not new or progressive)	3.58	38.3	No	101		N/A	N/A	
	25/05/15	Yes (not new or progressive)	5.89	37.7	N/A	99.8		N/A	N/A	
	26/05/15	Yes (not new or progressive)	5.63	39.1	N/A	102.3		N/A	N/A	
	27/05/15	Yes (not new or progressive)	7.99	38.0	No	100.4		1+	0	
	28/05/15	Yes (not new or progressive)	3.93	39.3	N/A	102.7		N/A	N/A	
	29/05/15	Yes (not new or progressive)	4.83	38.2	No	100.7		1+	0	
	30/05/15	Yes (not new or progressive)	4.86	37.6	No	99.7		2+	3+ XMAL	
	31/05/15	Yes (not new or progressive)	N/A	N/A	N/A			N/A	N/A	
110	24/05/15	No	14.16	37.5	N/A	99.5		N/A	N/A	
	25/05/15	Yes (new)	8.51	36.9	Yes	98.4		0	0	
	26/05/15	Yes (progressive)	8.15	37.6	Yes	99.6		0	0	
	27/05/15	Yes	11.54	37.3	N/A	99.1		N/A	N/A	
	28/05/15	Yes	9.48	37.7	No	99.9		0	0	
	29/05/15	No	10.21	37.6	Yes	99.7		0	0	
	30/05/15	No	6.34	37.6	No	99.6		0	0	
	31/05/15	No	6.94	37.3	Yes	99.1		0	0	
	01/06/15	No	7.49	37.2	Yes	99		0	2+ ENTC	
	02/06/15	No	8.56	36.9	N/A	98.4		N/A	N/A	
111	01/11/14	Yes (not new or progressive)	14.97	37.7	No	99.8		0	0	
	02/11/14	Yes (not new or progressive)	11.41	38.1	No	100.5		0	0	
	03/11/14	No	12.67	38.1	No	100.5		0	0	
	04/11/14	Yes (new)	14.39	38.3	Yes	101	VAP	0	0	
	05/11/14	Yes	14.62	37.5	No	99.5		0	0	
	06/11/14	Yes	12.8	37.1	N/A	98.7		N/A	N/A	
112	01/11/14	Yes (not new or progressive)	20.54	36.9	No	98.5		N/A	N/A	
	02/11/14	Yes (progressive)	18.6	37.7	Yes	99.8		0	0	
	03/11/14	No	18.83	37.4	Yes	99.3		1+	0	
	04/11/14	Yes (new)	8.6	37.5	Yes	99.5		0	0	
	05/11/14	No	11.82	37.2	N/A	98.9		N/A	N/A	
113	25/05/15	Yes (not new or progressive)	14.63	38.1	No	100.6		0	0	
	26/05/15	No	12.06	38.1	No	100.6		0	0	
	27/05/15	Yes (new)	14.92	37.3	No	99.1		N/A	N/A	
	28/05/15	Yes	7.58	38.1	No	100.5		0	0	
	29/05/15	Yes	6.78	38.9	No	102		0	0	
	30/05/15	Yes	7.47	39.1	No	102.4		0	0	
	31/05/15	No	9.97	37.8	No	100.1		0	0	
	01/06/15	N/A	10.05	38.2	No	100.7		0	0	
	02/06/15	Yes (new)	9.1	37.9	Yes	100.2		0	0	
	03/06/15	No	10.64	37.4	N/A	99.3		N/A	N/A	
	04/06/15	No	12.16	36.7	N/A	98		N/A	N/A	
114	04/11/14	No	13.26	38.1	Yes	100.6		0	0	
	05/11/14	No	14.17	38.6	Yes			0	0	
	06/11/14	No	12.09	38.6	No			0	0	
	07/11/14	N/A	N/A	37.8	Yes			0	0	
	08/11/14	N/A	N/A	N/A	N/A			N/A	N/A	
115	06/11/14	No	26.17	37.9	Yes	100.2		N/A	N/A	
	07/11/14	No	14.41	37.2	Yes	98.9		0	0	
	08/11/14	N/A	N/A	N/A	N/A			N/A	N/A	
116	06/11/14	No	11.22	33.8	N/A			N/A	N/A	
	07/11/14	No	9.38	38.1	Yes	100.6		0	0	
	08/11/14	No	9.72	38.0	No			1+	0	
	09/11/14	No	12.66	37.9	No			0	0	
	10/11/14	No	13.84	37.4	No			0	0	
	11/11/14	Yes (new)	19.53	38.6	Yes		VAP	0	0	
	12/11/14	Yes	15.09	37.9	N/A			N/A	N/A	
	13/11/14	Yes	17.37	37.6	No	99.7		0	0	
	14/11/14	No	25.47	38.3	Yes			0	2+ ACHX	
	15/11/14	Yes (new)	37.28	38.2	Yes		VAP	N/A	N/A	
	16/11/14	Yes	34.95	36.7	Yes		VAP	0	3+ ACHX	
	17/11/14	Yes	28.84	36.7	Yes	98	VAP	0	4+ ACHX	
	18/11/14	No	23.49	36.9	Yes	98.4		0	4+ ACHX	
	19/11/14	N/A	N/A	N/A	N/A			N/A	N/A	
117	09/11/14	Yes (not new or progressive)	18.72	37.1	Yes	98.8		0	0	
	10/11/14	Yes (progressive)	14.05	38.4	Yes	101.1		0	0	
	11/11/14	N/A	10.67	36.9	N/A	98.4		N/A	N/A	
118	10/11/14	No	19.66	36.9	Yes	98.5		0	3+ KP; 4+ DIPS	
	11/11/14	No	13.02	36.7	Yes	98		0	3+ KP; 3+ DIPS	
	12/11/14	No	16.08	36.8	No	98.2		0	4+ KP; 4+ DIPS	
	13/11/14	No	14.24	37.6	Yes	99.7		0	3+ KP; 2+ DIPS	KP
	14/11/14	No	12.14	37.6	Yes			0	3+ KP	
	15/11/14	Yes (new)	13.4	38.0	Yes	100.4	VAP	0	4+ KP	
119	27/05/15	No	10.42	37.5	Yes	99.5		0	0	
	28/05/15	Yes (new)	7.93	37.3	N/A	99.2		N/A	N/A	
120	12/11/14	No	14.53	38.0	N/A	100.4		N/A	N/A	
	13/11/14	No	10.7	37.4	N/A	99.4		0	0	
	14/11/14	No	8.62	36.8	N/A	98.3		N/A	N/A	
121	12/11/14	Yes (not new or progressive)	12.54	37.7	No	99.9		0	0	
	13/11/14	Yes (not new or progressive)	10.72	38.1	No	100.5		0	0	
	14/11/14	Yes (not new or progressive)	17.42	38.2	No	100.8		0	3+ SPNE	
	15/11/14	Yes (not new or progressive)	13.8	39.1	No	102.3		0	3+ SPNE	
	16/11/14	Yes (not new or progressive)	13.93	38.8	No	101.8		0	0	
	17/11/14	Yes (progressive)	16.61	38.1	No	100.6	VAP	0	0	
	18/11/14	Yes	13.07	39.2	No	102.6	VAP	0	0	
	19/11/14	Yes	13.39	39.2	Yes	102.6	VAP	0	1+ DIPS	
	20/11/14	No	13.77	38.4	No	101.2		0	0	
	21/11/14	No	16.07	38.1	N/A	100.6		N/A	N/A	
	22/11/14	No	20.24	37.9	N/A	100.2		N/A	N/A	
122	27/05/15	Yes (not new or progressive)	27.24	37.6	No	99.7		0	0	
	28/05/15	N/A	16.22	37.3	N/A	99.2		N/A	N/A	
123	13/11/14	No	53.14	38.6	No	101.5		0	3+ KP; 3+ PSA	
	14/11/14	Yes (new)	42.09	37.4	Yes			3+	3+ PSA	
	15/11/14	Yes	19.62	37.0	No	98.6		0	2+ KP; 2+ PSA	
	16/11/14	Yes	13.87	37.8	No	100		0	2+ KP; 2+ PSA	
	17/11/14	Yes	16.31	37.8	No	100		0	2+ KP; 2+ PSA	
	18/11/14	Yes	17.49	39.2	Yes	102.5		3+	3+ PSA; 3+ SERM	
	19/11/14	Yes	15.01	37.8	Yes	100.1		1+	2+ DIPS; 2+ PSA	

	20/11/14	Yes	14.01	37.4	Yes	99.3		0	2+ PSA; 2+ SERM	
	21/11/14	Yes	13.8	37.2	No	98.9		0	3+ PSA; 2+ SERM	
	22/11/14	Yes	12.35	37.1	Yes	98.7		0	3+ PSA; 2+ SERM	
124	13/11/14	No	14.58	37.2	Yes	98.9		0	4+ KOX	
	14/11/14	No	9.88	38.2	Yes	100.7		0	3+ KOX	
	15/11/14	Yes (new)	9.01	38.8	Yes	101.9	VAP	0	3+ KOX	
	16/11/14	Yes	10.47	39.0	Yes	102.2	VAP	0	2+ KOX; 3+ MOST-MORX	
	17/11/14	Yes	12.61	38.6	Yes	101.5	VAP	0	2+ KOX; 1+ MORX	
	18/11/14	Yes	12.59	38.9	Yes	102	VAP	0	1+ KOX	
	19/11/14	Yes	10.12	38.3	No	101		0	1+ KOX	
	20/11/14	Yes	10.95	39.3	Yes	102.8	VAP	0	2+ KOX	
	21/11/14	No	10.65	38.6	Yes	101.4		0	2+ KOX	
	22/11/14	Yes (new)	12.92	38.2	No	100.8	VAP	0	2+ KOX	
	23/11/14	No	15.25	38.2	No	100.7		0	1+ KOX	
	24/11/14	No	15.63	37.7	N/A	99.9		0	2+ KOX	
125	14/11/14	Yes (not new or progressive)	18.84	37.2	Yes	99		3+	0	
	15/11/14	Yes (not new or progressive)	23.38	37.7	Yes	99.9		3+	0	SAUR
	16/11/14	Yes (not new or progressive)	11.61	37.4	Yes	99.3		3+	0	
	17/11/14	Yes (progressive)	8.33	36.9	Yes	98.5		2+	0	
	18/11/14	No	7.33	37.1	Yes	98.8		3+	0	
	19/11/14	Yes (new)	9.33	37.6	Yes	99.6		2+	0	
	20/11/14	No	10.47	37.3	Yes	99.1		2+	0	
	21/11/14	Yes (new)	7.16	37.3	Yes	99.2		2+ (MORP2)	0	
	22/11/14	Yes	6.32	37.0	Yes	98.6		2+; 2+ (OMOR)	0	
	23/11/14	Yes	7.42	37.1	No	98.8		2+; 2+ (OMOR)	0	
	24/11/14	Yes	6.02	36.8	Yes	98.2		2+ (MORP2)	0	
126	14/11/14	No	15.76	38.3	Yes	101		0	0	Yeast
	15/11/14	Yes (new)	15.38	37.3	Yes	99.1		0	0	
	16/11/14	Yes	16.17	37.6	No	99.6		0	0	
	17/11/14	Yes	18.51	36.4	No	97.6		0	0	
	18/11/14	Yes	25.73	36.8	Yes	98.3		N/A	N/A	
	19/11/14	N/A	N/A	N/A	N/A			N/A	N/A	
127	15/11/14	Yes (not new or progressive)	19.32	38.3	Yes	101		0	3+ ENTC	
	16/11/14	Yes (not new or progressive)	14.01	37.5	Yes	99.5		0	3+ ENTC	
128	16/11/14	Yes (not new or progressive)	2.92	34.4	N/A			N/A	N/A	
	17/11/14	No	5.42	37.9	No			0	0	
	18/11/14	Yes (new)	4.63	36.9	Yes			0	0	
	19/11/14	Yes	7.74	37.8	Yes			0	0	
	20/11/14	Yes	9.79	38.3	Yes		VAP	0	0	
	21/11/14	No	10.78	39.2	N/A			N/A	N/A	
129	17/11/14	No	7.35	37.1	No	98.8		0	0	
	18/11/14	Yes (new)	10.79	37.0	Yes	98.6		0	4+ HFLU	
	19/11/14	Yes	11.3	37.1	Yes	98.8		0	4+ HFLU	
	20/11/14	Yes	11.42	37.3	N/A	99.1		N/A	N/A	
	21/11/14	No	7.54	37.2	No	98.9		0	0	
	22/11/14	Yes (new)	9.9	37.1	Yes	98.7		0	0	
	23/11/14	Yes	11.01	36.9	No	98.5		0	0	
	24/11/14	No	7.16	37.0	No	98.6		0	0	
	25/11/14	Yes (new)	8.64	36.9	No	98.5		0	2+ HFLU	
	26/11/14	Yes	12.15	37.0	Yes	98.6	VAP	0	3+ HFLU	
	27/11/14	Yes	15.51	37.1	N/A	98.7		N/A	N/A	
130	18/11/14	Yes (not new or progressive)	13.77	36.9	Yes	98.5		0	0	
	19/11/14	Yes (progressive)	22.05	36.9	Yes	98.4	VAP	0	0	
	20/11/14	Yes	17.67	37.3	No	99.2		0	0	
	21/11/14	Yes (progressive)	18.43	37.4	No	99.3		0	0	
	22/11/14	Yes	24.6	38.1	Yes	100.5	VAP	0	0	
	23/11/14	No	25.52	36.9	Yes	98.4		0	0	
	24/11/14	No	22.81	37.2	No	99		0	0	
	25/11/14	Yes (new)	21.52	38.6	No	101.4	VAP	0	0	
	26/11/14	Yes	18.89	37.9	Yes	100.2	VAP	0	0	
	27/11/14	No	20.4	37.5	N/A	99.5		N/A	N/A	
131	20/11/14	Yes (not new or progressive)	16.11	36.9	No	98.5		0	0	
	21/11/14	No	23.52	37.3	Yes	99.2		0	0	
	22/11/14	No	32.36	37.0	No	98.6		0	0	
	23/11/14	No	36.92	37.3	Yes	99.2		0	0	
	24/11/14	No	45.61	37.4	Yes	99.3		0	0	
	25/11/14	Yes (new)	40.32	37.4	Yes	99.3	VAP	0	0	
	26/11/14	No	31.73	37.2	N/A	98.9		N/A	N/A	
132	22/11/14	Yes (not new or progressive)	6.08	38.3	No			0	0	
	23/11/14	Yes (not new or progressive)	10.56	37.6	No			0	0	
	24/11/14	Yes (not new or progressive)	9.12	37.1	Yes	98.7		0	0	
	25/11/14	Yes (not new or progressive)	8.11	36.7	No	98		0	0	
	26/11/14	Yes (not new or progressive)	10.65	37.7	N/A	99.9		N/A	N/A	
133	24/11/14	Yes (not new or progressive)	8.06	38.9	N/A			N/A	N/A	
	25/11/14	N/A	7.1	38.7	N/A	101.7		N/A	N/A	
	26/11/14	Yes (not new or progressive)	7.65	38.7	Yes	101.7		N/A	N/A	
	27/11/14	Yes (not new or progressive)	5.38	38.7	N/A			N/A	N/A	
	28/11/14	No	6.1	38.5	N/A			N/A	N/A	
	29/11/14	Yes (new)	7.15	38.8	Yes	101.9	VAP	1+	0	
	30/11/14	No	9.15	38.2	Yes			2+	0	
	01/12/14	No	9.64	37.4	N/A	99.4		N/A	N/A	
	02/12/14	No	10.94	37.4	No	99.3		0	0	
	03/12/14	No	13.7	37.1	N/A	98.7		N/A	N/A	
134	28/05/15	No	12.48	37.5	No	99.5		2+	0	
	29/05/15	No	10.19	37.0	N/A	98.6		N/A	N/A	
	30/05/15	Yes (new)	7.42	37.2	Yes	99		4+	0	
	31/05/15	Yes	8.83	37.6	Yes	99.7		4+	0	
	01/06/15	No	13.15	38.0	Yes	100.4		4+	0	
	02/06/15	Yes (new)	15.3	37.3	Yes	99.1	VAP	4+	0	
	03/06/15	No	11.59	37.8	Yes	100.1		3+	0	
	04/06/15	No	18.61	37.4	Yes	99.3		2+	0	
	05/06/15	Yes (new)	19.11	37.4	Yes	99.3	VAP	3+	0	
	06/06/15	Yes	21.67	37.4	Yes	99.4	VAP	2+	0	
	07/06/15	Yes	22.48	37.2	Yes	98.9	VAP	2+	0	
	08/06/15	N/A	25.26	37.1	Yes	98.7		1+	0	
	09/06/15	No	25.92	36.9	No	98.5		N/A	N/A	
	10/06/15	No	26.86	37.2	Yes	99		1+	0	
	11/06/15	No	19.69	36.9	Yes	98.5		0	0	
	12/06/15	No	18.51	37.6	No	99.6		0	0	
	13/06/15	No	17.71	37.1	Yes	98.8		0	0	
	14/06/15	N/A	N/A	N/A	N/A			N/A	N/A	
135	28/05/15	N/A	15.84	37.3	Yes	99.1		2+	0	
	29/05/15	No	10.04	37.1	N/A	98.7		N/A	N/A	
136	30/05/15	N/A	9.02	37.1	Yes	98.8		3+	0	
	31/05/15	No	7.87	36.8	N/A	98.2		N/A	N/A	
137	26/11/14	Yes (not new or progressive)	11.93	37.3	No	99.1		N/A	N/A	
	27/11/14	No	10.1	38.4	N/A	101.1		N/A	N/A	
	28/11/14	No	10	38.1	Yes	100.5		0	0	
	29/11/14	No	9.19	37.7	Yes	99.9		0	0	
	30/11/14	No	7.27	36.8	Yes	98.3		1+	0	
	01/12/14	N/A	N/A	36.6	N/A	97.9		N/A	N/A	
138	28/11/14	No	5.77	37.8	Yes			1+	0	

	29/11/14	No	5	37.7	Yes			0	0	
	30/11/14	N/A	4.33	N/A	N/A			N/A	N/A	
139	18/06/15	No	16.31	38.1	No	100.5		0	0	
	19/06/15	No	16.01	38.6	Yes	101.5		0	0	
	20/06/15	No	15.59	38.4	No	101.1		0	0	
	21/06/15	No	15.45	38.0	Yes	100.4		0	0	
	22/06/15	No	17.36	38.2	No	100.7		0	0	
	23/06/15	No	17.2	38.7	Yes	101.6		0	0	
	24/06/15	No	16.33	38.1	N/A	100.6		N/A	N/A	
	25/06/15	N/A	N/A	N/A	N/A			N/A	N/A	
140	28/11/14	Yes (not new or progressive)	20.26	36.9	Yes	98.4		3+	0	
	29/11/14	Yes (not new or progressive)	21.85	37.0	Yes	98.6		3+	0	
	30/11/14	Yes (not new or progressive)	21.38	37.1	Yes	98.8		2+	0	
	01/12/14	N/A	23.35	37.5	Yes	99.5		3+	0	
	02/12/14	Yes (not new or progressive)	24.32	37.9	No	100.3		3+	0	
	03/12/14	Yes (not new or progressive)	23.18	37.4	Yes	99.3		2+	0	
	04/12/14	Yes (not new or progressive)	19.48	37.0	No	98.6		3+	0	
	05/12/14	Yes (not new or progressive)	25.83	37.8	Yes	100.1		3+	0	
	06/12/14	Yes (not new or progressive)	19.92	38.1	Yes	100.5		4+	0	
	07/12/14	Yes (not new or progressive)	20.54	38.4	Yes	101.1		3+	0	
	08/12/14	Yes (not new or progressive)	19.96	37.3	Yes	99.2		2+	0	
	09/12/14	Yes (not new or progressive)	25.79	38.1	Yes	100.6		2+	0	
	10/12/14	N/A	N/A	37.4	Yes	99.4		3+	0	
	11/12/14	N/A	N/A	37.1	Yes	98.8		2+	0	
141	28/11/14	Yes (not new or progressive)	11.69	37.3	Yes	99.2		3+	0	
	29/11/14	Yes (not new or progressive)	12.85	36.5	Yes	97.7		2+	0	
	30/11/14	Yes (not new or progressive)	11.68	37.2	Yes	98.9		1+	2+ DIPS	
	01/12/14	Yes (not new or progressive)	11.74	37.2	Yes	98.9		1+	0	
	02/12/14	Yes (not new or progressive)	9.41	37.0	No	98.6		2+	0	
	03/12/14	No	10.69	37.7	Yes	99.9		0	0	
	04/12/14	No	8.8	37.9	Yes	100.2		0	0	
	05/12/14	N/A	N/A	N/A	N/A			N/A	N/A	
142	29/11/14	Yes (not new or progressive)	8.8	37.1	Yes	98.8		0	2+ XMAL	
	30/11/14	Yes (not new or progressive)	6.55	37.1	Yes	98.7		0	3+ XMAL	
	01/12/14	N/A	5.6	N/A	N/A			N/A	N/A	
143	29/11/14	No	4.86	37.3	No	99.2		0	0	
	30/11/14	No	4.15	37.4	Yes	99.4		0	0	
	01/12/14	No	5.69	37.0	No	98.6		0	0	
	02/12/14	No	5.31	36.9	Yes	98.5		0	0	
	03/12/14	No	4.16	38.1	Yes	100.5		0	0	
	04/12/14	No	4.62	37.7	Yes	99.9		0	0	
	05/12/14	No	6.68	37.3	N/A	99.2		N/A	N/A	
144	30/11/14	No	6.7	37.8	No	100		1+	0	
	01/12/14	N/A	5.28	37.1	N/A	98.7		N/A	N/A	
	02/12/14	N/A	10.4	37.3	N/A	99.2		N/A	N/A	
145	30/11/14	No	16.41	28.8	No			2+	N/A	
	01/12/14	No	13.98	32.5	N/A			N/A	N/A	
	02/12/14	No	23.33	36.1	No			1+	0	
	03/12/14	No	30.87	38.3	Yes			N/A	N/A	
	04/12/14	Yes (new)	32.24	36.1	Yes		VAP	0	0	
	05/12/14	Yes	21.84	35.7	Yes		VAP	0	0	
	06/12/14	No	19.94	35.3	Yes			0	0	
	07/12/14	No	21.3	36.9	Yes			0	0	
	08/12/14	Yes (new)	29.77	37.7	Yes	99.8	VAP	0	0	
	09/12/14	No	27.89	37.4	N/A	99.4		N/A	N/A	
	10/12/14	No	22.67	37.1	Yes	98.8		0	0	
	11/12/14	Yes (new)	16.2	38.1	Yes	100.5	VAP	0	0	
	12/12/14	No	15.91	38.1	N/A	100.5		N/A	N/A	
	13/12/14	No	20.23	38.4	N/A	101.2		N/A	N/A	
	14/12/14	N/A	19.27	38.6	Yes	101.5		0	0	
	15/12/14	No	19.38	38.6	No	101.4		0	4+ KP	
	16/12/14	No	18.54	37.8	Yes	100		0	4+ KP	
	17/12/14	No	18.12	37.8	Yes	100.1		0	3+ KP	
	18/12/14	No	17.64	38.2	Yes	100.8		0	3+ KP	
	19/12/14	No	22.39	38.4	Yes	101.2		0	3+ KP	
	20/12/14	N/A	N/A	N/A	N/A			N/A	N/A	
146	30/11/14	No	12.87	37.7	N/A	99.8		N/A	N/A	
	01/12/14	Yes (new)	10.21	37.9	Yes	100.3		3+	0	
	02/12/14	No	12.48	38.1	Yes	100.6		4+	0	
	03/12/14	No	11.28	38.2	Yes	100.7		4+	0	
	04/12/14	No	9.29	38.1	N/A	100.6		N/A	N/A	
	05/12/14	No	11.9	39.3	Yes	102.7		4+	0	
	06/12/14	No	10.48	37.9	Yes	100.2		3+	0	
	07/12/14	Yes (new)	10.19	38.2	Yes	100.7	VAP	4+	2+ KP	
	08/12/14	Yes	N/A	37.7	Yes	99.8		3+	0	
	09/12/14	Yes	N/A	38.1	Yes	100.5	VAP	3+	0	
	10/12/14	N/A	12.96	38.2	Yes	100.8		3+	0	
	11/12/14	Yes	14.26	37.9	Yes	100.2	VAP	4+	0	
	12/12/14	N/A	N/A	37.7	Yes	99.9		3+	0	
147	30/11/14	No	10.35	36.9	N/A	98.5		N/A	N/A	
	01/12/14	No	8.86	37.0	No	98.6		0	0	
	02/12/14	No	7.48	37.6	Yes	99.6		0	0	
	03/12/14	No	7.75	37.1	N/A	98.8		N/A	N/A	
148	01/12/14	No	4.94	37.5	Yes	99.5		0	0	
	02/12/14	No	4.49	37.7	Yes	99.9		0	0	
	03/12/14	Yes (new)	4.79	38.1	Yes	100.5	VAP	2+	4+ PSA	
	04/12/14	Yes	6.29	36.9	Yes	98.5		0	4+ PSA	
	05/12/14	Yes	6.47	36.9	Yes	98.5		0	3+ PSA	
	06/12/14	Yes	5.34	36.6	Yes	97.9		0	4+ PSA	
	07/12/14	Yes	8.49	36.6	Yes	97.9		0	4+ PSA	
	08/12/14	Yes	10.02	37.2	Yes	99		0	3+ PSA	
	09/12/14	Yes	5.65	36.8	No	98.2		0	4+ PSA	
	10/12/14	Yes	7.92	36.9	Yes	98.4		0	3+ PSA	
	11/12/14	Yes	7.97	37.3	Yes	99.1		0	4+ PSA	
	12/12/14	No	6.42	34.4	N/A	93.9		N/A	N/A	
149	05/12/14	No	12.68	37.6	Yes	99.7		0	0	
	06/12/14	No	13.12	37.8	Yes	100		0	0	
	07/12/14	No	12.13	37.5	Yes	99.5		0	0	
	08/12/14	No	12.55	37.8	Yes	100.1		0	0	
	09/12/14	No	9.05	37.3	No	99.1		0	0	
	10/12/14	Yes (new)	10.55	37.7	Yes	99.8		0	0	
	11/12/14	No	11	37.2	Yes	99		0	0	
	12/12/14	No	12.45	37.5	Yes	99.5		0	0	
	13/12/14	No	14.15	37.7	No	99.9		0	0	
	14/12/14	No	16.45	37.4	N/A	99.4		N/A	N/A	
150	03/12/14	Yes (not new or progressive)	7.43	37.1	No	98.7		0	0	
	04/12/14	Yes (not new or progressive)	5.5	37.3	No	99.2		0	0	
	05/12/14	Yes (not new or progressive)	4.27	37.4	No	99.3		0	0	
	06/12/14	Yes (not new or progressive)	4.61	37.7	Yes	99.8		0	0	
	07/12/14	Yes (not new or progressive)	3.29	37.6	No	99.6		0	0	
	08/12/14	Yes (not new or progressive)	3.01	37.0	No	98.6		0	0	
	09/12/14	Yes (not new or progressive)	3.06	37.6	Yes	99.6		0	0	
	10/12/14	No	2.8	37.5	No	99.5		0	0	

	11/12/14	No	3.1	37.9	Yes	100.2		0	0	
	12/12/14	Yes (new)	3.67	37.9	N/A	100.3		N/A	N/A	
151	06/12/14	Yes (not new or progressive)	14.8	36.9	N/A	98.4		N/A	N/A	
	07/12/14	Yes (not new or progressive)	12.41	37.3	Yes	99.2		3+	0	
	08/12/14	Yes (not new or progressive)	8.69	36.9	Yes	98.5		2+	0	
	09/12/14	Yes (progressive)	3.85	36.8	N/A	98.2		N/A	N/A	
152	03/12/14	No	12.06	37.8	N/A			N/A	N/A	
	04/12/14	No	9.1	38.3	Yes			2+	0	
	05/12/14	No	8.32	38.0	Yes			1+	1+ BTSB	
	06/12/14	No	8.33	37.6	N/A			N/A	N/A	
	07/12/14	Yes (new)	8.49	38.0	Yes			0	2+ BTSB	
	08/12/14	Yes	8.41	38.5	Yes		VAP	N/A	N/A	
	09/12/14	Yes	10	38.3	Yes		VAP	0	0	
	10/12/14	Yes	11.31	38.3	N/A	100.9	VAP	N/A	N/A	
153	07/12/14	Yes (not new or progressive)	7.81	37.3	N/A	99.1		N/A	N/A	
	08/12/14	Yes (not new or progressive)	8.44	37.2	Yes	99		0	0	
	09/12/14	No	7.96	37.0	N/A	98.6		N/A	N/A	
154	02/06/15	No	8.89	37.1	N/A	98.8		N/A	N/A	
	03/06/15	No	10.15	37.0	N/A	98.6		N/A	N/A	
	04/06/15	No	8.56	36.9	N/A	98.5		N/A	N/A	
155	07/12/14	Yes (not new or progressive)	4.71	38.7	N/A	101.7		N/A	N/A	
	08/12/14	Yes (not new or progressive)	1.71	37.7	No	99.9		0	0	
	09/12/14	Yes (not new or progressive)	1.01	37.4	No	99.3		0	0	
	10/12/14	Yes (progressive)	1.12	37.3	No	99.1		0	0	
	11/12/14	No	3.9	37.6	No	99.6		0	0	
	12/12/14	Yes (new)	10.1	38.4	Yes	101.2	VAP	0	0	
	13/12/14	Yes	17.38	38.3	No	101	VAP	0	3+ XMAL	
	14/12/14	Yes	26.5	38.4	Yes	101.1	VAP	0	2+ XMAL	
	15/12/14	Yes	36.9	38.4	N/A	101.1	VAP	N/A	N/A	XMAL
	16/12/14	Yes	51.7	N/A	N/A			N/A	N/A	
156	08/12/14	Yes (not new or progressive)	9.08	38.8	N/A	101.9		N/A	N/A	
	09/12/14	Yes (not new or progressive)	16.58	37.4	Yes	99.4		0	0	
	10/12/14	Yes (not new or progressive)	16.77	39.3	Yes	102.7		0	0	
	11/12/14	Yes (not new or progressive)	18.5	39.4	Yes	103		0	0	
	12/12/14	Yes (not new or progressive)	19.06	37.1	N/A	98.7		N/A	N/A	
	13/12/14	Yes (not new or progressive)	12.8	39.9	N/A	103.9		N/A	N/A	
	14/12/14	Yes (not new or progressive)	N/A	39.3	No	102.8		0	0	
157	10/12/14	Yes (not new or progressive)	20.13	37.8	No	100		N/A	N/A	
	11/12/14	Yes (not new or progressive)	24.4	37.3	Yes	99.1		0	0	
	12/12/14	Yes (not new or progressive)	12.64	36.9	N/A	98.4		N/A	N/A	
158	03/06/15	Yes (not new or progressive)	17.67	37.3	Yes	99.1		0	0	
	04/06/15	Yes (not new or progressive)	14.59	36.9	N/A	98.5		N/A	N/A	
	05/06/15	Yes (progressive)	11.42	37.3	No	99.1		0	0	
	06/06/15	Yes	11.94	37.0	Yes	98.6	VAP	0	0	
	07/06/15	Yes	12.03	37.1	Yes	98.7	VAP	0	0	
	08/06/15	No	12.29	37.2	Yes	98.9		0	0	
	09/06/15	No	12.06	37.2	No	99		N/A	N/A	
	10/06/15	No	10.87	37.3	No	99.2		0	0	
	11/06/15	Yes (new)	8.89	37.0	No	98.6		0	0	
	12/06/15	N/A	N/A	37.6	N/A	99.6		N/A	N/A	
	13/06/15	Yes	10.97	37.2	No	99		0	0	
	14/06/15	Yes	10.82	37.3	N/A	99.2		N/A	N/A	
159	13/12/14	Yes (not new or progressive)	4.87	37.6	N/A	99.6		N/A	N/A	
	14/12/14	Yes (not new or progressive)	4.72	37.6	Yes	99.6		0	2+ ENTA	
	15/12/14	Yes (not new or progressive)	5.11	38.5	N/A	101.3		N/A	N/A	
160	15/12/14	No	33.76	38.2	No	100.7		0	0	
	16/12/14	No	23.11	37.8	N/A	100		N/A	N/A	
	17/12/14	No	24.38	37.1	N/A	98.7		N/A	N/A	
161	16/12/14	Yes (not new or progressive)	12.71	38.1	N/A	100.6		N/A	N/A	
	17/12/14	Yes (not new or progressive)	9.52	37.6	N/A	99.6		N/A	N/A	
	18/12/14	Yes (not new or progressive)	7.76	37.2	Yes	98.9		1+	0	
	19/12/14	Yes (not new or progressive)	7.18	37.5	No	99.5		1+	0	
	20/12/14	Yes (not new or progressive)	7.17	37.8	Yes	100		3+	0	
	21/12/14	No	12.44	32.9	N/A	91.3		N/A	N/A	
162	03/06/15	Yes (not new or progressive)	10.15	30.5	Yes	86.9		0	0	
	04/06/15	No	6.53	31.8	Yes	89.2		0	0	
	05/06/15	Yes (new)	9.09	38.6	No	101.5		0	0	
	06/06/15	No	10.08	37.8	Yes	100		2+	0	
	07/06/15	N/A	N/A	37.2	Yes	99		2+	0	
163	19/12/14	No	3.88	37.5	No	99.5		0	0	
	20/12/14	Yes (new)	4.57	37.1	Yes	98.8		0	0	
	21/12/14	Yes	4.16	38.8	Yes	101.8	VAP	N/A	N/A	
	22/12/14	Yes	7.32	40.9	No	105.7		N/A	N/A	
	23/12/14	Yes	6.16	39.9	Yes		VAP	0	0	
	24/12/14	N/A	N/A	38.8	N/A			N/A	N/A	
164	21/12/14	Yes (not new or progressive)	28.19	37.6	No	99.6		0	0	
	22/12/14	Yes (not new or progressive)	21.64	38.2	Yes	100.7		0	0	
	23/12/14	Yes (not new or progressive)	19.15	38.0	No	100.4		0	0	
	24/12/14	Yes (not new or progressive)	14.08	38.1	N/A	100.6		N/A	N/A	
	25/12/14	Yes (not new or progressive)	14.86	37.8	N/A	99.98		N/A	N/A	
	26/12/14	Yes (not new or progressive)	15.93	37.8	Yes	100.1		0	0	
	27/12/14	Yes (not new or progressive)	17.86	38.1	Yes	100.6		0	0	
	28/12/14	Yes (not new or progressive)	13.97	38.4	Yes	101.1		0	0	
	29/12/14	Yes (not new or progressive)	14.11	38.6	Yes	101.5		0	1+ EC	
	30/12/14	Yes (not new or progressive)	14.51	37.5	Yes	99.5		0	0	
	31/12/14	Yes (not new or progressive)	12.4	38.3	N/A	101		N/A	N/A	
	01/01/15	Yes (not new or progressive)	11.13	38.1	N/A	100.5		N/A	N/A	
	02/01/15	Yes (not new or progressive)	10.08	38.0	No	100.4		0	0	
	03/01/15	Yes (not new or progressive)	8.69	37.9	No	100.2		0	0	
	04/01/15	N/A	8.74	37.5	No	99.5		0	0	
	05/01/15	Yes (not new or progressive)	9.51	37.2	N/A	98.9		N/A	N/A	
	06/01/15	Yes (not new or progressive)	10.26	37.6	N/A	99.7		N/A	N/A	
	07/01/15	Yes (not new or progressive)	9.05	38.1	N/A	100.6		0	0	
	08/01/15	Yes (not new or progressive)	8.97	38.2	N/A	100.7		N/A	N/A	
165	21/12/14	Yes (not new or progressive)	20.42	36.8	Yes	98.2		2+	0	
	22/12/14	Yes (not new or progressive)	11.93	37.1	Yes	98.8		0	0	
	23/12/14	Yes (not new or progressive)	11.52	37.5	N/A	99.5		N/A	N/A	
166	27/12/14	Yes (not new or progressive)	11.91	36.9	No	98.4		N/A	N/A	
	28/12/14	Yes (not new or progressive)	7.24	36.5	Yes	97.7		0	0	
	29/12/14	No	3.31	36.7	No	98		0	0	
	30/12/14	No	3.37	36.8	No	98.3		0	0	
	31/12/14	Yes (new)	2.74	36.6	N/A	97.8		N/A	N/A	
	01/01/15	No	3.76	37.1	N/A	98.7		N/A	N/A	
167	28/12/14	Yes (not new or progressive)	31.57	36.7	Yes	98		0	0	
	29/12/14	Yes (not new or progressive)	34.8	36.7	N/A	98		N/A	N/A	
	30/12/14	Yes (not new or progressive)	36.59	36.3	Yes	97.4		0	0	
	31/12/14	Yes (not new or progressive)	22.08	36.9	N/A			N/A	N/A	
	01/01/15	Yes (not new or progressive)	11.47	36.7	N/A			N/A	N/A	
	02/01/15	Yes (not new or progressive)	16.55	36.7	Yes	98.1		0	3+ GNR (MOST BKCC)	
	03/01/15	No	16.18	36.7	No			0	2+ GNR (MOST BKCC)	
	04/01/15	No	18.42	37.0	Yes	98.6		0	0	

	05/01/15	No	15.76	36.6	Yes	97.9		0	2+ GNR (MOST BKCC)
	06/01/15	No	12.71	37.5	Yes	99.5		0	4+ GNR (MOST BKCC)
	07/01/15	No	19.89	37.3	N/A	99.1		N/A	N/A
168	28/12/14	Yes (not new or progressive)	30.67	38.9	N/A	102.1		N/A	N/A
	29/12/14	Yes (not new or progressive)	24.88	37.6	Yes	99.6		N/A	N/A
	30/12/14	Yes (not new or progressive)	26.92	37.1	N/A	98.8		N/A	N/A
	31/12/14	N/A	N/A	N/A	N/A			N/A	N/A
	01/01/15	N/A	N/A	N/A	N/A			N/A	N/A
169	29/12/14	Yes (not new or progressive)	13.57	37.8	Yes	100.1		N/A	N/A
	30/12/14	No	11.22	37.6	Yes	99.6		2+	0
	31/12/14	Yes (new)	7.92	36.9	N/A	98.5		N/A	N/A
	01/01/15	Yes	8.56	37.2	Yes	98.9		N/A	N/A
	02/01/15	Yes	9.23	37.2	Yes	98.9		3+	0
	03/01/15	Yes	9.35	37.6	No	99.6		4+	3+ BKCC
	04/01/15	Yes	9.61	37.2	N/A	99		N/A	N/A
	05/01/15	Yes	8.61	37.1	N/A	98.7		N/A	N/A
	06/01/15	Yes	8.13	36.9	N/A	98.5		N/A	N/A
170	29/12/14	No	9.21	40.1	No	104.1		0	0
	30/12/14	Yes (new)	14.14	39.0	No			0	0
	31/12/14	Yes	10.01	38.5	N/A	101.3		N/A	N/A
	01/01/15	Yes	12.72	37.3	N/A	99.1		N/A	N/A
	02/01/15	No	8.59	39.8	No			0	0
	03/01/15	Yes (new)	9.76	39.3	No	102.7		0	0
	04/01/15	Yes	17.95	38.1	No		VAP	0	0
	05/01/15	Yes	9.1	36.9	No			0	0
	06/01/15	Yes	7.69	36.6	N/A			N/A	N/A
	07/01/15	No	6.15	36.8	Yes	98.3		0	0
	08/01/15	No	6.74	37.7	No	99.8		0	0
	09/01/15	Yes (new)	7.8	37.2	No	98.9		0	0
	10/01/15	Yes	6.63	36.6	N/A	97.9		N/A	N/A
	11/01/15	Yes	5.81	37.9	Yes	100.3		0	0
	12/01/15	No	6.14	37.4	N/A	99.4		N/A	N/A
171	01/01/15	Yes (not new or progressive)	31.79	36.8	Yes	98.3		N/A	N/A
	02/01/15	Yes (not new or progressive)	55.89	37.2	N/A	98.9		0	0
	03/01/15	Yes (not new or progressive)	65.83	36.8	N/A	98.2		0	0
	04/01/15	N/A	N/A	N/A	N/A			N/A	N/A
172	30/12/14	Yes (not new or progressive)	N/A	37.2	Yes	98.9		0	0
	31/12/14	Yes (not new or progressive)	32.8	36.7	N/A	98		N/A	N/A
	01/01/15	Yes (not new or progressive)	27.91	36.6	N/A	97.9		N/A	N/A
	02/01/15	Yes (not new or progressive)	31.45	36.9	Yes	98.5		0	0
	03/01/15	Yes (not new or progressive)	24.12	36.9	No	98.5		0	0
	04/01/15	Yes (not new or progressive)	21.56	37.3	Yes	99.1		0	0
	05/01/15	Yes (not new or progressive)	19.62	37.6	Yes	99.7		0	0
	06/01/15	Yes (not new or progressive)	23.31	38.0	No	100.4		0	0
	07/01/15	Yes (not new or progressive)	22.91	37.5	No	99.5		N/A	N/A
	08/01/15	Yes (not new or progressive)	24.12	37.3	Yes	99.2		0	0
	09/01/15	Yes (progressive)	22.14	38.1	No	100.5	VAP	0	0
	10/01/15	Yes	21.36	37.2	Yes	98.9	VAP	0	0
	11/01/15	Yes	24.41	37.3	Yes	99.1	VAP	0	0
	12/01/15	Yes	20.16	36.9	No	98.4		0	0
	13/01/15	No	17.96	37.2	Yes	99		0	0
	14/01/15	No	15.64	37.4	N/A	99.3		N/A	N/A
	15/01/15	Yes (new)	17.03	36.9	Yes	98.5	VAP	0	0
	16/01/15	N/A	12.51	37.0	No	98.6		0	0
	17/01/15	No	8.8	37.4	No	99.3		0	0
	18/01/15	No	N/A	38.1	Yes	100.6		0	0
	19/01/15	Yes (new)	7.84	38.1	No	100.5		0	0
	20/01/15	Yes	8.63	37.1	N/A	98.8		0	0
	21/01/15	N/A	N/A	N/A	N/A			N/A	N/A
	22/01/15	N/A	N/A	N/A	N/A			N/A	N/A
173	30/12/14	Yes (not new or progressive)	18.22	36.6	N/A	97.8		N/A	N/A
	31/12/14	Yes (not new or progressive)	11.94	37.1	N/A	98.8		N/A	N/A
	01/01/15	Yes (not new or progressive)	12.02	36.9	N/A	98.4		N/A	N/A
	02/01/15	No	19.35	36.5	Yes	97.7		0	4+ SERM
	03/01/15	No	12.95	36.6	N/A	97.9		N/A	N/A
	04/01/15	No	15.23	36.6	N/A	97.9		N/A	N/A
174	30/12/14	No	11.09	36.8	No	98.2		0	0
	31/12/14	No	9.72	37.1	N/A	98.7		N/A	N/A
	01/01/15	Yes (new)	10.2	36.9	N/A	98.4		N/A	N/A
175	30/12/14	Yes (not new or progressive)	6.71	39.7	Yes	103.4		0	0
	31/12/14	Yes (not new or progressive)	5.1	37.6	N/A	99.6		N/A	N/A
	01/01/15	Yes (not new or progressive)	5.11	37.1	N/A	98.8		N/A	N/A
	02/01/15	Yes (not new or progressive)	3.99	36.8	No	98.2		0	0
	03/01/15	Yes (not new or progressive)	5.31	36.9	N/A	98.4		N/A	N/A
	04/01/15	Yes (not new or progressive)	6.06	36.7	N/A	98.1		N/A	N/A
176	30/12/14	No	9.71	36.4	Yes	97.6		0	0
	31/12/14	No	8.2	36.9	N/A	98.4		N/A	N/A
	01/01/15	No	9.8	36.9	N/A	98.5		N/A	N/A
	02/01/15	No	13.9	37.6	Yes	99.6		0	0
	03/01/15	No	19.37	37.3	N/A	99.1		N/A	N/A
	04/01/15	No	18.22	37.2	N/A	98.9		N/A	N/A
177	03/01/15	Yes (not new or progressive)	12.06	36.7	Yes	98.1		0	4+ PSA
	04/01/15	N/A	24.5	36.8	No	98.2		0	3+ PSA
	05/01/15	N/A	N/A	36.9	Yes	98.5		0	3+ PSA
	06/01/15	N/A	N/A	N/A	N/A			N/A	N/A
178	03/01/15	Yes (not new or progressive)	14.47	39.4	Yes	103		0	2+ MCAT
	04/01/15	Yes (not new or progressive)	11.9	39.3	Yes	102.7		0	0
	05/01/15	Yes (not new or progressive)	6.53	37.6	Yes	99.7		0	0
	06/01/15	No	4.69	37.4	N/A	99.4		N/A	N/A
179	04/06/15	Yes (not new or progressive)	17.34	37.2	Yes	99		2+	0
	05/06/15	Yes (not new or progressive)	16.2	37.3	N/A	99.2		N/A	N/A
	06/06/15	Yes (not new or progressive)	11.46	37.4	Yes	99.3		0	0
	07/06/15	Yes (not new or progressive)	9.45	37.7	Yes	99.8		2+	0
	08/06/15	Yes (not new or progressive)	8.94	38.0	No	100.4		1+	0
	09/06/15	Yes (progressive)	7.92	37.9	Yes	100.2		1+	0
	10/06/15	Yes	8.17	37.1	Yes	98.7		1+	0
	11/06/15	Yes	N/A	36.8	Yes	98.2		1+	0
	12/06/15	Yes	8.7	36.8	Yes	98.3		2+	0
	13/06/15	Yes	8.2	36.8	No	98.2		1+	0
	14/06/15	Yes	8.49	37.2	N/A	99		N/A	N/A
	15/06/15	Yes	N/A	36.9	Yes	98.4		1+	0
	16/06/15	Yes	6.76	37.1	Yes	98.8		0	0
180	04/01/15	No	3.9	33.7	Yes	92.6		0	4+ KP; 2+ PMIR
	05/01/15	No	6.36	38.7	N/A	101.6		0	3+ KP; 2+ PMIR
	06/01/15	Yes (new)	N/A	37.9	N/A	100.2		N/A	N/A
	07/01/15	Yes	6.41	N/A	N/A			N/A	N/A
181	06/06/15	Yes (not new or progressive)	17.36	37.7	No	99.9		0	0
	07/06/15	Yes (not new or progressive)	18.17	37.6	No	99.6		0	0
	08/06/15	Yes (not new or progressive)	15.89	37.7	No	99.9		0	0
	09/06/15	N/A	14.73	38.2	No	100.8		N/A	N/A
	10/06/15	Yes (progressive)	13.25	38.9	No	102.1	VAP	0	0

	11/06/15	Yes	13.05	37.3	No	99.2		0	0
	12/06/15	N/A	12.74	38.7	Yes	101.6		0	0
	13/06/15	Yes	12.67	38.6	No	101.4	VAP	N/A	N/A
	14/06/15	Yes	12.32	37.6	N/A	99.6		N/A	N/A
	15/06/15	Yes (progressive)	13.13	37.2	No	98.9		0	0
	16/06/15	Yes	14.75	37.4	No	99.4		0	0
	17/06/15	Yes (progressive)	18.86	38.2	No	100.8	VAP	0	0
	18/06/15	Yes	18.75	39.4	No	102.9	VAP	0	0
	19/06/15	Yes	16.68	38.9	No	102.1	VAP	0	0
	20/06/15	Yes	15.63	37.8	No	100		0	0
	21/06/15	Yes	15.51	38.5	No	101.3	VAP	0	0
	22/06/15	Yes	16.64	38.7	No	101.6	VAP	0	0
	23/06/15	Yes	17.98	38.7	No	101.7	VAP	0	0
	24/06/15	Yes	22.99	37.8	No	100		0	0
	25/06/15	Yes	17.87	37.6	No	99.7		0	0
	26/06/15	Yes	15.72	38.4	No	101.1	VAP	0	0
	27/06/15	N/A	N/A	36.9	N/A	98.4		N/A	N/A
182	07/01/15	N/A	8.14	36.9	Yes	98.4		4+	0
	08/01/15	No	4.73	36.9	Yes	98.4		4+	0
	09/01/15	No	6.16	36.9	No	98.4		4+	0
	10/01/15	Yes (new)	7.27	36.6	N/A	97.9		N/A	N/A
183	07/01/15	N/A	16.92	37.3	Yes	99.1		0	0
	08/01/15	Yes (not new or progressive)	16.64	37.3	No	99.1		0	0
	09/01/15	Yes (not new or progressive)	14.6	37.4	No	99.3		0	0
	10/01/15	Yes (not new or progressive)	11.41	37.3	No	99.2		N/A	N/A
	11/01/15	Yes (not new or progressive)	11.37	37.7	No	99.9		N/A	N/A
	12/01/15	Yes (not new or progressive)	11.17	37.9	No	100.2		0	0
	13/01/15	Yes (not new or progressive)	11.92	37.8	No	100.1		0	0
	14/01/15	Yes (not new or progressive)	15.08	37.3	Yes	99.2		0	0
	15/01/15	Yes (not new or progressive)	14.28	37.1	No	98.8		0	0
	16/01/15	Yes (not new or progressive)	12.75	37.3	No	99.2		0	0
	17/01/15	Yes (not new or progressive)	13.69	36.9	No	98.4		0	0
	18/01/15	Yes (not new or progressive)	16.19	37.2	No	99		0	0
	19/01/15	Yes (not new or progressive)	20.76	37.4	Yes	99.3		0	0
	20/01/15	Yes (progressive)	21.44	37.0	No	98.6		N/A	N/A
	21/01/15	Yes	N/A	37.5	No	99.5		0	1+ CTFX
	22/01/15	Yes	16.39	37.9	No	100.3		0	0
	23/01/15	Yes	15.88	38.1	Yes	100.5	VAP	0	2+ CTFX
	24/01/15	Yes	13.87	37.3	N/A	99.2		N/A	N/A
	25/01/15	Yes	15.15	37.3	No	99.2		0	2+ CTFX
	26/01/15	Yes	16.84	37.7	Yes	99.9	VAP	0	3+ CTFX
	27/01/15	Yes	14.61	38.5	Yes	101.3	VAP	0	3+ CTFX; 3+ PSA
	28/01/15	Yes	14.84	38.4	N/A	101.2	VAP	N/A	N/A
184	11/01/15	Yes (not new or progressive)	9.82	38.4	Yes	101.2		0	0
	12/01/15	Yes (not new or progressive)	10.21	37.2	No	99		2+	0
	13/01/15	Yes (not new or progressive)	25.2	37.7	No	99.9		1+	0
	14/01/15	N/A	N/A	37.3	N/A	99.1		N/A	N/A
	15/01/15	N/A	N/A	N/A	N/A			N/A	N/A
185	11/01/15	No	13.85	37.8	Yes	100		0	0
	12/01/15	Yes (new)	9.18	37.1	No	98.8		0	0
	13/01/15	No	9.44	37.1	N/A	98.7		N/A	N/A
186	12/01/15	Yes (not new or progressive)	14.5	39.9	No	103.8		0	0
	13/01/15	Yes (not new or progressive)	7.36	39.5	Yes	103.1		0	0
	14/01/15	Yes (not new or progressive)	5.62	36.2	No	97.1		0	0
	15/01/15	Yes (not new or progressive)	11.76	36.6	No	97.8		0	0
	16/01/15	No	16.84	36.6	N/A	97.8		N/A	N/A
	17/01/15	No	11.7	36.6	Yes	97.8		0	0
	18/01/15	Yes (new)	14.39	36.8	Yes	98.3	VAP	0	0
	19/01/15	Yes	19.09	37.0	Yes	98.6	VAP	0	0
	20/01/15	Yes	24.63	36.6	Yes	97.8	VAP	0	0
	21/01/15	Yes	54.81	37.1	No	98.8		0	0
	22/01/15	Yes	40.55	37.4	No	99.3		0	0
	23/01/15	Yes	39.64	38.1	Yes	100.5	VAP	0	0
	24/01/15	Yes	42.42	37.9	N/A	100.2		N/A	N/A
	25/01/15	Yes	28.92	36.0	Yes	96.8	VAP	0	0
	26/01/15	Yes	34.42	36.5	Yes	97.7	VAP	0	0
	27/01/15	Yes	33.17	37.2	N/A	98.9		N/A	N/A
	28/01/15	No	29.3	36.9	N/A	98.5		N/A	N/A
	29/01/15	No	28.36	36.9	Yes	98.4		0	0
	30/01/15	Yes (new)	28.64	37.2	Yes	99	VAP	0	0
	31/01/15	No	25.15	37.0	Yes	98.6		0	0
	01/02/15	N/A	17.93	37.2	Yes	98.9		0	0
	02/02/15	Yes (new)	15.63	37.2	Yes	99	VAP	0	0
	03/02/15	No	14.32	37.7	Yes	99.9		0	0
	04/02/15	No	19.38	38.1	Yes	100.5		0	0
	05/02/15	No	18.38	38.3	N/A	100.9		N/A	N/A
187	08/06/15	Yes (not new or progressive)	9.73	38.8	No	101.9		1+	0
	09/06/15	Yes (not new or progressive)	8.2	39.4	No	103		N/A	N/A
	10/06/15	Yes (not new or progressive)	6.52	39.1	No	102.3		0	0
	11/06/15	No	N/A	38.0	Yes	100.4		0	0
	12/06/15	No	7.46	37.8	Yes	100.1		0	0
	13/06/15	Yes (new)	9.31	37.9	Yes	100.3		0	0
	14/06/15	No	12.37	37.9	N/A	100.3		N/A	N/A
	15/06/15	Yes (new)	17.58	37.5	Yes	99.5	VAP	0	0
	16/06/15	No	17.2	37.6	Yes	99.7		0	0
	17/06/15	No	15.73	37.8	No	100		0	0
	18/06/15	No	18.93	37.3	No	99.1		0	0
	19/06/15	N/A	N/A	37.1	N/A	98.7		N/A	N/A
188	16/01/15	No	11.8	38.2	N/A	100.7		N/A	N/A
	17/01/15	No	12.79	38.0	No	100.4		0	0
	18/01/15	No	13.13	38.1	No	100.6		0	0
	19/01/15	Yes (new)	12.76	37.3	N/A	99.2		N/A	N/A
	20/01/15	Yes	14.01	37.8	N/A	100.1		N/A	N/A
189	17/01/15	Yes (not new or progressive)	10.16	37.9	No	100.2		0	0
	18/01/15	Yes (not new or progressive)	9	37.5	No	99.5		0	0
	19/01/15	Yes (not new or progressive)	11.01	37.6	Yes	99.6		0	0
	20/01/15	No	10.2	37.3	N/A	99.1		N/A	N/A
	21/01/15	No	12.67	37.0	N/A	98.6		N/A	N/A
190	17/01/15	Yes (not new or progressive)	17.97	37.4	N/A	99.3		N/A	N/A
	18/01/15	No	14.36	37.0	No	98.6		0	0
	19/01/15	Yes (new)	14.23	37.1	N/A	98.7		N/A	N/A
	20/01/15	Yes	13.3	36.8	Yes	98.3	VAP	0	0
	21/01/15	Yes	13.3	37.2	No	98.9		0	0
	22/01/15	Yes	13.3	36.9	No	98.5		0	0
	23/01/15	Yes	13.01	37.3	Yes	99.1	VAP	0	0
	24/01/15	Yes	14.11	37.9	No	100.2		0	0
	25/01/15	Yes	16.55	38.2	Yes	100.7	VAP	0	0
	26/01/15	Yes	17.66	37.6	N/A	99.7		N/A	N/A
	27/01/15	Yes	14.94	37.7	N/A	99.9		N/A	N/A
	28/01/15	No	12.47	36.8	No	98.3		0	0
	29/01/15	N/A	N/A	N/A	N/A			N/A	N/A
191	18/01/15	Yes (not new or progressive)	11.56	36.8	No	98.2		0	0
	19/01/15	No	11.17	37.1	Yes	98.7		0	0

	20/01/15	No	13.53	38.2	No	100.7		N/A	N/A
	21/01/15	No	13.13	37.6	N/A	99.6		0	0
	22/01/15	Yes (new)	15.69	36.7	No	98.1		0	0
	23/01/15	Yes	19.07	37.3	Yes	99.1	VAP	0	0
	24/01/15	Yes	14.39	37.2	N/A	98.9		N/A	N/A
	25/01/15	Yes	16.09	37.1	Yes	98.7	VAP	0	1+ PSA; 2+ PSA (OMOR)
	26/01/15	Yes	21.2	37.3	Yes	99.1	VAP	0	3+ PSA
	27/01/15	Yes	16.17	37.2	Yes	98.9	VAP	0	4+ PSA
	28/01/15	Yes	17.84	37.2	Yes	98.9	VAP	0	3+ PSA
	29/01/15	Yes	15.37	38.1	Yes	100.5	VAP	0	2+ PSA
	30/01/15	Yes	13.39	37.2	Yes	99	VAP	0	3+ PSA
	31/01/15	Yes	15.07	37.6	Yes	99.7	VAP	0	4+ PSA (MORP2)
	01/02/15	Yes	13.07	36.7	N/A	98		N/A	N/A
192	19/01/15	No	15.4	38.2	N/A	100.7		N/A	N/A
	20/01/15	No	21.96	37.1	Yes	98.8		0	0
	21/01/15	No	19.5	38.0	No	100.4		0	0
	22/01/15	No	17.72	37.5	Yes	99.5		0	0
193	09/06/15	Yes (not new or progressive)	20.89	37.6	No	99.7		0	0
	10/06/15	Yes (not new or progressive)	46.29	38.0	No	100.4		0	0
194	22/01/15	No	20.16	39.3	N/A	102.7		N/A	N/A
	23/01/15	No	26.39	39.7	Yes	103.4		0	0
	24/01/15	N/A	N/A	N/A	N/A			N/A	N/A
195	29/01/15	Yes (not new or progressive)	12.53	37.4	N/A	99.3		N/A	N/A
	30/01/15	No	12.19	38.6	Yes	101.4		0	0
	31/01/15	No	11.12	37.3	No	99.1		0	0
	01/02/15	No	7.99	38.2	No	100.7		0	0
	02/02/15	Yes (new)	9.92	37.7	Yes	99.9		0	0
	03/02/15	No	11.53	37.8	No	100.1		0	0
	04/02/15	Yes (new)	13.08	38.2	Yes	100.7	VAP	0	0
	05/02/15	Yes	14.15	37.3	N/A	99.1		N/A	N/A
	06/02/15	No	14.92	37.0	N/A	98.6		N/A	N/A
	07/02/15	Yes (new)	15.35	37.2	N/A	98.9		N/A	N/A
196	31/01/15	No	4.75	38.5	No			0	0
	01/02/15	No	25.59	37.4	No			0	0
	02/02/15	No	25.89	37.1	N/A	98.7		N/A	N/A
	03/02/15	No	18.38	37.3	No	99.1		0	0
	04/02/15	Yes (new)	21.43	37.2	No	98.9		0	0
	05/02/15	Yes	27.06	37.7	N/A	99.9		N/A	N/A
	06/02/15	Yes	39.01	37.2	No	98.9		N/A	N/A
	07/02/15	Yes	39.18	37.4	Yes	99.3	VAP	0	0
	08/02/15	Yes	27.7	37.3	No	99.1		0	0
	09/02/15	Yes	18.67	37.5	No	99.5		0	0
	10/02/15	Yes	15.48	37.8	N/A	100.1		N/A	N/A
	11/02/15	Yes	15.11	37.3	No	99.1		0	0
	12/02/15	Yes	13.33	36.9	N/A	98.5		N/A	N/A
	13/02/15	Yes	10.31	37.1	N/A	98.8		N/A	N/A
	14/02/15	Yes	9.68	37.1	No	98.8		0	0
197	01/02/15	Yes (not new or progressive)	14.75	36.8	Yes	98.2		0	4+ SERM
	02/02/15	Yes (not new or progressive)	15.87	36.0	Yes	96.8		0	4+ SERM
	03/02/15	No	18.11	37.0	Yes	98.6		0	4+ SERM; 4+ ACCL
	04/02/15	Yes (new)	15.4	37.3	N/A	99.1		N/A	N/A
198	08/02/15	Yes (not new or progressive)	15.27	37.1	N/A	98.7		N/A	N/A
	09/02/15	Yes (not new or progressive)	10.58	38.8	Yes	101.8		0	0
	10/02/15	Yes (not new or progressive)	9.28	37.9	Yes	100.2		0	0
	11/02/15	Yes (not new or progressive)	7.99	37.7	Yes	99.8		0	0
	12/02/15	Yes (not new or progressive)	7.05	37.3	Yes	99.1		0	0
	13/02/15	No	8.99	37.3	No	99.2		0	0
	14/02/15	No	7.94	37.1	N/A	98.7		N/A	N/A
	15/02/15	N/A	7.96	37.3	N/A	99.1		N/A	N/A
	16/02/15	N/A	N/A	37.2	N/A	98.9		N/A	N/A
199	08/02/15	Yes (not new or progressive)	8.82	40.6	N/A	105		N/A	N/A
	09/02/15	No	10.96	37.8	Yes	100		0	0
	10/02/15	No	9.8	37.2	No	99		0	0
	11/02/15	No	8.61	37.2	Yes	99		0	0
	12/02/15	Yes (new)	9.17	36.9	Yes	98.5		0	0
	13/02/15	Yes	8.08	36.8	No	98.3		0	0
	14/02/15	N/A	N/A	37.1	Yes	98.7		0	0
	15/02/15	Yes (progressive)	7.65	37.2	Yes	99		0	0
	16/02/15	Yes	10.91	37.2	Yes	99		0	0
	17/02/15	No	11.65	37.1	Yes	98.8		0	0
	18/02/15	No	9.56	37.3	No	99.2		0	0
	19/02/15	No	11.52	37.3	Yes	99.2		0	0
	20/02/15	No	12.41	37.4	No	99.4		0	0
	21/02/15	No	10.71	37.1	No	98.8		0	0
	22/02/15	No	8.55	36.9	N/A	98.4		N/A	N/A
	23/02/15	Yes (new)	8.42	36.7	No	98		0	0
	24/02/15	Yes	7.68	36.8	No	98.3		0	0
	25/02/15	No	8.83	37.1	No	98.7		0	0
	26/02/15	No	8.84	36.7	No	98		N/A	N/A
200	08/02/15	No	27.96	37.5	N/A	99.5		N/A	N/A
	09/02/15	Yes (new)	23.69	38.5	Yes	101.3	VAP	0	0
	10/02/15	No	24.44	39.7	Yes	103.4		0	0
	11/02/15	No	36.99	37.8	No	100		0	0
	12/02/15	No	40.28	38.3	N/A	100.9		N/A	N/A
	13/02/15	No	40.41	37.0	N/A	98.6		N/A	N/A
	14/02/15	No	36.56	38.3	N/A	100.9		N/A	N/A
	15/02/15	No	31.65	38.5	N/A	101.3		N/A	N/A
	16/02/15	No	17.37	N/A	N/A			N/A	N/A
201	11/02/15	No	5.24	36.7	Yes	98		0	0
	12/02/15	Yes (new)	5.17	39.0	Yes	102.2		0	0
	13/02/15	No	5.9	38.3	No	100.9		0	0
	14/02/15	Yes (new)	9.2	37.2	Yes	98.9		0	0
	15/02/15	No	8.72	36.9	Yes	98.4		0	0
	16/02/15	No	9.53	37.2	No	98.9		0	0
	17/02/15	No	8.98	37.1	No	98.7		0	0
	18/02/15	Yes (new)	8.12	36.8	No	98.2		1+	0
	19/02/15	Yes	8.55	37.1	No	98.8		2+	0
	20/02/15	Yes	10.02	37.4	No	99.3		2+	0
202	11/02/15	Yes (not new or progressive)	25.44	37.8	Yes	100.1		0	0
	12/02/15	Yes (not new or progressive)	30.6	38.4	N/A	101.2		N/A	N/A
	13/02/15	N/A	N/A	N/A	Yes			N/A	N/A
203	12/06/15	No	16.17	37.1	Yes	98.7		0	4+ DIPS; 2+ BTSF
	13/06/15	Yes (new)	19	39.4	No	102.9		0	1+ EC
204	13/02/15	Yes (not new or progressive)	17.33	36.9	Yes	98.5		0	0
	14/02/15	No	15.34	37.4	Yes	99.3		0	0
	15/02/15	No	11.3	36.9	N/A	98.4		0	0
	16/02/15	No	8.55	36.9	N/A	98.5		N/A	N/A
	17/02/15	No	10.44	37.2	Yes	99		N/A	N/A
205	14/02/15	No	16.73	36.4	N/A	97.6		N/A	N/A
	15/02/15	No	16.96	37.6	Yes	99.6		0	0
	16/02/15	Yes (new)	24.67	38.6	N/A	101.5	VAP	0	0
	17/02/15	Yes	27.54	38.2	N/A	100.7	VAP	N/A	N/A

	18/02/15	Yes	25.52	37.8	N/A	100.1		N/A	N/A	
206	16/02/15	No	17.72	37.9	No	100.2		N/A	N/A	
	17/02/15	Yes (new)	17.46	37.9	No	100.2		0	0	
	18/02/15	Yes	11.17	38.2	No	100.7	VAP	0	0	
	19/02/15	Yes	11.41	37.4	No	99.3		0	0	
	20/02/15	Yes	17.49	36.8	N/A	98.2		0	0	
	21/02/15	Yes	16.6	36.7	N/A	98.1		N/A	N/A	
	22/02/15	No	16.29	36.9	N/A	98.4		N/A	N/A	
	23/02/15	Yes (new)	16.48	37.1	Yes	98.7	VAP	N/A	N/A	
207	17/02/15	Yes (not new or progressive)	11.02	37.1	Yes	98.8		0	0	
	18/02/15	Yes (not new or progressive)	11.85	37.7	No	99.8		0	0	
	19/02/15	Yes (not new or progressive)	12.93	37.7	No	99.9		0	0	
	20/02/15	Yes (progressive)	14.43	37.4	No	99.4		0	0	
208	18/02/15	Yes (not new or progressive)	15.63	37.7	Yes	99.8		2+	0	
	19/02/15	No	15.5	37.8	No	100		0	3+ ACCA; 3+ KP	
	20/02/15	Yes (new)	11.03	38.1	Yes	100.5	VAP	0	3+ ACCA; 3+ KP	
	21/02/15	No	10.29	37.9	N/A	100.2		N/A	N/A	
	22/02/15	Yes (new)	10.67	37.7	Yes	99.9		N/A	N/A	
	23/02/15	Yes (progressive)	10.75	37.1	N/A	98.8		0	3+ ACCA; 3+ KP	
	24/02/15	Yes	12.42	37.6	N/A	99.6		N/A	N/A	
	25/02/15	Yes	13.34	37.2	N/A	99		N/A	N/A	
	26/02/15	Yes	11.34	N/A	Yes		VAP	N/A	N/A	
209	21/02/15	No	37.76	37.6	N/A	99.6		N/A	N/A	
	22/02/15	Yes (new)	16.91	38.3	No	100.9	VAP	N/A	N/A	
	23/02/15	Yes (progressive)	13.29	37.7	N/A	99.9		0	0	
	24/02/15	Yes	11.46	37.2	No	98.9		N/A	N/A	
	25/02/15	Yes	14.04	37.4	Yes	99.3	VAP	0	0	
	26/02/15	No	20.49	37.6	No	99.6		0	0	
	27/02/15	Yes (new)	17.56	37.7	Yes	99.8	VAP	0	0	
	28/02/15	Yes	18.04	37.4	No	99.4		0	0	
	01/03/15	No	13.48	37.4	Yes	99.3		0	0	
	02/03/15	Yes (new)	12.7	37.7	N/A	99.9		0	0	
	03/03/15	N/A	9.71	37.1	No	98.8		N/A	N/A	
210	27/02/15	No	9.59	38.3	No	101		0	2+ KOX; 2+ EC	
	28/02/15	No	10.83	39.0	No	102.2		2+	2+ EC	
	01/03/15	Yes (new)	21.62	38.1	No	100.6	VAP	2+	0	
	02/03/15	Yes	18.41	38.1	No	100.6	VAP	3+	2+ EC; 3+ KOX	MRSA
	03/03/15	Yes	13.48	37.9	N/A	100.3		2+	1+ EC	
	04/03/15	Yes	13.64	37.7	N/A	99.8		N/A	N/A	
	05/03/15	Yes	11.46	37.7	No	99.9		N/A	N/A	
211	27/02/15	No	14.01	37.9	No	100.3		0	1+ KP; 1+ EC	
	28/02/15	Yes (new)	9.12	37.7	No	99.9		0	0	
	01/03/15	No	5.59	37.7	Yes	99.8		0	3+ EC	
	02/03/15	Yes (new)	7.25	38.2	Yes	100.8	VAP	0	3+ EC	
	03/03/15	Yes	8.94	37.2	N/A	98.9		0	2+ KP; 2+ EC	
	04/03/15	Yes	10.21	38.5	N/A	101.3		N/A	N/A	
	05/03/15	Yes	11.86	37.2	No	98.9		N/A	N/A	
212	27/02/15	No	5.21	38.4	Yes	101.1		0	0	
	28/02/15	No	5.92	38.8	No	101.8		0	0	
	01/03/15	No	6.18	39.6	Yes	103.3		0	0	
	02/03/15	Yes (new)	6.62	39.3	No	102.7		0	0	
	03/03/15	Yes	8.05	38.2	N/A	100.8		0	0	
	04/03/15	No	9.83	38.2	N/A	100.8		N/A	N/A	
	05/03/15	No	N/A	37.7	N/A	99.9		N/A	N/A	
	06/03/15	No	11.76	37.7	No	99.9		N/A	N/A	
	07/03/15	Yes (new)	15.9	37.6	No	99.6		0	0	
	08/03/15	Yes	13.52	37.6	N/A	99.7		0	0	
	09/03/15	Yes	13.96	38.3	N/A	101	VAP	N/A	N/A	
	10/03/15	No	12.98	38.3	No	100.9		N/A	N/A	
	11/03/15	No	11.57	39.5	Yes	103.1		0	0	
	12/03/15	No	12.71	38.7	No	101.7		0	0	
213	03/03/15	No	4.84	39.7	Yes	103.4		0	0	
	04/03/15	Yes (new)	7.48	37.2	No	98.9		0	0	
	05/03/15	Yes	7.35	37.0	N/A	98.6		0	0	
	06/03/15	Yes	7.29	36.9	No	98.5		0	0	
	07/03/15	No	7.46	38.2	N/A	100.8		0	0	
	08/03/15	No	8.04	38.2	N/A	100.7		N/A	N/A	
	09/03/15	No	8.76	38.3	Yes	101		N/A	N/A	
	10/03/15	Yes (new)	9.62	38.9	N/A	102		0	0	
	11/03/15	N/A	N/A	37.7	N/A	99.8		N/A	N/A	
	12/03/15	N/A	N/A	N/A	No			N/A	N/A	
214	05/03/15	Yes (not new or progressive)	13.14	36.6	Yes	97.9		0	2+ ENTC	
	06/03/15	Yes (not new or progressive)	11.88	36.2	N/A	97.1		0	4+ EC	
	07/03/15	No	9.25	36.7	N/A	98.1		0	3+ ENTC	
	08/03/15	No	10.19	N/A	N/A			N/A	N/A	
	09/03/15	N/A	N/A	N/A	Yes			N/A	N/A	
215	09/03/15	N/A	N/A	39.3	Yes	102.8		N/A	N/A	
	10/03/15	No	19.35	38.8	N/A	101.9		0	0	
	11/03/15	No	17.37	37.9	N/A	100.3		N/A	N/A	
	12/03/15	No	18.29	37.9	N/A	100.2		N/A	N/A	
	13/03/15	N/A	N/A	38.2	Yes	100.7		N/A	N/A	
216	10/03/15	No	14.91	37.4	Yes	99.3		0	0	
	11/03/15	Yes (new)	11.43	37.6	Yes	99.7	VAP	0	0	
	12/03/15	N/A	21.83	37.4	Yes	99.3		0	0	
	13/03/15	Yes	14.17	37.7	N/A	99.8		N/A	N/A	
	14/03/15	N/A	14.17	36.9	N/A	98.5		N/A	N/A	
217	10/03/15	Yes (not new or progressive)	27.8	37.4	N/A			N/A	N/A	
	11/03/15	Yes (not new or progressive)	35.04	37.5	N/A			N/A	N/A	
	12/03/15	No	29.23	37.6	Yes			N/A	N/A	
	13/03/15	No	23.75	38.9	N/A	102.1		N/A	N/A	
	14/03/15	No	22.65	38.9	N/A	102.1		N/A	N/A	
	15/03/15	Yes (new)	24.95	39.5	Yes	103.1	VAP	N/A	N/A	
	16/03/15	Yes	23.77	37.0	N/A	98.6		0	0	
	17/03/15	Yes	19.12	37.5	Yes		VAP	N/A	N/A	
	18/03/15	Yes	16.82	37.6	N/A	99.7		0	0	
	19/03/15	No	12.94	37.7	Yes	99.9		N/A	N/A	
	20/03/15	No	10.05	37.8	N/A	100		0	0	
	21/03/15	No	9.91	37.1	N/A	98.8		N/A	N/A	
218	15/03/15	No	15.4	38.0	Yes	100.4		N/A	N/A	
	16/03/15	Yes (new)	10.09	38.4	Yes	101.1		0	0	
	17/03/15	Yes	10.14	39.1	N/A	102.3		0	4+ HFLU	
	18/03/15	Yes	8.03	38.3	Yes	100.9		N/A	N/A	
	19/03/15	Yes	9.57	38.0	N/A	100.4		N/A	N/A	
	20/03/15	Yes	9.86	38.9	N/A	102		N/A	N/A	
	21/03/15	Yes	12	38.0	N/A	100.4		N/A	N/A	
	22/03/15	Yes	13.83	38.6	N/A	101.5		N/A	N/A	
	23/03/15	Yes	13	37.7	Yes	99.9		N/A	N/A	
	24/03/15	Yes	14.5	37.2	N/A	98.9		N/A	N/A	
	25/03/15	Yes	12.3	37.7	N/A	99.8		N/A	N/A	
219	15/03/15	No	26.22	39.2	No	102.6		N/A	N/A	
	16/03/15	No	20.9	39.5	No			0	0	
	17/03/15	No	11.91	37.7	No			0	0	
	18/03/15	No	10.4	37.7	N/A	99.8		0	0	

	19/03/15	Yes (new)	7.51	38.0	No	100.4		N/A	N/A	
	20/03/15	Yes (progressive)	8.86	38.4	No	101.2		0	0	
	21/03/15	Yes	9.51	38.7	Yes	101.6	VAP	0	0	
	22/03/15	Yes	8.85	37.4	N/A	99.3		0	0	
	23/03/15	Yes	7.26	37.8	No	100		N/A	N/A	
	24/03/15	Yes	5.54	37.2	No	99		0	0	
	25/03/15	Yes	5.66	37.3	No	99.1		0	0	
	26/03/15	No	6.41	37.7	N/A	99.8		0	0	
	27/03/15	No	5.25	37.3	N/A	99.1		N/A	N/A	
	28/03/15	No	6.01	37.2	N/A	98.9		N/A	N/A	
220	14/06/15	No	13.97	37.7	No	99.9		N/A	N/A	
	15/06/15	Yes (new)	17	37.7	No	99.8		0	0	
	16/06/15	Yes	13.96	37.3	No	99.2		0	0	
	17/06/15	Yes	14.05	37.8	No	100.1		0	0	
	18/06/15	Yes	12.99	37.5	No	99.5		0	4+ KP	
	19/06/15	No	10.72	37.1	Yes	98.7		0	0	
221	18/03/15	Yes (not new or progressive)	15.17	38.0	Yes	100.4		0	0	
	19/03/15	Yes (not new or progressive)	11.02	38.3	Yes	101		0	0	
	20/03/15	Yes (not new or progressive)	6.33	38.5	Yes	101.3		0	2+ EC	
	21/03/15	Yes (not new or progressive)	6.33	38.6	Yes	101.4		0	3+ EC	
	22/03/15	Yes (progressive)	8.17	38.5	Yes	101.3	VAP	0	3+ EC	
	23/03/15	Yes	5.39	38.4	Yes	101.2	VAP	0	4+ EC	
	24/03/15	Yes	8.02	38.9	N/A	102.1		N/A	N/A	
	25/03/15	Yes	8.05	37.9	Yes	100.3		N/A	N/A	
	26/03/15	No	8.25	37.3	N/A	99.1		2+	2+ EC	
	27/03/15	No	7.76	37.8	N/A	100		3+	3+ EC	
	28/03/15	Yes (new)	7.41	37.3	N/A	99.1		N/A	N/A	
	29/03/15	Yes	7.85	38.1	N/A	100.5		N/A	N/A	
	30/03/15	Yes	12.95	37.1	N/A	98.7		N/A	N/A	
222	19/03/15	Yes (not new or progressive)	15.72	38.1	No	100.6		N/A	N/A	
	20/03/15	Yes (not new or progressive)	21.98	37.5	No			0	0	
	21/03/15	Yes (progressive)	22	37.4	No	99.4		0	0	
	22/03/15	Yes	28.53	37.3	N/A	99.1		0	0	
	23/03/15	Yes	31.17	37.2	No	98.9		N/A	N/A	
	24/03/15	No	28.48	37.3	Yes	99.1		0	0	
	25/03/15	Yes (new)	32.8	37.6	No	99.7		0	0	
	26/03/15	Yes	25.08	37.4	N/A	99.4		0	0	
	27/03/15	Yes	18.83	37.1	N/A	98.8		N/A	N/A	
	28/03/15	Yes	14.87	37.3	N/A	99.2		N/A	N/A	
	29/03/15	Yes (progressive)	14.76	36.7	N/A	98		N/A	N/A	
223	21/03/15	Yes (not new or progressive)	24.44	37.7	Yes	99.9		N/A	N/A	
	22/03/15	Yes (not new or progressive)	7.3	37.8	No	100		3+	0	
	23/03/15	Yes (progressive)	6.89	37.7	N/A	99.8		2+	0	
	24/03/15	No	5.6	37.2	N/A	99		N/A	N/A	
224	22/03/15	Yes (not new or progressive)	14.81	38.8	No	101.9		N/A	N/A	
	23/03/15	Yes (not new or progressive)	14.63	38.1	No	100.5		2+	2+ S. rubidaea	
	24/03/15	Yes (progressive)	4.22	37.1	N/A	98.7		2+	2+ S. rubidaea	
	25/03/15	Yes	19.65	36.7	N/A	98		N/A	N/A	
	26/03/15	Yes	14.03	37.0	N/A	98.6		N/A	N/A	
	27/03/15	N/A	N/A	N/A	N/A			N/A	N/A	
225	22/03/15	Yes (not new or progressive)	20.3	39.1	Yes	102.3		N/A	N/A	
	23/03/15	Yes (not new or progressive)	11.79	37.5	No	99.5		0	0	
	24/03/15	Yes (not new or progressive)	10.58	37.2	Yes	98.9		2+	0	
	25/03/15	Yes (not new or progressive)	7.33	37.0	N/A	98.6		0	0	
	26/03/15	Yes (not new or progressive)	7.49	37.1	Yes	98.7		N/A	N/A	
	27/03/15	No	8.73	37.1	N/A	98.7		3+	0	
	28/03/15	Yes (new)	16.18	37.6	N/A	99.6		N/A	N/A	
	29/03/15	Yes	17.91	37.6	N/A	99.6		N/A	N/A	
	30/03/15	Yes	18.06	37.4	N/A	99.4		N/A	N/A	
	31/03/15	Yes	18.11	39.0	N/A	102.2	VAP	N/A	N/A	
	01/04/15	Yes	17.34	39.4	N/A	102.9	VAP	N/A	N/A	
	02/04/15	Yes	13.09	39.3	N/A	102.8	VAP	4+	0	
	03/04/15	Yes	10.85	39.6	N/A	103.2		3+	0	
	04/04/15	Yes	8.73	39.6	N/A	103.3		N/A	N/A	
	05/04/15	Yes	8.22	39.4	Yes	103	VAP	N/A	N/A	
	06/04/15	Yes	10.46	39.4	No	102.9		2+	0	
	07/04/15	N/A	7.82	37.4	N/A	99.3		0	0	
	08/04/15	N/A	3.18	38.3	Yes	100.9		N/A	N/A	
226	26/03/15	Yes (not new or progressive)	2.26	39.4	Yes	103		N/A	N/A	
	27/03/15	Yes (not new or progressive)	13.79	38.6	N/A	101.5		0	0	
	28/03/15	Yes (not new or progressive)	14.01	38.2	N/A	100.8		N/A	N/A	
227	16/06/15	No	7.79	38.9	No	102.1		N/A	N/A	
	17/06/15	No	7.06	38.0	No	100.4		0	2+ KP	
	18/06/15	No	6.28	37.5	N/A	99.5		N/A	N/A	
	19/06/15	No	3.96	37.2	Yes	98.9		N/A	N/A	
228	26/03/15	N/A	4.66	N/A	N/A			N/A	N/A	
	27/03/15	N/A	6.42	N/A	N/A			N/A	N/A	
	28/03/15	Yes (not new or progressive)	6.07	38.7	N/A	101.6		N/A	N/A	
	29/03/15	Yes (not new or progressive)	6	37.9	N/A	100.3		N/A	N/A	
	30/03/15	Yes (not new or progressive)	7.58	38.3	N/A	101		N/A	N/A	
	31/03/15	Yes (not new or progressive)	6.29	38.3	N/A	100.9		N/A	N/A	
	01/04/15	Yes (not new or progressive)	5.92	37.9	N/A	100.2		N/A	N/A	
	02/04/15	Yes (not new or progressive)	5.37	37.8	N/A	100		0	0	
	03/04/15	N/A	6.4	38.2	N/A	100.8		0	0	
	04/04/15	Yes (not new or progressive)	8.67	37.8	N/A	100		N/A	N/A	
	05/04/15	N/A	N/A	37.4	Yes	99.4		N/A	N/A	
	06/04/15	Yes (not new or progressive)	6.61	37.7	Yes	99.9		0	0	
	07/04/15	N/A	N/A	38.1	Yes	100.5		0	0	
229	06/04/15	Yes (not new or progressive)	20.77	37.2	No	99		0	2+ Mold	Yeast
	07/04/15	Yes (not new or progressive)	28.48	36.7	N/A	98.1		0	2+ Mold	
	08/04/15	N/A	N/A	N/A	N/A			N/A	N/A	
	09/04/15	N/A	N/A	N/A	No			N/A	N/A	
230	06/04/15	Yes (not new or progressive)	29.4	37.2	N/A	98.9		0	0	
	07/04/15	Yes (not new or progressive)	30.59	37.3	N/A	99.2		N/A	N/A	
	08/04/15	Yes (not new or progressive)	35.75	37.0	N/A	98.6		N/A	N/A	
	09/04/15	Yes (not new or progressive)	40.28	37.1	N/A	98.7		N/A	N/A	
	10/04/15	Yes (not new or progressive)	35.87	37.3	Yes	99.1		N/A	N/A	
	11/04/15	Yes (not new or progressive)	35.42	37.8	N/A	100		0	0	
	12/04/15	Yes (not new or progressive)	49.46	37.7	N/A	99.9		N/A	N/A	
	13/04/15	Yes (not new or progressive)	51.18	37.3	No	99.2		N/A	N/A	
	14/04/15	Yes (not new or progressive)	55.44	37.3	N/A	99.2		1+	0	
	15/04/15	Yes (not new or progressive)	46.55	37.2	N/A	99		N/A	N/A	
	16/04/15	N/A	51.14	37.1	N/A	98.7		N/A	N/A	
231	06/04/15	No	25.33	39.4	N/A	103		N/A	N/A	
	07/04/15	No	21.13	39.6	N/A	103.3		N/A	N/A	
	08/04/15	Yes (new)	29.02	37.0	N/A	98.6		N/A	N/A	
	09/04/15	N/A	N/A	N/A	N/A			N/A	N/A	
232	08/04/15	No	20.75	37.8	Yes	100		N/A	N/A	
	09/04/15	No	26.75	37.7	N/A	99.9		0	4+ MCAT	
	10/04/15	No	21.4	37.5	Yes	99.5		N/A	N/A	
	11/04/15	Yes (new)	16.16	37.2	Yes	99	VAP	0	0	
	12/04/15	Yes	23.31	37.3	No	99.1		0	0	
	13/04/15	Yes	40.02	37.2	Yes	99	VAP	0	0	

	14/04/15	Yes	45.49	37.3	N/A	99.1		0	0	
	15/04/15	Yes	41.13	37.8	N/A	100.1		0	0	
	16/04/15	Yes	34.97	37.9	Yes	100.2	VAP	N/A	N/A	
	17/04/15	No	36.98	37.1	N/A	98.7		N/A	N/A	
	18/04/15	No	35.46	37.4	No	99.4		0	0	
	19/04/15	No	30.53	38.2	N/A	100.8		0	0	
	20/04/15	No	25.12	37.4	N/A	99.3		N/A	N/A	
	21/04/15	No	23.43	37.9	N/A	100.2		N/A	N/A	
	22/04/15	No	20.67	37.9	Yes	100.2		N/A	N/A	
	23/04/15	Yes (new)	18.8	37.1	No	98.7		0	0	
	24/04/15	Yes	18.38	37.3	N/A	99.1		0	0	
	25/04/15	N/A	19	37.3	N/A	99.1		N/A	N/A	
233	08/04/15	Yes (not new or progressive)	4.77	36.9	Yes	98.4		N/A	N/A	
	09/04/15	Yes (not new or progressive)	3.04	37.2	Yes	99		0	0	
	10/04/15	Yes (not new or progressive)	3.66	37.2	No	98.9		0	0	
	11/04/15	No	3.98	37.8	Yes	100		1+	0	
	12/04/15	Yes (new)	6.05	38.0	Yes	100.4		2+	0	
	13/04/15	Yes	5.11	37.2	Yes	99		4+	0	
	14/04/15	Yes	7.16	37.2	Yes	98.9		4+	0	SAUR
	15/04/15	Yes	6.98	37.6	N/A	99.7		3+	0	
	16/04/15	No	6.49	37.3	Yes	99.2		N/A	N/A	
234	10/04/15	Yes (not new or progressive)	24.72	37.3	Yes	99.2		N/A	N/A	
	11/04/15	Yes (not new or progressive)	18.99	36.7	N/A	98		0	0	
	12/04/15	Yes (not new or progressive)	24.53	36.7	N/A	98		N/A	N/A	
	13/04/15	Yes (not new or progressive)	19.32	37.6	Yes	99.7		N/A	N/A	
	14/04/15	Yes (not new or progressive)	13.58	37.5	Yes	99.5		0	0	
	15/04/15	No	17.29	37.4	N/A	99.3		0	0	
	16/04/15	Yes (new)	31.05	36.7	N/A	98.1		N/A	N/A	
	17/04/15	N/A	N/A	N/A	Yes			N/A	N/A	
235	12/04/15	No	12.12	37.9	No	100.3		1+	0	
	13/04/15	No	10.53	37.7	N/A	99.9		1+	0	
	14/04/15	N/A	12.13	37.3	Yes	99.1		N/A	N/A	
236	14/04/15	No	41.79	37.5	N/A	99.5		1+	0	
	15/04/15	Yes (new)	20.65	37.5	Yes	99.5		0	0	
	16/04/15	Yes	14.55	37.2	N/A	98.9		N/A	N/A	
	17/04/15	Yes	15.65	36.8	Yes	98.2		N/A	N/A	
237	15/04/15	No	6.63	36.6	N/A	97.8		0	0	
	16/04/15	No	6.6	36.6	N/A	97.9		N/A	N/A	
	17/04/15	No	8.28	37.1	N/A	98.7		N/A	N/A	
	18/04/15	Yes (new)	5.88	36.8	N/A	98.2		N/A	N/A	
	19/04/15	Yes	5.88	37.0	N/A	98.6		N/A	N/A	
	20/04/15	Yes	7.28	38.0	N/A	100.4		N/A	N/A	
	21/04/15	Yes	8.28	37.6	Yes	99.6		N/A	N/A	
	22/04/15	No	N/A	37.9	N/A	100.3		0	4+ EC; 2+ PMIR	
	23/04/15	No	8.4	37.8	N/A	100		N/A	N/A	
	24/04/15	Yes (new)	9.43	37.9	N/A	100.2		N/A	N/A	
	25/04/15	Yes	11.03	38.4	N/A	101.2	VAP	N/A	N/A	
238	15/04/15	Yes (not new or progressive)	7.79	37.9	N/A	100.2		N/A	N/A	
	16/04/15	Yes (not new or progressive)	6.04	37.4	N/A	99.4		N/A	N/A	
	17/04/15	Yes (not new or progressive)	4.96	37.5	N/A	99.5		N/A	N/A	
	18/04/15	Yes (not new or progressive)	4.38	37.7	Yes	99.9		N/A	N/A	
	19/04/15	No	6.17	37.4	N/A	99.3		3+	0	
	20/04/15	No	9.85	37.6	N/A	99.6		N/A	N/A	
	21/04/15	No	8.61	37.0	N/A	98.6		N/A	N/A	
	22/04/15	Yes (new)	6.31	37.9	N/A	100.2		N/A	N/A	
	23/04/15	Yes	7.38	36.8	N/A	98.2		N/A	N/A	
	24/04/15	No	5.47	36.6	Yes	97.9		N/A	N/A	
	25/04/15	No	6.87	38.8	N/A	101.8		N/A	N/A	
	26/04/15	Yes (new)	15.8	39.1	N/A	102.4		N/A	N/A	
	27/04/15	Yes	9.67	37.3	Yes	99.1		N/A	N/A	
	28/04/15	No	6.47	36.9	No	98.4		1+	0	
	29/04/15	Yes (new)	7.67	37.1	Yes	98.8		2+	2+ XMAL	
	30/04/15	No	9.88	36.7	Yes	98		3+	3+ XMAL	
	01/05/15	No	13.02	36.7	N/A	98.1		0	3+ XMAL	
	02/05/15	No	26.11	37.4	N/A	99.4		N/A	N/A	
	03/05/15	N/A	N/A	N/A	Yes			N/A	N/A	
239	16/04/15	No	13.43	32.6	N/A	90.7		N/A	N/A	
	17/04/15	No	11.83	32.8	N/A	91		N/A	N/A	
	18/04/15	Yes (new)	9.5	37.7	N/A	99.8		N/A	N/A	
	19/04/15	Yes	11.27	37.8	N/A	100		N/A	N/A	
	20/04/15	Yes	10.94	37.9	N/A	100.2		N/A	N/A	
	21/04/15	Yes	12.02	37.8	Yes	100	VAP	N/A	N/A	
	22/04/15	No	9.57	37.4	N/A	99.3		0	0	
	23/04/15	Yes (new)	10.59	37.5	No	99.5		N/A	N/A	
	24/04/15	N/A	11.05	37.4	N/A	99.3		0	0	
	25/04/15	No	12.69	38.2	N/A	100.8		N/A	N/A	
	26/04/15	N/A	14.21	38.2	N/A	100.8		N/A	N/A	
	27/04/15	N/A	N/A	37.9	N/A	100.3		N/A	N/A	
	28/04/15	N/A	N/A	37.9	N/A	100.2		N/A	N/A	
	29/04/15	N/A	N/A	38.4	N/A	101.1		N/A	N/A	
	30/04/15	N/A	N/A	N/A	N/A			N/A	N/A	
240	16/04/15	No	7.97	38.7	N/A	101.7		N/A	N/A	
	17/04/15	No	N/A	38.4	N/A	101.1		N/A	N/A	
	18/04/15	Yes (new)	9.07	37.9	N/A	100.2		N/A	N/A	
241	16/04/15	Yes (not new or progressive)	21.5	38.1	N/A	100.5		N/A	N/A	
	17/04/15	Yes (progressive)	14.08	37.9	N/A	100.3		N/A	N/A	
	18/04/15	Yes	11.04	37.5	N/A	99.5		N/A	N/A	
242	13/05/15	N/A	21.29	38.9	Yes	102.1		N/A	N/A	
	14/05/15	Yes (not new or progressive)	18.92	38.9	No	102.1		0	0	
	15/05/15	Yes	15.96	38.1	No	100.5		0	0	
	16/05/15	Yes	13.98	37.6	No	99.7		0	0	
	17/05/15	Yes	15.34	37.2	Yes	99		1+	0	
	18/05/15	Yes	14.85	37.3	N/A	99.2		3+	0	
	19/05/15	No	14.21	36.9	N/A	98.4		N/A	N/A	
243	14/05/15	No	21.13	39.2	No	102.5		N/A	N/A	
	15/05/15	No	24.35	36.8	N/A	98.2		2+	0	
	16/05/15	N/A	N/A	N/A	Yes			N/A	N/A	
244	18/04/15	N/A	48.96	39.1	Yes	102.3		0	0	
	19/04/15	N/A	57.79	38.1	N/A	100.5		0	0	
	20/04/15	No	51.75	38.2	Yes	100.7		N/A	N/A	
	21/04/15	Yes (new)	47.72	38.7	N/A	101.6	VAP	0	0	
	22/04/15	Yes	50.56	38.4	N/A	101.2	VAP	N/A	N/A	
	23/04/15	Yes (progressive)	45.75	38.9	N/A	102.1	VAP	N/A	N/A	
	24/04/15	Yes	80.2	39.5	N/A	103.1	VAP	N/A	N/A	
	25/04/15	N/A	N/A	N/A	N/A			N/A	N/A	
245	20/04/15	No	13.25	37.9	N/A	100.3		N/A	N/A	
	21/04/15	No	13.59	37.5	Yes	99.5		N/A	N/A	
	22/04/15	Yes (new)	8.04	37.2	N/A	98.9		0	0	
	23/04/15	Yes (progressive)	7.39	37.3	No	99.2		N/A	N/A	
	24/04/15	Yes	7.22	37.5	N/A	99.5		0	0	
	25/04/15	Yes	9.49	37.4	N/A	99.3		N/A	N/A	
246	23/04/15	Yes (not new or progressive)	11.84	39.9	N/A	103.8		N/A	N/A	
	24/04/15	Yes (not new or progressive)	14.32	37.3	N/A	99.1		N/A	N/A	

	25/04/15	No	15.11	37.0	N/A	98.6		N/A	N/A
	26/04/15	No	20.64	36.7	No	98.1		N/A	N/A
	27/04/15	No	21.35	36.9	Yes	98.5		0	0
	28/04/15	No	17.09	40.0	No	104		0	0
	29/04/15	Yes (new)	14.1	40.0	Yes	104	VAP	0	0
	30/04/15	Yes	15.36	39.2	Yes	102.5	VAP	0	0
	01/05/15	Yes	14.65	37.4	Yes	99.4	VAP	0	0
	02/05/15	Yes	18.32	37.1	N/A	98.8		0	0
	03/05/15	No	23.17	36.9	N/A	98.4		N/A	N/A
	04/05/15	Yes (new)	26.27	37.8	Yes	100	VAP	N/A	N/A
	05/05/15	Yes	18.13	37.4	Yes	99.4	VAP	0	0
	06/05/15	No	13.62	37.2	N/A	98.9		0	0
	07/05/15	Yes (new)	13.45	37.3	N/A	99.1		N/A	N/A
	08/05/15	No	13.58	37.4	Yes	99.4		N/A	N/A
247	29/04/15	Yes (not new or progressive)	12.4	37.3	Yes	99.1		3+	0
	30/04/15	Yes (not new or progressive)	10.87	37.6	N/A	99.6		2+	0
	01/05/15	Yes (not new or progressive)	11.23	37.1	Yes	98.7		N/A	N/A
248	29/04/15	No	29.91	37.1	Yes	98.8		0	0
	30/04/15	Yes (new)	28.46	37.2	N/A	99		0	0
	01/05/15	N/A	37.61	36.8	N/A	98.3		N/A	N/A
249	07/05/15	Yes (not new or progressive)	16.17	37.6	Yes	99.7		N/A	N/A
	08/05/15	Yes (not new or progressive)	15.49	37.5	No	99.5		0	0
	09/05/15	No	7.26	37.4	No	99.4		0	0
	10/05/15	Yes (new)	6.27	37.3	Yes	99.2		0	0
	11/05/15	Yes	6.79	37.5	Yes	99.5		0	0
	12/05/15	Yes	6.94	38.6	Yes	101.4	VAP	0	0
	13/05/15	Yes	11.06	37.4	Yes	99.4	VAP	0	0
	14/05/15	Yes	11.19	37.4	N/A	99.4		0	0
	15/05/15	Yes (progressive)	10.85	37.1	Yes	98.8		N/A	N/A
250	08/05/15	Yes (not new or progressive)	12.84	37.6	Yes	99.7		N/A	N/A
	09/05/15	Yes (not new or progressive)	10.29	37.2	Yes	99		0	0
	10/05/15	Yes (not new or progressive)	8.62	37.7	No	99.8		0	0
	11/05/15	Yes (not new or progressive)	9.17	38.2	No	100.7		0	2+ XMAL
	12/05/15	No	9.18	38.8	Yes	101.9		0	2+ XMAL
	13/05/15	Yes (new)	10.96	37.7	Yes	99.9		0	3+ XMAL
	14/05/15	Yes	10.81	38.4	Yes	101.2	VAP	0	3+ XMAL
	15/05/15	Yes	11.44	38.1	No	100.6	VAP	0	3+ XMAL
	16/05/15	Yes	10.46	38.1	No	100.6		0	2+ XMAL
	17/05/15	Yes	8.6	37.8	Yes	100.1		0	4+ XMAL
	18/05/15	Yes (progressive)	9.29	38.0	Yes	100.4		0	3+ XMAL
	19/05/15	Yes	9.18	37.6	Yes	99.6		0	3+ XMAL
	20/05/15	Yes	9.71	37.9	Yes	100.2		0	4+ XMAL
	21/05/15	N/A	9.38	37.4	Yes	99.4		0	4+ XMAL
	22/05/15	Yes	8.31	37.6	Yes	99.7		3+	3+ XMAL
	23/05/15	N/A	9.46	37.6	N/A	99.6		3+	2+ XMAL
	24/05/15	N/A	9.84	37.7	N/A	99.9		N/A	N/A
