# Supplementary material for: The utility of endotracheal aspirate bacteriology in identifying mechanically ventilated patients at risk for ventilator associated pneumonia: a single-center prospective observational study
Source: BMC Infect Dis. 2019 Aug 29;19:756. doi: 10.1186/s12879-019-4367-7 (PMC6716855; doi:10.1186/s12879-019-4367-7)
Supplement: Supplementary file 9 — Methicillin resistance of the received S. aureus isolates. The data for the entire study population presented. (PDF 34 kb) [file 12879_2019_4367_MOESM9_ESM.pdf]

ETA Isolate	
study ID	Restistance MRSA / MSSA
002	MSSA
003	MSSA
014	MRSA
014	MRSA
014	MRSA
014	MRSA
021	MSSA
022	MRSA
022	MRSA
022	MRSA
022	MRSA
022	MRSA
023	MSSA
023	MSSA
030	MSSA
042	MSSA
042	MSSA
052	MRSA
053	MRSA
053	MRSA
055	MSSA
055	MSSA
058	MSSA
058	MSSA
058	MSSA
059	MRSA
059	MRSA
064	MRSA
072	MSSA
073	MSSA
073	MSSA
076	MRSA
77R	MSSA
77R	MSSA
77R	MSSA
77R	MSSA
078	MSSA
078	MSSA
080	MRSA
081	MRSA
081	MRSA

085	MSSA
088	MSSA
088	MSSA
088	MSSA
088	MSSA
088	MSSA
088	MSSA
088	MSSA
088	MSSA
088	MSSA
088	MSSA
088	MSSA
088	MSSA
090	MSSA
090	MSSA
090	MSSA
090	MSSA
090	MSSA
090	MSSA
090	MSSA
092	MSSA
102	MSSA
103	MRSA
105	MRSA
105	MRSA
107	MSSA
107	MSSA
109	MRSA
109	MRSA
109	MRSA
109	MRSA
109	MRSA
112	MSSA
113	MRSA
116	MSSA
123	MRSA
123	MRSA
123	MRSA
125	MSSA
125	MRSA
125	MRSA
125	MRSA
125	MRSA
125	MRSA
125	MRSA
125	MRSA
125	MRSA
125	MRSA
125	MRSA
125	MRSA

125	MRSA
133	MSSA
133	MSSA
134	MSSA
134	MSSA
134	MSSA
134	MSSA
134	MSSA
134	MSSA
134	MSSA
134	MSSA
134	MSSA
134	MSSA
134	MSSA
134	MSSA
135	MSSA
137	MRSA
138	MSSA
140	MSSA
140	MSSA
140	MSSA
140	MSSA
140	MSSA
140	MSSA
140	MSSA
140	MSSA
140	MSSA
140	MSSA
140	MSSA
140	MSSA
140	MSSA
140	MSSA
140	MSSA
141	MSSA
141	MSSA
141	MSSA
144	MSSA
145	MSSA
145	MSSA
146	MSSA
146	MSSA
146	MSSA
146	MSSA
146	MSSA
146	MSSA
148	MSSA
151	MSSA
151	MSSA
152	MSSA
152	MSSA

161	MSSA
161	MSSA
162	MSSA
162	MSSA
165	MSSA
169	MRSA
169	MRSA
169	MRSA
173	MRSA
179	MSSA
179	MSSA
179	MSSA
182	MRSA
182	MRSA
184	MRSA
184	MRSA
187	MRSA
201	MSSA
201	MSSA
201	MSSA
208	MRSA
210	MRSA
210	MRSA
210	MRSA
221	MRSA
223	MRSA
223	MRSA
224	MSSA
224	MSSA
225	MRSA
225	MRSA
225	MRSA
225	MRSA
225	MRSA
230	MSSA
233	MSSA
233	MSSA
235	MSSA
235	MSSA
236	MSSA
238	MRSA
238	MRSA
238	MRSA
238	MRSA
242	MRSA
242	MRSA
243	MRSA
247	MSSA

247	MSSA
250	MRSA
250	MRSA
